# PNKP targeting engages the autophagic machinery through STING and STAT3 to potentiate ferroptosis and chemotherapy in TNBC

**DOI:** 10.1016/j.redox.2025.103775

**Published:** 2025-07-22

**Authors:** Avi Maimon, Pier Giorgio Puzzovio, Yaron Vinik, Gavriel-David Hannuna, Sara Donzelli, Daniela Rutigliano, Giovanni Blandino, Sima Lev

**Affiliations:** a Molecular Cell Biology Department, Weizmann Institute of Science, Rehovot, 76100, Israel; b Translational Oncology Research Unit, Department of Research, Diagnosis and Innovative Technologies, IRCCS Regina Elena National Cancer Institute, Rome, Italy

**Keywords:** Ferroptosis, TNBC therapy, PNKP, STING, STAT3

## Abstract

The polynucleotide kinase/phosphatase (PNKP) is a DNA repair enzyme possessing bifunctional DNA 3′-phosphatase and DNA 5′-kinase activities. It plays an important role in the rejoining of single- and double-strand DNA breaks and is considered as a potential therapeutic target for different cancer types. Here we show that PNKP is highly expressed in triple negative breast cancer (TNBC) and associated with poor prognosis and chemoresistance. Targeting of PNKP enhanced ferroptosis in TNBC, which was associated with increased labile iron pool and ROS and concomitantly decreased in intracellular glutathione, SCD1 and GPX4 levels. Transcriptomic profiling and mechanistic data indicate that PNKP targeting robustly enhances the lysosomal and the autophagic machinery by activating STING and concurrently inhibiting STAT3, thereby increasing ferritinophagy, intracellular iron level and modulating the expression of key ferroptosis regulators. Importantly, PNKP and STAT3 are rapidly phosphorylated, colocalize, and interact upon ferroptosis induction or doxorubicin treatment, the first line treatment for TNBC patients. Targeting PNKP together with doxorubicin synergistically inhibited the growth of TNBC in an animal model and of TNBC-patients derived organoids. These results offer a promising therapeutic combination for TNBC and highlight the clinical potential of PNKP targeting and ferroptotic death for TNBC therapy.

## 1 Introduction

Despite the recent progress in targeted- and immuno-therapy, chemotherapy remains the mainstay treatment for triple negative breast cancer (TNBC), a highly aggressive and heterogenous breast cancer subtype [1]. TNBC is defined by the absence of estrogen and progesterone receptors and of HER2 amplification and is characterized by poor prognosis and high metastatic rate. Standard chemotherapeutic agents such as taxanes (mitotic inhibitors) and anthracyclines (DNA intercalators) are typically applied either in neoadjuvant or adjuvant settings [2]. Although a subset of TNBC patients can benefit from these chemotherapy treatments, approximately 30–50 % of the patients evolve chemoresistance and overall poor survival [3,4], highlighting the clinical need of new therapeutic strategies and potent combination therapies.

Doxorubicin, an anthracycline antibiotic, is possibly the most common anti-cancer chemotherapeutic agent used against a wide range of human cancers, including TNBC [5]. It is a quinone compound- implicated in reactive oxygen species (ROS) production, which can damage - cellular membranes, DNA and proteins. In addition, it has the capacity to intercalate into the DNA and interfere with topoisomerase II activity [6]. These activities not only damage cancer cells but are also associated with dosage-dependent cardiotoxicity and heart failure [7]. Extensive studies on doxorubicin-induced cancer cytotoxicity or cardiotoxicity suggest that doxorubicin may trigger various forms of regulated cell death (RCD), depending on the cell type, on the applied dose, and on the exposure time [8]. It appears that in addition to apoptotic cell death, doxorubicin can induce pyroptosis, necroptosis, and ferroptosis [9].

Ferroptosis is an RCD pathway driven by iron-dependent lipid peroxidation and severe damage of cellular membranes [10]. ROS, glutathione (GSH), labile iron pool (LIP) and polyunsaturated fatty acids (PUFAs) are fundamental for lipid peroxidation and ferroptosis execution [11,12]. Lipid peroxides are commonly metabolized into reactive aldehydes, such as 4-hydroxynonenal (4-HNE) and malondialdehyde (MDA), which react with cellular proteins and DNA to generate a variety of covalent adducts. The DNA adducts are associated with oxidative DNA damage, production of 8-hydroxydeoxyguanosine (8-OHdG) [13], and a variety of DNA lesions that engage the DNA damage response [13,14].

The major ferroptosis protective pathway in mammalian cells is the cyst(e)ine-GSH-GPX4 (Glutathione peroxidase 4) axis [15]. GPX4, a key ferroptosis protector, utilizes GSH as a cofactor to reduce lipid hydroperoxides, while GSH synthesis requires cystine import via the cystine/glutamate System Xc-antiporter. Two other key defense axes are the coenzyme Q10 (CoQ10)-FSP1(Ferroptosis suppressor protein 1) [16] and the tetrahydrobiopterin (BH4)-GCH1 (GTP cyclohydrolase-1) [17], which suppress lipid peroxidation independent of GPX4 activity. On the other hand, lipoxygenases (LOXs), which catalyze the dioxygenation of PUFAs and generate phospholipid peroxides (PLOOH) [18], ACSL4 (acyl-CoA synthetase long chain family member 4) and LPCAT3 (lysophosphatidylcholine acyltransferase 3), which are required for production of arachidonic acid (AA) and adrenic acid (AdA)-containing phosphatidylethanolamine (PE), the primary targets of lipid peroxidation, are all promoting ferroptosis execution.

Growing evidence suggests that ferroptosis vulnerability can be used for cancer therapy [19], and previously we showed that TNBCs are highly vulnerable to ferroptosis [20,21]. To exploit the ferroptotic susceptibility of TNBC, we developed a powerful computational pipeline to predict ferroptosis repressors as potential targets for TNBC therapy and identified PNKP as one of the top candidates [22]. PNKP is a DNA repair enzyme exhibiting dual phosphatase/kinase activities, which are required for repair of single- and double-strand breaks (SSBs and DSBs) by non-homologous end joining [23]. It is present both in the nucleus and in the mitochondria and involved in repair of oxidative DNA damage generated by ROS, ionizing radiation and certain chemotherapeutic agents [24,25].

Here we show that PNKP acts as a potent repressor of ferroptosis in TNBC. We identified the PNKP-STING-STAT3 axis as a regulator of ferroptotic cell death by converging the autophagic-lysosomal machinery with the DNA damage response. Our findings show that PNKP depletion simultaneously modulates the activities of cGAS-STING and STAT3 in opposing directions, potentially amplifying their individual effects through feedback loops. We also found that PNKP is a powerful therapeutic target for TNBC in combination with doxorubicin, a commonly used chemotherapeutic drug for TNBC patients, and propose that this synergistic combination could be a powerful approach to increase doxorubicin efficacy and possibly reduce its toxicity.

## 2 Results

### 2.1 Targeting of PNKP in TNBC induces lipid peroxidation and ferroptotic death

**Fig. 1:**
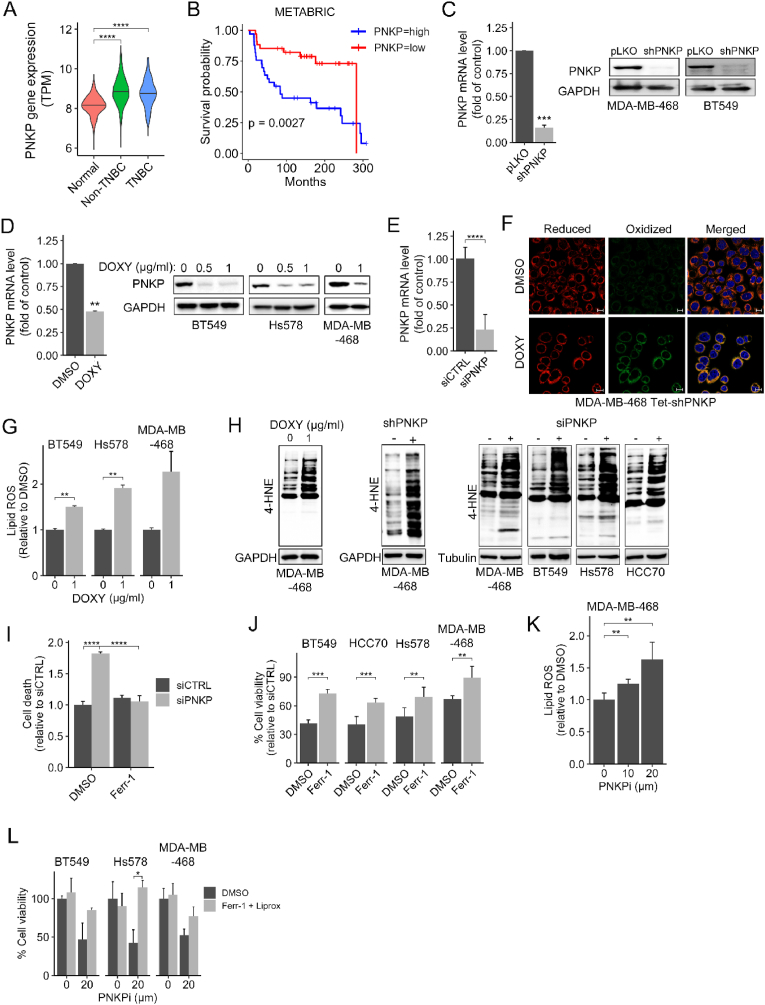
PNKP suppresses ferroptosis in TNBC. (A) The expression level of PNKP in non-TNBC and TNBC patients compared to healthy women was extracted from the TCGA datasets (n = 114 normal, 879 non-TNBC, 180 TNBC). (B) Kaplan-Meier curve for TNBC patients, stratified by their PNKP expression level (high and low PNKP indicates the 10 % patients with the highest and lowest PNKP expression, respectively) (n = 33 patients per group). (C–D) Knocking down (KD) of PNKP expression by the constitutive (C) or the Tet-inducible (D) shPNKP in the indicated TNBC cell lines were assessed by qPCR for PNKP transcripts (n = 3 in C, n = 2 in D, ∗∗∗∗p < 0.0001), or by Western Blotting (WB) for PNKP protein using anti-PNKP - antibodies. GAPDH was used as a loading control. p-values were determined by one-sample *t*-test. (E) KD of PNKP by siRNA (SMART pool) in MDA-MB-468 TNBC cell line was assessed by qPCR and compared to control (siCTRL) (n = 2 experiments in triplicates, ∗∗∗∗p < 0.0001). (F–H) Increased lipid peroxidation in PNKP-depleted cells. The indicated TNBC cell lines were depleted of PNKP either by siRNA, shRNA, or by the Tet-shRNA as indicated. Where indicated,The cells were treated with doxycycline (DOXY) (1 μg/ml) for 96 hr, and lipid peroxidation (lipid ROS) was assessed by BODIPY-C11 oxidation (F–G) or by WB to detect 4-HNE protein adducts (H). Representative confocal images of BODIPY‐C11 fluorescence are shown (F). Scale bar, 10 μm. Fluorometric quantification of lipid‐ROS (G) was calculated relative to control (0; DMSO) and the mean values ± SD from two independent experiments are shown. (I) Cells transfected with the indicated siRNA were grown in the absence or presence of ferrostatin-1 (3 μM) and cell death was measured by CellTox Green. Fluorometric quantification of cell death was calculated relative to control, and the mean values ± SD from two independent experiments are shown. (J) The indicated TNBC cell lines transfected with siCTRL or siPNKP were grown in the absence or presence of ferrostatin-1 (3 μM) and cell viability was measured 72 h later by MTT assay. Percentage (%) of cell viability was calculated compared to siCTRL. Shown are the mean values ± SD (n = 3). (K) MDA-MB-468 cells were treated with the indicated concentrations of PNKP inhibitor (A12B4C3, SIGMA) and lipid-ROS was assessed using BODIPY-C11 as described in G. Shown are the mean values ± SD from four independent experiments. (L) The indicated TNBC cell lines were treated with 20 μM of PNKP inhibitor in the presence or absence of a 1:1 mixture of liproxstatin/ferrostatin (3 μM) and cell viability was measured 72 hr later by MTT assay. Percentage (%) of cell viability was calculated compared to DMSO. Shown are the mean values ± SD from at least three independent experiments.

Considering the vulnerability of TNBC to ferroptosis, we developed different approaches, including a computational pipeline to identify potential ferroptosis repressors as candidates targets for TNBC therapy [21,22,26]. PNKP was one of the top candidates, and further analysis of its expression in the TCGA (The Cancer Genome Atlas) datasets revealed a significant higher expression in breast cancer patients compared to normal women (Fig. 1A), which was associated with poor prognosis in TNBC as demonstrated by the Kaplan–Meier curve (data from METABRIC, Fig. 1B).

To determine if PNKP indeed suppresses ferroptosis in TNBC, we depleted its expression by shRNA (constitutive and Tet-inducible shRNA) (Fig. 1C and D) or by siRNA (SMARTpool) (Fig. 1E–S1A) in several TNBC cell lines of different molecular subtypes (MDA-MB-468, BT549, Hs578, HCC70) and assessed lipid peroxidation by the fluorescent sensor BODIPY-C11 (Fig. 1F–G and S1B–F). As seen in the representative confocal microscopy images and by the fluorometric quantification - - (Fig. 1G–S1C, F), an increase in the oxidized form and the corresponding ratio between the oxidized (green) and reduced (red) forms were observed in PNKP knockdown (KD) cells. These results were further supported by an increase in 4-HNE protein adducts as assessed by Western Blot (WB) analysis using an antibody against 4-HNE, an aldehydic product of lipid peroxidation (Fig. 1H). The increased in lipid peroxidation led us to examine the influence of PNKP KD on ferroptotic death using cell death (Fig. 1I) and cell viability (Fig. 1J) assays in the absence or presence of the ferroptosis inhibitors ferrostatin and/or liproxstatin. As shown, PNKP KD reduced cell viability by ∼40–60 %, while ferrostatin and liproxstatin significantly increased the viability of PNKP-depleted TNBC cells (Fig. 1J–S1G, S1I). Likewise, depletion of PNKP significantly increased cell death as measured by CellTOX™ Green cytotoxic assay (Promega), while ferrostatin rescued the death response (Fig. 1I). Collectively, these results suggest that depletion of PNKP in TNBC cells induces lipid peroxidation and ferroptotic death.

To further validate the effects of PNKP KD, we investigated the impact of A12B4C3, a noncompetitive inhibitor of the phosphatase activity of PNKP , on lipid peroxidation (Fig. 1K) and ferroptotic death (Fig. 1L). While this inhibitor has been previously reported as a potent and selective for PNKP, with an IC_50_ of ∼60 nM in several human cancer cell lines [27,28], our findings show it to be less effective, exhibiting an apparent IC_50_ of ∼20 μM (Fig. S1H) in multiple TNBC cell lines. Nevertheless, treatment of TNBC cells with A12B4C3 (20 μM) for 12 h significantly increased lipid peroxidation as measured by BODIPY-C11 fluorescence (Fig. 1K), and enhanced ferroptotic cell death as measured by reduced cell viability 72 hr post treatment and rescue with ferrostatin and liproxstatin (Fig. 1L).

### 2.2 Depletion of PNKP modulates ROS, GSH and intracellular iron levels

**Fig. 2:**
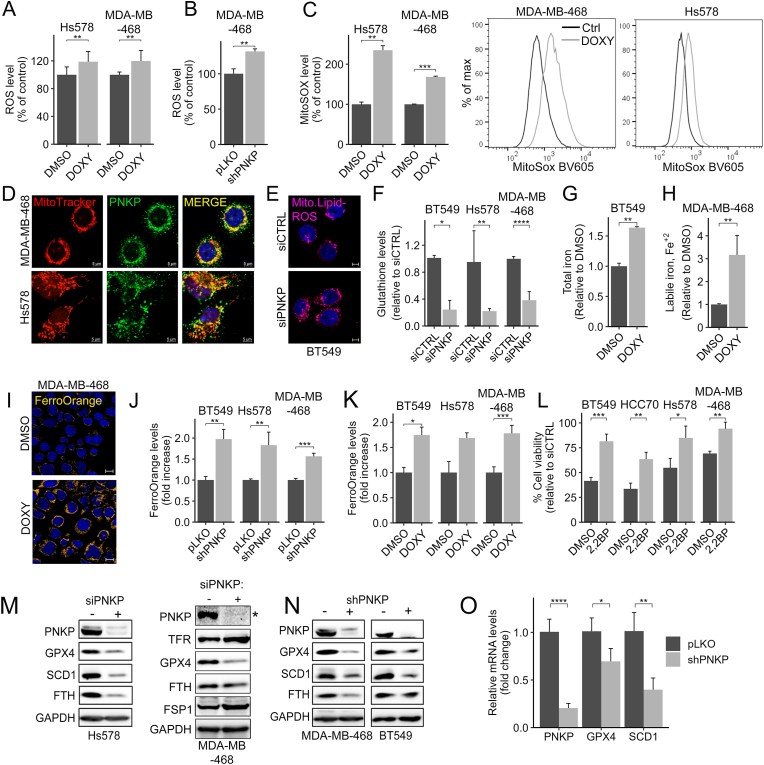
PNKP depletion modulates the levels of intracellular ROS, glutathione, iron, and key ferroptosis regulators. (A–C) Increased levels of cytosolic (A–B) and mitochondrial (C) ROS in PNKP-depleted cells. The indicated TNBC cells expressing the Tet-shPNKP were treated with either DMSO or doxycycline (DOXY) (1 μg/ml) for 4–5 days. Cells were then incubated either with CM-H_2_DCFDA to measure ROS as described in Methods or with MitoSOX to measure mitochondrial ROS using flow cytometry. Percentage (%) of ROS (A, B) and MitoSOX (C) in DOXY-treated cells were calculated relative to DMSO control cells. Shown are the mean values from 3 independent experiments done in duplicates (A), three experiments (B) and two experiments (C). (D) PNKP is localized in the mitochondria of TNBC cells. Shown are representative confocal images of the indicated TNBC cells stained with MitoTracker (red) and PNKP (green). Scale bar, 5 μm. (E) Increased mitochondrial lipid-ROS in BT549 cells depleted of PNKP (siPNKP) compared to control (siCTRL) cells was detected by MitoPeDPP staining. Shown are representative confocal images. Scale bar, 5 μm. (F–H) PNKP KD decreased intracellular levels of GSH (F), while increased total iron (G) and labile Fe^+2^ levels (H) as measured by colorimetric assays described in Methods. Shown are the mean values ± SD relative to control from 2 experiments done in duplicates (in F), and 2 or 4 independent experiments in G and H. (I) Representative confocal images of cells stained with the FerroOrange probe. Scale bar, 10 μm. (J–K) TNBC cells expressing either the constitutive KD (shPNKP) (J) or an inducible Tet-shPNKP (K) were treated with FerroOrange, fluorometric quantification was calculated as fold increase relative to control cells and the mean values ± SD are shown (n = 3 in J or 2 in K). (L) TNBC cells depleted for PNKP were grown in the absence or presence of the iron chelator 2,2-BP (8 μM) and 72 hr later, cell viability was measured relative to control cells (siCTRL). Shown are the percentage (%) of the mean values ± SD from three experiments. (M–N) The levels of the ferroptosis-related proteins GPX4, SCD1 and ferritin heavy chain (FTH) from cells transfected either with siCTRL/pLKO (−) as control or with siPNKP/shPNKP (+) were assessed by WB. GAPDH was used as a loading control. The blot marked with “∗” (PNKP) appears also in Fig. 5C, as both panels originate from the same Western blot experiment. (O) Transcripts of PNKP, GPX4 and SCD1 were measured by real time PCR, the mean values ± SD from three independent experiments are shown.

To further characterize the influence of PNKP on ferroptosis, we examined the effects of PNKP depletion (by siRNA and/or shRNA) on key ferroptosis modulators, including ROS, GSH, LIP and major regulatory proteins. To monitor intracellular ROS levels, we used the nonfluorescent H_2_DCFDA (2′,7′-dichlorodihydrofluorescein diacetate) probe, which is cleaved upon oxidation and converted into the highly fluorescent DCF (2′,7′-dichlorofluorescein) [29]. As seen in Fig. 2A and B, a significant increase in DCF fluorescence was obtained in PNKP KD compared to control cells. Since PNKP is also localized in the mitochondria (Fig. 2D–S2A), we examined the influence of its depletion on mitochondrial ROS using the MitoSOX Red Reagent and observed a significant increase as measured by flow cytometry (Fig. 2C–S2B). The increase in intracellular and mitochondrial ROS was accompanied by an increase in mitochondrial lipid peroxidation (Fig. 2E) and by a decrease in intracellular GSH level in PNKP depleted cells, as measured by colorimetric assay (Fig. 2F). We next examined the influence of PNKP depletion on intracellular iron level and LIP, using a colorimetric assay (Fig. 2G and H) and the FerroOrange probe (Fig. 2I–K, S2C). As shown, PNKP depletion significantly increased total iron and LIP in the different TNBC cell lines. Furthermore, the iron chelator 2,2′-Bipyridyl partially restored cell viability of PNKP KD cells (Fig. 2L), consistent with the rescue effects of ferrostatin or liproxstatin. These results indicate that PNKP KD elevated ROS levels both in the cytosol and in the mitochondria, decreased GSH and increased LIP levels.

We then assessed the influence of PNKP on key ferroptosis regulators by WB and real time PCR (qPCR). As seen in Fig. 2MN, S2D, PNKP KD reduced the protein level of GPX4, the major ferroptosis repressor as well as of ferritin heavy chain (FTH) and stearoyl-CoA desaturase-1 (SCD1), the rate limiting enzyme of monounsaturated fatty acids (MUFAs) synthesis. SCD1 markedly contributes to ferroptosis resistance by increasing MUFA levels, which exhibit antioxidant properties and indirectly influence PUFA levels [30]. The transcripts levels of GPX4 and SCD1 were also significantly reduced (Fig. 2O). Interestingly, we found a positive correlation between PNKP and GPX4 expression in all types of breast cancer including TNBC patients (Fig. S2E). Collectively, these results suggest that PNKP depletion leads to ferroptosis execution by modulation the levels of ROS, GSH, LIPs, GPX4, SCD1 and FTH.

### 2.3 PNKP depletion modulates the transcription of ferroptosis-associated pathways

**Fig. 3:**
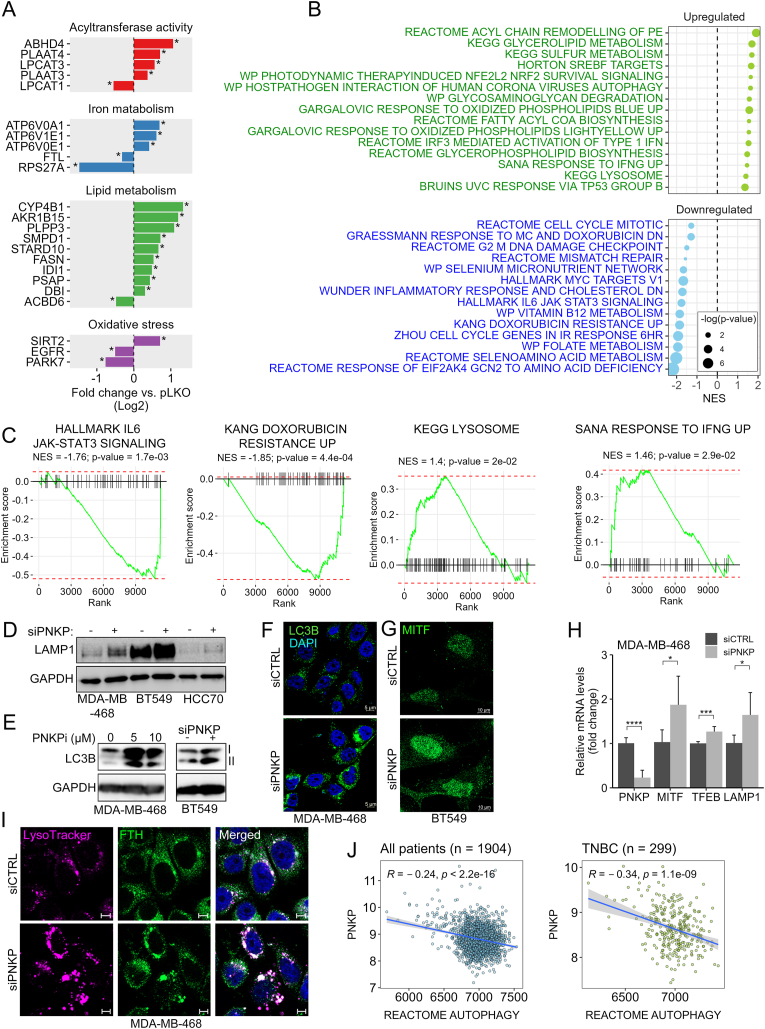
Transcriptomic profile of PNKP depleted cells and activation of the autophagy-lysosomal machinery. (A–C) Transcriptomic analysis of PNKP-depleted cells. Transcriptomic profiles of control (pLKO) and PNKP depleted (shPNKP) MDA-MB-468 cells were determined by RNAseq analysis as described in Methods. Differential gene expression between samples was quantified by Limma. Shown are some significantly differential genes related to ferroptosis (∗p-value <0.05). (B) Gene set enrichment analysis (GSEA) was performed based on the RNAseq results. Shown are selected pathways with significant (p-value <0.05) normalized enrichment score (NES). (C) GSEA plots for selected pathways. Normalized enrichment score (NES) and p-values are indicated. (D) The levels of LAMP1 in control (siCTRL, -) or PNKP depleted (siPNKP, +) TNBC cell lines were assessed by WB. GAPDH served as a loading control. (E, F). The levels of LC3B (I-cytosolic, II-lipidated, autophagosome) were assessed by WB (E) or by IF (F) in control cells or cells that were either treated with the indicated concentrations of PNKP inhibitor or depleted for PNKP. Shown are representative confocal images of LC3B staining following PNKP deletion (F). Scale bar, 5 μm. (G) Representative confocal images of MITF localization in control and PNKP depleted BT549 cells. Scale bar, 10 μm. (H) The levels of PNKP, MITF, TFEB and LAMP1 transcripts in control (siCTRL) and PNKP depleted (siPNKP) MDA-MB-468 cells were measured by qPCR. The mean values ± SD from two independent experiments are shown. (I) Increased co-localization of FTH with lysosomes after PNKP depletion. MDA-MB-468 cells transfected with the indicated siRNA were co-stained with lysotracker and FTH. Shown are representative of confocal images. Scale bar, 5 μm. (J) Correlation between PNKP expression and the enrichment of the autophagy pathway (measured by GSVA) in BC patients (n = 1904) and TNBC patients (n = 299) from MetaBric data.

To gain molecular insight into the ferroptotic death induced by PNKP KD, we profiled the transcriptomic response of PNKP KD in MDA-MB-468 by RNAseq. Differential gene expression (DGE) analysis revealed that 143 genes were significantly upregulated, while 184 genes were downregulated in PNKP-depleted cells compared to control. Many of the DEGs are related to ferroptosis (Fig. 3A), including critical lipid modifying enzymes, such as LPCAT3 (upregulated) and LPCAT1 (downregulated), which positively and negatively regulate PUFAs synthesis respectively [31]. Pathway enrichment analysis revealed upregulation of the IRF3 (Interferon regulatory factor 3)-IRF (Interferon)-STING (stimulator of interferon genes) pathway, consistent with previous reports [32,33]. Notably, we also observed upregulation of lysosomal and autophagy pathways (Fig. 3B), as well as oxidative phosphorylation and various lipid metabolism related pathways, particularly of phospholipids, including PE. The expression of selected genes related to these pathways, such as the lysosomal proton pumps ATP6V0E1 and ATP6V1E1 and the sulfotransferases SULT1A1/2, were further validated by qPCR (Fig. S3A). Downregulation of mitotic and cell cycle pathways, possibly due to accumulation of damaged DNA, were detected along with significant reduced levels of MYC targets, IL6/STAT3 signaling, selenium network, and doxorubicin resistance genes (Fig. 3B). Gene set enrichment analysis plots of selected pathways with the corresponding normalized enrichment score (NES) are shown in Fig. 3C–S3B.

Among the significantly upregulated pathways in PNKP depleted cells, the enrichment in the autophagy and lysosomal signatures led us to investigate the impact of PNKP depletion/inhibition on lysosomal activity and autophagy, both being involved in ferroptosis execution [34]. We observed upregulation in the lysosomal marker LAMP1 (lysosomal associated membrane protein 1) (Fig. 3D–S3D) and the autophagic protein LC3B (Microtubule-associated protein 1 light chain 3B), as shown by the immunofluorescence (IF) (Fig. 3F) and by the WB (Fig. 3E) analysis. We also observed increased intensity of LysoTracker (Fig. 3I–S3C), and its co-localization with ferritin (FTH) (Fig. 3I). These results suggest that PNKP KD enhances ferritinophagy, a process that promotes autophagic degradation of ferritin and the subsequent release of iron to upregulate LIP [35]. Consistent with these findings, we observed an upregulation in the mRNA levels of LAMP1 as well as of the autophagy-lysosomal transcription factors MITF and TFEB, both known to activate the autophagic pathway [36], and enhanced nuclear staining of MITF in various TNBC cell lines (Fig. 3G,H, S3E). The elevated activity of the lysosomal/autophagy pathway in PNKP-depleted cells is also reflected by the expression profiles of breast cancer patients, particularly of TNBC, where a negative correlation exists between PNKP expression and the autophagic pathway (Fig. 3J). Notably, stratification of breast cancer patients based on PNKP expression levels revealed a negative correlation between *PNKP* and *MAP1LC3B,* which encodes the LC3B autophagy protein (Fig. 3SF).

### 2.4 PNKP depletion induces DNA damage and STING-mediated autophagy

**Fig. 4:**
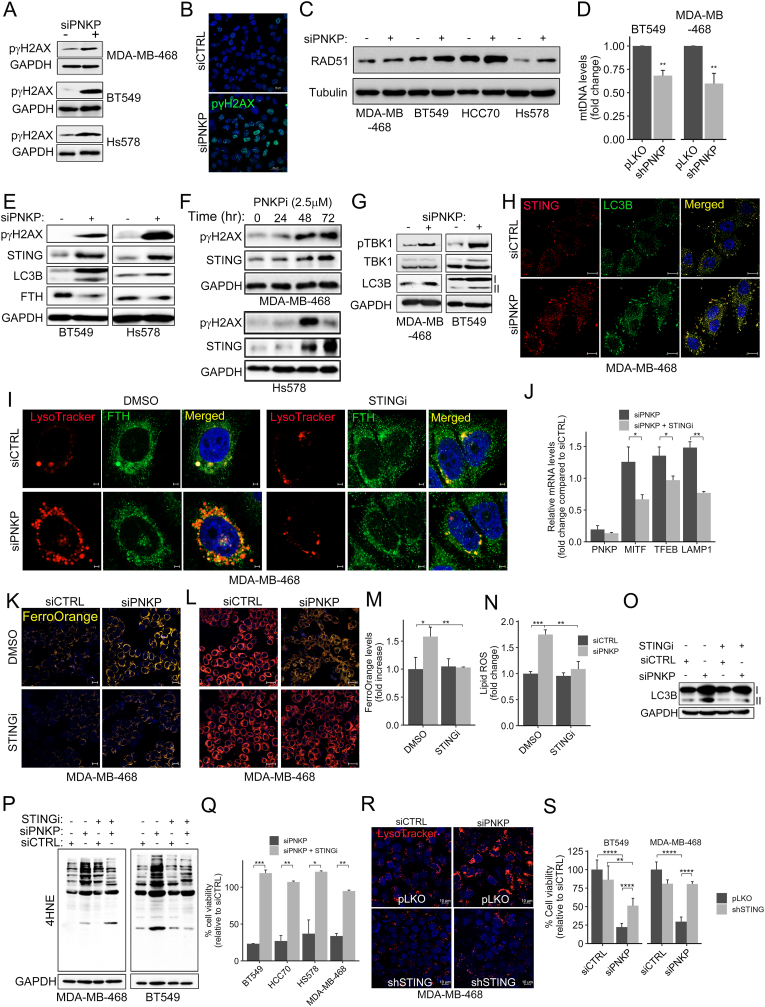
Increased DNA damage and STING-dependent autophagy upon PNKP deletion. (A–C) WB analysis demonstrating the influence of PNKP depletion (siPNKP) in different TNBC cell lines on DNA damage using antibodies against p-γH2AX (A) and RAD51 (C). GAPDH (in A) or Tubulin (in C) were used for loading control. (B) Representative confocal images for p-γH2AX staining in control and PNKP depleted MDA-MB-468 cells. Scale bar, 20 μm. (D) Increased mitochondrial DNA (mtDNA) damage in PNKP depleted (shPNKP) cells. Total DNA (genomic and mitochondria) was purified from the indicated TNBC cells and mtDNA fragments were amplified by qPCR as described in Methods. Shown are the mean values ± SD as fold change of control from four independent experiments. P-values were determined by one-sample *t*-test. (E–G) Increased DNA damage is associated with upregulation of STING and autophagy upon PNKP depletion. The indicated TNBC cell lines were either depleted of PNKP by siRNA (E, G) or treated with the PNKP inhibitor (PNKPi) A12B4C3 (F) and were analyzed by WB with the indicated antibodies. GAPDH was used for loading control. (H) Increased levels and co-localization of STING and LC3B following PNKP knockdown. Control (siCTRL) and PNKP depleted (siPNKP) MDA-MB-468 cells were co-stained for STING (red) and LC3B (green). Shown are representative IF images. Scale bar, 10 μm. (I–J) Inhibition of STING suppressed ferritinophagy in PNKP KD cells. Control (siCTRL) or PNKP depleted (siPNKP) MDA-MB-468 cells were incubated in the presence or absence of STING inhibitor (STINGi) C-170 (0.1 μM, 24 hr) and then analyzed either for ferritin accumulation in lysosomes (I) by co-staining with LysoTtracker and FTH as shown in the confocal images (Scale bar, 5 μm), or by qPCR (J) for autophagy/lysosomal-related transcripts. Shown are the mean values ± SD from two independent experiments. (K–Q) Inhibition of STING reduces LIP, lipid-ROS, autophagy and rescued cell viability of PNKP KD cells. Control (siCTRL) and PNKP depleted (siPNKP) MDA-MB-468 cells treated with STINGi were stained with FerroOrange (K, M), BODIPY-C11 (L, N) and processed by confocal microscopy (Scale bar, 10 μm), assessed for LC3B (O) and for 4HNE protein adducts (P) by WB, or for cell viability by MTT assay (Q). Cell viability (Q) and fluorometric quantification of K (in M) and of L (in N) were calculated relative to control (siCTRL) and the mean values ± SD from n = 3 (in M, N) and n = 2 (in Q) experiments are shown. (R–S) *STING* silencing suppresses the lysosomal activity and rescues cell viability of PNKP depleted cells. Control (pLKO) or STING depleted (shSTING) MDA-MB-468 cells were transfected with siCTRL or siPNKP and 72 hr later were stained with LysoTracker (R) and processed for confocal microscopy (Scale bar, 10 μm), or assessed for cell viability (S) by MTT. Cell viability was calculated relative to control (siCTRL) and the mean values ± SD from 2 experiments are shown.

Considering the strong effects of PNKP on the lysosomal-autophagic activities (Fig. 3B and 3D-I, S3A, C-E), its known DNA repair function [32,33] and the enrichment in IFN-STING pathway in PNKP KD cells (Fig. 3B and C), we assumed that PNKP may modulate the autophagic activity through the cGAS (cyclic GMP-AMP synthase)-STING pathway. To explore this possibility, we first demonstrated that PNKP depletion/inhibition indeed increased DNA damage using WB and IF analysis of phospho-histone H2AX (pγH2AX/Ser139) (Fig. 4A and B, E, F, S4A), as a marker for DNA double-strand breaks [37]. Elevated level of RAD51, which is involved in DNA DSBs repair [38], were also detected in PNKP KD cells (Fig. 4C), further demonstrating the impact of PNKP on DNA damage.

As PNKP localizes both in the nucleus and the mitochondria, and is involved in maintaining mitochondrial DNA (mtDNA) stability and integrity [25], we employed a qPCR-based approach as previously described [25,39] to monitor mtDNA damage. As shown in Fig. 4D, depletion of PNKP significantly reduced the levels of intact mtDNA. The elevated DNA damage observed in PNKP-depleted cells was accompanied by increased cytosolic double-stranded DNA (dsDNA) fragments (Fig. S4B). This was evidenced by enhanced anti-dsDNA antibody staining and the reduced co-localization with MitoTracker, suggesting leakage of DNA into the cytosol.

Previous studies have demonstrated a functional link between DNA damage and activation of the cGAS-STING pathway, which is involved in inflammation and an innate immune response and can also activate autophagy through different mechanisms, including direct interaction with LC3 [[40], [41], [42], [43]]. Indeed, we found that KD of PNKP (Fig. 4E) or inhibition of its activity by A12B4C3 (Fig. 4F) increased the level of STING1 in different TNBC cell lines and its colocalization with LC3B (Fig. 4H) as well as the level of LC3B-II, a marker of autophagosomes. We also observed elevated level of phospho-TBK1 (TANK-binding kinase 1) (Fig. 4G), which is recruited to activated STING and phosphorylates the transcription factor IRF3, leading to transcription of type I IFN, particularly IFN-β as previously reported [33]. These results suggest that the cGAS-STINGSTING pathway may contribute to the enhanced autophagic response observed in PNKP depleted cells, thereby promoting ferroptosis death. To explore this possibility, we examined the influence of C-170, a potent and covalent STING inhibitor [44] on autophagy/lysosomal activity and ferroptotic death in PNKP-depleted cells. As shown in Fig. 4I, C-170 significantly reduced the signal of LysoTracker staining and its co-localization with FTH in PNKP KD cells, suggesting reduced ferritinophagy. These results were accompanied by reduced levels of LAMP1, TFEB and MITF transcripts (Fig. 4J), in agreement with previous reports demonstrating the impact of cGAS-STING pathway on TFEB and lysosomal biogenesis [45]. C-170 also dimmed the FerroOrange signal of PNKP-depleted cells (Fig. 4K–M), suggesting a STING-dependent elevation of LIP in PNKP-KD cells. Furthermore, STING blockade significantly reduced lipid peroxidation in PNKP-depleted cells, as demonstrated by the level of oxidized BODIPY-C11 (Fig. 4L–N) and by the 4-HNE protein adducts (Fig. 4P). C-170 also decreased LC3B lipidation (Fig. 4O) and significantly restored cell viability of PNKP-KD cells (Fig. 4Q). Similarly, silencing of *STING1* expression by shRNA suppressed the ferroptosis-associated features of PNKP deficient cells. This was reflected by reduced lysosomal activity (Fig. 4R and S4D), decreased lipid peroxidation and LIP (Fig. S4E–F and S4G, H respectively) and a partial rescue in cell viability (Fig. 4S). Collectively, these results suggest that PNKP depletion elevates DNA damage, thereby activating the cGAS-STING pathway, which in turn modulates the autophagic machinery and enhances ferritinophagy and ferroptotic death.

### 2.5 STAT3 cooperates with PNKP to modulate autophagy-lysosomal and ferroptosis

**Fig. 5:**
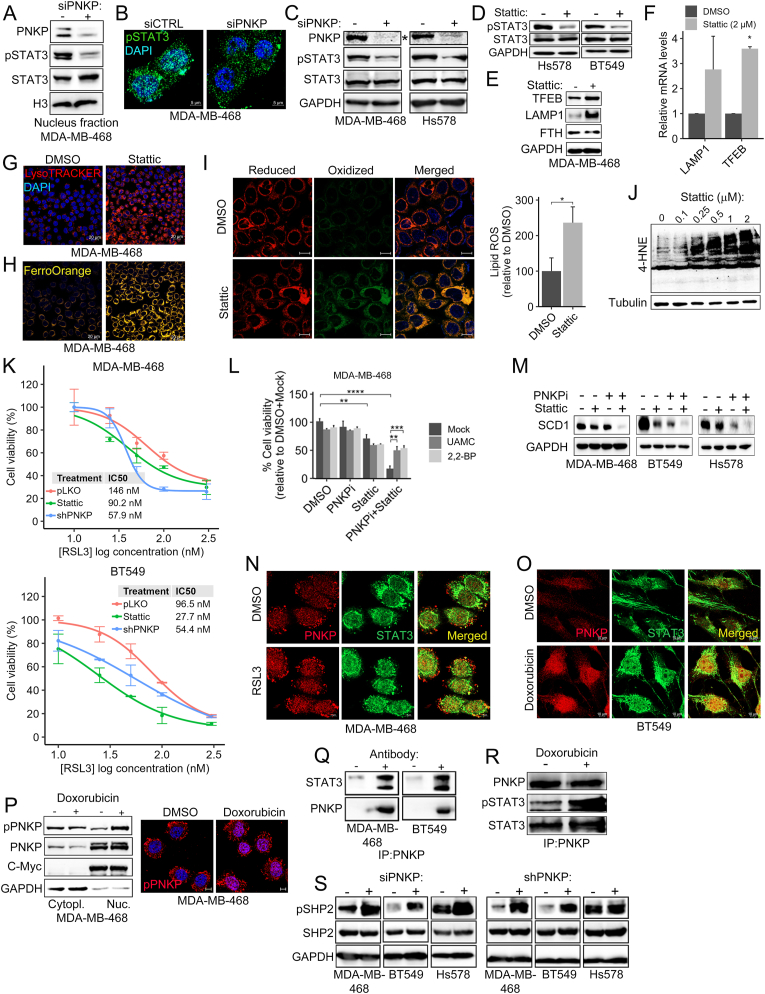
PNKP modulates STAT3 activity to enhance autophagy and ferroptosis. (A–B) Depletion of PNKP reduced the level of phospho-STAT3 (pY705). The levels of pSTAT3 in control (siCTRL) and PNKP depleted (siPNKP) cells were assessed either by cell fractionation (A) and WB using Histone 3 (H3) as loading control, or by IF (B) for pSTAT3. Scale bar, 5 μm. (C) Total cell lysates from control (siCTRL) and PNKP-depleted (siPNKP) TNBC cells were assessed by WB with the indicated antibodies. The blot marked with “∗” (PNKP) appears also in Fig. 2M, as both panels originate from the same Western blot experiment. (D–J) STAT3 inhibition enhances lysosomal activity, LIP and lipid ROS. Cells were treated with the STAT3 inhibitor Stattic (0.25 μM, 24 hr) and assessed by WB for STAT3 activation using anti-pSTAT3 (D) or the indicated autophagy-lysosomal markers (E). GAPDH was used as loading control (D, E). (F) The transcript levels of LAMP1 and TFEB were measured by qPCR. Shown are the mean values ± SD from two independent experiment (p-values measured by one-sample *t*-test). Cells were treated with Stattic (0.25 μM, 24 hr) and stained with LysoTracker (G), FerroOrange (H), or with BODIPY-C11 (I). Shown are representative confocal images. Scale bars, 20 μm (G, H), 10 μm (I). Fluorometric quantification of lipid-ROS was calculated relative to control (DMSO) and the mean values ± SD from three experiments are shown. (J) MDA-MB-468 cells were treated with increasing concentrations of Stattic (0.1–2 μM) and 20 hr later assessed by WB analysis for 4-HNE protein adducts. β-tubulin served as loading control. (K) Dose-response curve of RSL3 in the indicated control (pLKO), PNKP depleted (shPNKP), or Stattic-treated (0.5 μM) TNBC cell lines. MTT assay was used to measure cell viability 72 hr post treatment. Shown are mean percentage relative to DMSO ± SD from two independent experiments. (L–M) Co-inhibition of PNKP and STAT3 induced ferroptotic cell death. MDA-MB-468 cells (L) were treated with the indicated drugs in the absence or presence of iron chelator (2,2′BP, 8 μM) or UAMC-3203 (UAMC, 2.5 μM) and assessed 72 hr later for cell viability by MTT. Shown are percentage (%) of control with the mean values ± SD from three experiments. The indicated TNBC cells (M) were treated with the PNKP and/or STAT3 inhibitors for 24 hr and then assessed by WB using anti-SCD1 antibody. GAPDH was used for loading control. (N, O) PNKP-STAT3 interaction is enhanced during ferroptosis and/or doxorubicin treatment. Cells were treated either with RSL3 (N) or doxorubicin (O) were co-stained for PNKP (red) or STAT3 (green). Scale bar, 10 μm. (P) Increased phospho-PNKP after treatment with doxorubicin. Cell fractionation (left) and IF (right) were carried out 24 h post doxorubicin treatment and the levels of phospho-PNKP (pS114/T118) were assessed by WB. C-Myc and GAPDH were used as loading controls for nuclear (Nuc.) and cytoplasmic (Cytopl.) fractions respectively. Representative IF images of pPNKP staining (red) are shown, scale bar 10 μm. (Q, R) Co-Immunoprecipitation (Co-IP) of PNKP and STAT3. The indicated TNBC cells were subjected to immunoprecipitation (IP) with or without anti-PNKP (±) in steady state conditions (Q) or 24 hr post doxorubicin treatment (R). Shown are WB with the indicated antibodies demonstrating Co-IP of STAT3 with PNKP. (S) Enhanced SHP-2 activation in PNKP KD cells. Control and PNKP depleted (siRNA/shPNKP) TNBC cells were assessed for SHP-2 phosphorylation (pY542) by WB analysis. GPADH was used for loading control.

In addition to STING, it was previously shown that STAT3 also modulates autophagy in different cancer types through transcriptional regulation of autophagic-related genes and/or interaction with autophagic-related proteins [46]. It was also shown that STAT3 is highly active in TNBC and that TNBC are highly sensitive to STAT3 inhibition [47]. Furthermore, recent studies suggest that STAT3 inhibition may induce ferroptosis in certain (e.g. gastric cancer), but not all (e.g. pancreatic ductal adenocarcinoma) cancer types [48]. In line with these reports, and the downregulation of STAT3 pathway in the RNAseq data of PNKP-depleted cells (Fig. 3B and C), we examined the influence of PNKP depletion on STAT3 activation by WB and by IF analysis using anti-pSTAT3 (pY705) antibody. As shown in Fig. 5A–C, S5A the levels of pSTAT3 were markedly reduced in different PNKP-KD TNBC cell lines (MDA-MB-468, BT549, Hs578, HCC1937, HCC38) without an obvious effect on the total STAT3 level, suggesting that PNKP depletion impairs STAT3 activation. These observations led us to examine the impact of STAT3 inhibition on autophagy/lysosomal activity, using the small molecule STAT3 inhibitor Stattic [49]. As shown in Fig. 5D and E, treatment with Stattic reduced the levels of pSTAT3 and FTH proteins but increased the levels of LAMP1 and TFEB proteins, as well as pγH2Ax, STING and LC3B levels (Fig. S5F). The transcripts of LAMP1 and TFEB were also increased (Fig. 5F) as well as the intensity of LysoTracker staining (Fig. 5G) and of FerroOrange signal (Fig. 5H). These results indicate that, similar to the effects of PNKP depletion/inhibition, STAT3 inhibition also enhances lysosomal activity, ferritinophagy and increases LIP in TNBC. We, therefore, examined whether Stattic can also enhance lipid peroxidation in TNBC cells. Indeed, we found that treatment with Stattic (0.25 μM, 24 h) induced lipid peroxidation as determined by BODIPY-C11 staining (Fig. 5I) and by the level of 4-HNE-protein adducts as shown in the WB analysis of Stattic-treated cells (0.25–2 μM, 24 h) (Fig. 5J). In line of these results, we found that Stattic (0.5 μM) could sensitize TNBC cells to ferroptosis induced by RSL3, a canonical GPX4 inhibitor. As shown by the RSL3 dose response curve, Stattic significantly reduced the IC_50_ of RSL3 in MDA-MB-468 cells and even more profoundly in BT549 cells (Fig. 5K). Likewise, depletion of PNKP sensitized TNBC cells to RSL3 as well as to FIN56, which induces ferroptosis by triggering degradation of GPX4 [50] and by inhibiting GPX4 translation and Coenzyme Q10 production [51](Fig. 5K–S5D).

As both PNKP and STAT3 inhibition increased autophagic/lysosomal activity, lipid peroxidation, and sensitized TNBC cells to ferroptosis (Fig. 5K–S5D), we examined the impact of their co-inhibition on cell viability. We applied a dose of the PNKP inhibitor A12B4C3 (10 μM), which has no significant effect on cell viability (Fig. S1H), together with ∼IC_50_ concentration of Stattic, and found that the drugs combination drastically reduced cell viability in both MDA-MB-468 and BT-549 cells, which could be partially restored by the ferroptosis inhibitor UAMC-3203 and the iron chelator 2,2′-Bipyridyl (Fig. 5L and S5G, respectively). This strong effect of STAT3 and PNKP co-inhibition on cell viability was accompanied by an increase in the transcription levels of several autophagic genes, including *MAP1LC3B, TFEB, ATG14, ATG4B and BCN1*, which are essential for a autophagosome formation [52,53] (Fig. S5B). On the other hand, we observed a substantial decrease in the protein level of SCD1 (Fig. 5M) and in the transcription levels of key ferroptosis protecting genes, including GPX4 and SCD1 in response to PNKP and STAT3 co-inhibition compared to inhibition of either PNKP or STAT3 alone (Fig. S5B). In summary, although PNKP inhibition reduced STAT3 activation, we found that co-inhibition of STAT3 and PNKP provokes ferroptotic death. These results suggest that PNKP cooperates with STAT3 to protect TNBC cells from ferroptosis, possibly through their effects on autophagic/lysosomal activity and/or DNA damage. In line with this hypothesis, we found that ferroptosis inducers (RSL3, Erastin) enhanced the nuclear staining of pPNKP (Fig. S5E) shortly after treatment (5–10 hr), and concurrently the colocalization between PNKP and STAT3 (Fig. 5N). The elevated levels of nuclear pPNKP and PNKP-STAT3 colocalization were also observed in response to doxorubicin treatment for 24 hr (Fig. 5O–P, S5C), implying that the two proteins may interact. Indeed, co-immunoprecipitation (Co-IP) experiments (Fig. 5Q and R) suggest a physical interaction between STAT3 and PNKP. This interaction might protect STAT3 from dephosphorylation, thereby reducing STAT3 phosphorylation in PNKP-depleted cells (Fig. 5A–C, S5A). The interaction might also play a role in transcriptional regulation of specific targets genes and/or in DNA damage response [54]. Regardless the exact role of PNKP-STAT3 interaction, we found a significant increase in SHP-2 phosphorylation in PNKP KD TNBC cell lines (Fig. 5S). These results suggest that PNKP depletion leads to activation of the SHP-2 phosphatase [55], which in turn dephosphorylates its substrate pSTAT3 [56]. Collectively, we found that PNKP cooperates with STAT3, possibly through physical interaction and/or inhibition of SHP-2 activation, to protect TNBC cells from ferroptosis by regulating the autophagy/lysosomal machinery and the DNA damage response. This newly identified physical and functional interaction between PNKP and STAT3, together with previous reports on the crosstalk between the STAT3 and the cGAS-STING pathways [[57], [58], [59]], introduce the PNKP-STING-STAT3 axis as a new ferroptosis regulator, integrating the autophagy-lysosomal activities with the DNA damage.

### 2.6 Combination of PNKP targeting and doxorubicin reduced tumor growth in a xenograft mouse model and TNBC patients-derived organoids

**Fig. 6:**
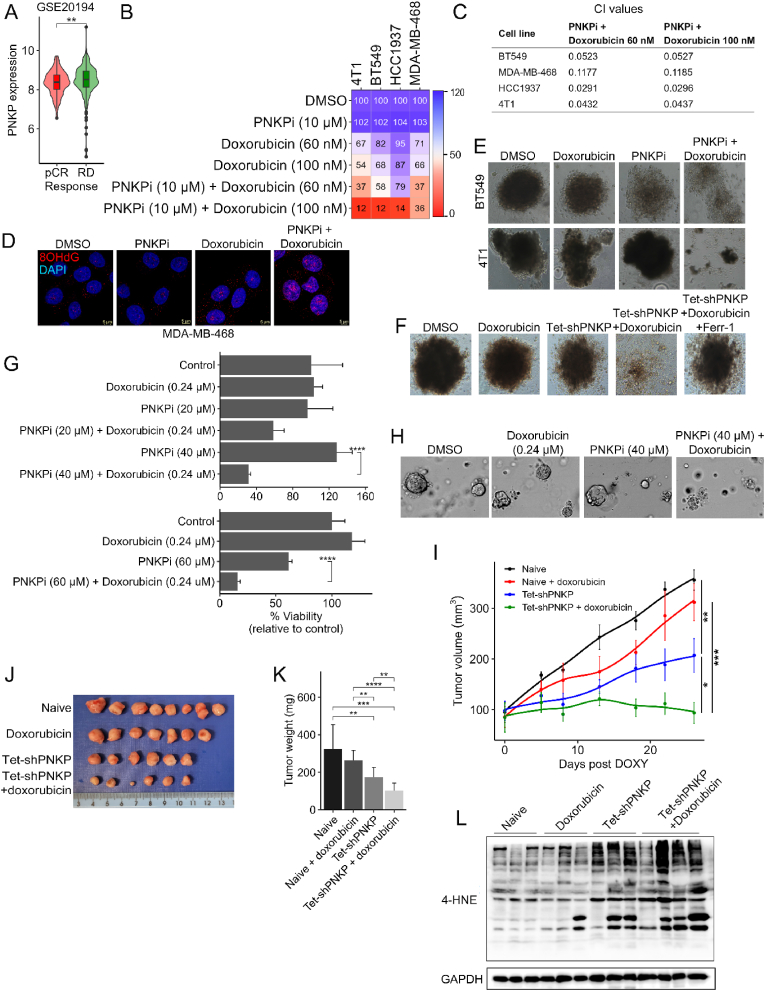
Elevated levels of PNKP in chemoresistant TNBC patients and its targeting together with doxorubicin suppress TNBC growth both in vitro and in vivo. (A) PNKP expression in TNBC patients, stratified by their chemotherapy response: pCR (complete response, n = 45) and RD (residual disease, n = 165). Data taken from GEO record GSE20194. (B) Heatmap representation of cell viability in response to the indicated drugs or drugs combinations in several TNBC cell lines. Cell viability was measured 72 hr post treatment and is shown as percentage of control (DMSO). Experiment done twice in duplicates, shown are means of the duplicates in one representative repeat. (C) Combination index (CI) for the combination between 10 μM PNKPi (A12B4C3) and 60–100 nM doxorubicin in the indicated cell lines was calculated according to the Bliss independence method. (D) Inhibition of PNKP (10 μM) potentiates the effect of doxorubicin (60 nM) on DNA oxidation as assessed by IF staining with anti-8OHdG antibody (red) 24 hr post treatment. Shown are representative confocal imagines. Scale bar, 5 μm. (E) BT549 or 4T1 cells grown as spheroids were either treated with PNKP inhibitor (60 μM) or with doxorubicin (360–600 nM) alone or with combination of the two drugs for 10 days. Shown are representative brightfield images. Scale bar, 100 μm. (F) Control or BT549 cells expressing Tet-shPNKP were grown as spheroids and treated either with DMSO as control, doxycycline (6 μg/ml) to induce PNKP KD, doxorubicin (360–600 nM), or doxycycline and doxorubicin in the absence or presence of ferrostatin-1 (18 μM). Brightfield images were acquired 15 days post treatment. Scale bar 100 μm. (G, H) Effects of single and drugs combination on TNBC patients derived organoids (PDOs) (G) were evaluated for viability assay 72 hr post treatment using the ATPlite luminescence assay as described in Methods. Shown are the mean percentage of viable organoids relative to control (DMSO) ± SD from two different PDOs (top and bottom), with at least 3 replicates per PDO. (H) Representative brightfield images showing the morphology of organoids after different treatments, scale bar 100 μm. (I–L) Depletion of PNKP together with doxorubicin treatment reduced tumor burden and increased lipid-ROS. Naïve or Tet‐shPNKP expressing MDA‐MB‐468 cells were implanted - into the mammary fat pad of female nude mice. When tumors reached ∼100 mm^3^, mice were randomized into four groups: Naïve or Tet‐shPNKP ± doxorubicin, *n* = 6–8 tumors/group. The two groups expressing the Tet-shPNKP were treated with doxycycline to knockdown PNKP. (I) Graph shows the mean tumor volume ± SEM in the indicated time points from non-induced (-doxycycline; naïve ± doxorubicin) and induced (+doxycycline; Tet‐shPNKP ± doxorubicin) tumors. p-values shown were measured by *t*-test comparing the volumes on day 26. 2-way ANOVA was performed to verify the significant difference between the groups (p-value = 5.11e-4) and the time points (p-value = 6.67e-31). (J–K) Image of excised tumors showing the reduced size after combining effect of PNKP depletion and doxorubicin treatment (J) along with end point (day 26) tumors weight (K) of the indicated groups. Shown are the mean mass of excised tumors ± SD (n = 6–8 per group). (L) Western blot analysis of control (naïve) and PNKP depleted tumors (Tet-shPNKP) ± doxorubicin (*n* = 3–4/group). Tumors were homogenized, lysed and analyzed for 4HNE‐protein adducts, and GAPDH was used as a loading control.

Although co-inhibition of STAT3 and PNKP significantly reduced cell viability (Fig. 5L–S5G), the high toxicity of Stattic [60] limits its clinical use for TNBC therapy. Doxorubicin, on the other hand, has long been used as a first line treatment for TNBC patients. In addition, the RNAseq data (Fig. 3B and C) indicates that the expression of doxorubicin resistance-related genes was reduced in PNKP KD cells, while PNKP expression was upregulated in chemoresistant TNBC patients (GSE20194) (Fig. 6A). These observations prompted us to examine the combined effects of PNKP inhibition and doxorubicin treatment on TNBC cells viability. We selected two fixed doses of doxorubicin (60 and 100 nM) that inhibit cell viability by 5–45 % across the different TNBC cell lines, and effectively low dose of PNKP inhibitor (10 μM). As shown in the dose-response matrix (Fig. 6B), the drugs combination substantially reduced cell viability, and exhibit strong synergy as reflected by the calculated combination index (CI)(Fig. 6C) using the Bliss independence method [61]. This synergistic effect was associated with elevated levels of DNA oxidation as demonstrated by IF analysis with anti-8-hydroxy-2-deoxyguanosine (8-OHdG) antibody (Fig. 6D). Notably, the synergy was obtained only for doxorubicin and not for paclitaxel or cisplatin (Fig. S6C), two chemotherapeutic drugs which are commonly used to treat the TNBC patients.

To further demonstrate the potency of this combination, we compared the IC_50_ values of doxorubicin in control TNBC cell lines to those of the PNKP KD (Tet-inducible shRNA) cells. The IC_50_ values were extracted from the doxorubicin dose-response curves and were reduced by ∼2.5 fold (Fig. S6A–B). Furthermore, this combination was also potent in 3 dimensional (3D) spheroids (Fig. 6E and F). We either used A12B4C3 (Fig. 6E) or the inducible PNKP KD (Fig. 6F) and observed robust effects of PNKP targeting together with doxorubicin on TNBC cell viability (4T1, BT549) 10 days post treatment as shown by representative brightfield images. Importantly, ferrostatin-1 restored cell viability of BT549 spheroids treated with doxorubicin together with doxycycline to downregulate PNKP (Fig. 6F).

To expand these observations, we examined the effects of the drugs combination on TNBC patients-derived organoids (PDOs). The organoids were established as described in Methods and treated either with doxorubicin (0.24 μM) or A12B4C3 (20 μM–60 μM) alone or with the drugs combinations. As shown, single treatments had no significant effects on cell viability, but the combined treatments of doxorubicin (0.24 μM) with PNKPi (40 and 60 μM) substantially suppressed the growth of PDOs from two different TNBC patients (Fig. 6G and H). Finally, we established xenograft models by orthotopic injection of the human MDA-MB-468 cells, either Naïve cells or cells expressing the inducible Tet-shPNKP into the mammary fat pad of nude mice. The tumors were grown for approximately two weeks to reach ∼100 mm^3^, and then half of the Naïve group was treated with vehicle, while the second half with doxorubicin to assess the influence of doxorubicin alone. Likewise, half of the Tet-shPNKP expressing group were treated with doxycycline to induce PNKP KD, while the second half with doxycycline and doxorubicin to assess the impact of the combination. The mice were treated for 28 days, and tumor volume was measured every 4–7 days by digital Vernier caliper. As shown in Fig. 6I, doxorubicin alone decreased tumor growth but not significantly, while PNKP KD had a stronger effect and significantly attenuated tumor growth. The combination of PNKP KD and doxorubicin had very strong effects on tumor growth. The tumors remained almost in the same volume throughout the treatment (Fig. 6I) and were significantly smaller at the end point as shown in the image of the excised tumors (Fig. 6J) and by their average mass (Fig. 6K). Interestingly, the excised tumors of PNKP-depletion or its combination with doxorubicin were enriched with 4-HNE-protein adducts as shown by the WB of representative tumors with anti-4-HNE antibodies (Fig. 6L), suggesting increase in ferroptotic death. Overall, this data indicates that PNKP targeting robustly increases the potency of doxorubicin, thereby introducing PNKP as a promising, clinically relevant, therapeutic target for TNBC therapy.

## 3 Discussion

In this study we show that targeting of PNKP in TNBC induces ferroptotic death (Fig. 1, S1) and potentiates the efficacy of doxorubicin (Fig. 6, S6A, B), suggesting that combining doxorubicin with PNKP blockade could improve the clinical outcome of doxorubicin treatment and may reduce its associated toxicity.

Targeting of PNKP was previously proposed to have a therapeutic potential in several human cancers, including lung, breast, colorectal, and prostate cancer, particularly in combination with γ-radiation or topoisomerase I inhibitor [62,63]. In prostate cancer cells, combination of PNKP inhibitor (A12B4C3) and carbon ion irradiation induced cell cycle arrest and apoptotic death [62]. In these cells, similarly to our data (Fig. 1K, L, S1H), a relatively high concentration of A12B4C3 (10–20 μM) was used at a micro rather than a nano molar range as previously reported [28]. These observations highlight the need for a highly potent and selective PNKP inhibitor.

Although additional small molecule inhibitors were developed (such as A83B4C63), they exhibit high toxicity towards normal cells [63]. Nevertheless, for the in vitro studies, A12B4C3 had similar effects as the PNKP KD (shRNA/siRNA) and induced ferroptotic death in several TNBC cell lines (Fig. 1L). The ferroptotic death induced by PNKP depletion/inhibition was accompanied by characteristic hallmarks of ferroptosis [64], including increase in lipid peroxidation, ROS, and LIP, concomitant with a decrease in GSH, GPX4, and SCD1 levels (Fig. 1F–L, S1B-G, 2M−O, S2D).

Through transcriptomic analysis and mechanistic studies, we found that PNKP depletion robustly enhanced the lysosomal/autophagy activities (Fig. 3B–I, S3A, S3C-E) and increased DNA damage response (Fig. 4A–F, S4A-B). The role of PNKP in DNA repair, especially of SSBs, and its implication in the pathology of several autosomal recessive neurodegenerative diseases, including AOA4 (Ataxia-ocular motor apraxia 4), CMT2B2 (Charcot–Marie–Tooth disease) and MCSZ (Microcephaly, seizures, and developmental delay) have been described in various studies [65].

**Fig. 7:**
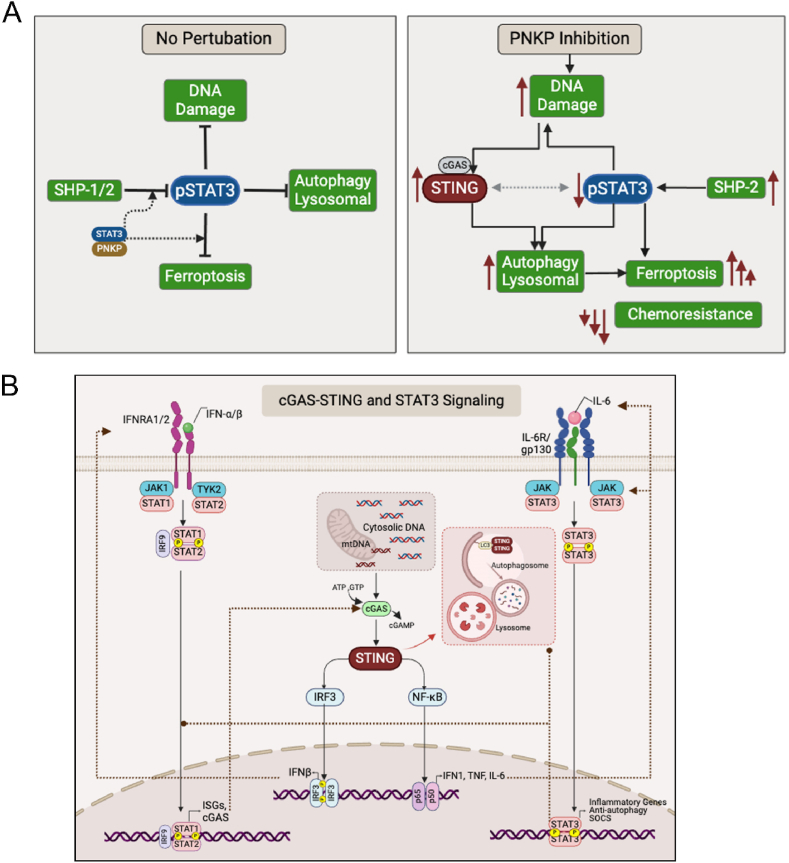
The PNKP-STING-STAT3 axis integrates autophagy/lysosomal machinery and DNA damage (A) An illustration depicting the impact of PNKP targeting on the STAT3 and cGAS-STING pathways, highlighting their crosstalk and feedback loops to potentiate ferroptosis and chemotherapy. Under baseline conditions of cancer cells (No Perturbation), pSTAT3 positively regulates tumor growth, while negatively regulates DNA damage response, the lysosomal-autophagic activities and ferroptosis. STAT3 activation is negatively regulated by the phosphatases SHP-1/2. We identified an interaction between PNKP and STAT3, which was transiently enhanced upon ferroptosis induction or doxorubicin treatment. This interaction might directly protect pSTAT3 from dephosphorylation and/or indirectly from ferroptosis through transcriptional modulation of ferroptosis protecting genes, such as GPX4, SCD1, FTH1, and of autophagic genes. Depletion/inhibition of PNKP disrupts the DNA repair machinery, leading to increased DNA damage and subsequent STING activation, which in turn enhances the autophagy-lysosomal activities. Simultaneously, the levels of pSTAT3 decrease, possibly due to SHP-2 activation and/or loss of physical protection. Decrease in pSTAT3 further enhances the autophagy-lysosomal machinery and the DNA damage, which potentiate the activity of STING to enhance ferritinophagy, lipid peroxidation, and ferroptotic death. This potentiation is mediated via positive feedback loops and contributes to the potent anti-tumor efficacy of PNKP targeting in combination with the chemotherapeutic drug doxorubicin. (B) The interplay between STAT3 and cGAS-STING pathways. The cGAS-STING (stimulator of interferon genes) pathway is involved in innate immune response and is activated by accumulation of cytosolic DNA fragments produced by DNA damage or microbial infections. The DNA fragments are sensed by cGAS (cyclic GMP-AMP synthase), which synthesizes cGAMP (cyclic GMPAMP) from ATP and GTP to activate STING. Activation of STING at the ER triggers its translocation to the Golgi apparatus and concomitantly the recruitment of TBK1 (TANK-binding kinase 1) and IKKα/β (Inhibitory kappaB kinase). TBK1 phosphorylates the transcription factor IRF3 (interferon regulatory factor 3), which in turn induces transcription of type I interferons (INFβ, α), while IKKα/β phosphorylates IkBα (inhibitor of nuclear factor kappa B), leading to release and activation of NF-kB. Activated NF-kB induces the transcription of inflammatory cytokines, such as TNF, IL-1β, and IL-6. Secreted IFNα/β activates their receptors IFNAR1/2, leading to JAK (Janus kinase)/TYK2 activation and phosphorylation of STAT1/2. The STAT1/2 dimer recruits IRF9 (interferon regulatory factor), translocate to the nucleus and triggers the transcription of ISGs (IFN-stimulated genes) including cGAS, demonstrating the positive feedback loops (dashed brown lines with arrow ends) of this pathway. IL-6 produced in response to NF-kB activation, binds to a cell-surface receptors complex composed of IL-6R and gp130, which in turn activates JAK1/2, leading to STAT3 phosphorylation (Y705), dimerization, and activation of transcription of inflammatory and anti-autophagic genes and of SOCS (suppressor of cytokine signaling), thus highlighting the crosstalk between the STAT3 and STING pathways. SOCSs act as negative feedback (dashed brown lines with circle ends) regulators by inhibiting JAK activity, preventing excessive signaling. STAT3 induces the expression of SOC3, which inhibits IFNα-induced STAT1 phosphorylation and consequently ISG expression, while suppressing the expression of STAT1, IRF7, and IRF9, further demonstrating the interplay between the two pathways. In addition to the role of STING in immune response, it activates autophagy by direct interaction with LC3 [40] and by activation of MITF [67], while STAT3 indirectly regulates autophagy and can suppress MITF [80].

However, its impact on the lysosomal/autophagy machinery and on ferroptotic death as described in this study was found to be mediated by its dual effects on the cGAS-STING and the STAT3 pathways, which are both involved in autophagy and DNA damage response (Fig. 7A). While the DNA damage response associated with STING function is mediated by cGAS and TBK axis to modulate antitumor and antiviral immunity [66], recent studies suggest that STING regulates lysosomal biogenesis and autophagy through TFEB activation in a TBK-independent manner [67]. TFEB activation and transcription of TFEB-targeted genes were also obtained in response to STAT3 inhibition in an ischemic stroke model [68], consistent with our results shown in Fig. 5E–F, S5B.

The STAT3 and STING pathways have also been implicated in ferroptosis regulation [69]. STING regulates ferroptosis through multiple mechanisms in a cell context dependent manner, and was reported to either inhibit or enhance ferroptosis [70,71]. In line with our results (Fig. 2M–O, S2D-E), recent studies suggest that STAT3 is upregulated in chemoresistant gastric cancer cells, and it directly binds to the promoter sequences of key negative regulators of ferroptosis, including GPX4, SCL7A11 and FTH1, thereby modulating their expression and protecting cells from ferroptosis [48].

Moreover, these two pathways are connected through several feedback loops. Previous studies showed that activation of STING by DNA damage in TNBC induced the expression of IL-6 and an autocrine activation of STAT3 [57]. However, our findings suggest that this feedback activation could be suppressed by PNKP depletion, which markedly reduces STAT3 activation (Fig. 5A–C, S5A). Other studies showed that STAT3 inhibition enhanced STING signaling induced by STING agonists and synergistically increased the anti-tumor immunity response in animal models of TNBC [58]. STAT3 inhibition also synergized with inducers of type I IFN, a downstream effector of the cGAS-STING pathway, to effectively inhibit B cell lymphoma growth, possibly by alleviating the suppressive effects of STAT3 on *IRF7*, *IRF9*, *STAT1*, and *STAT2* expression [72]. On the other hand, the two pathways are also connected through negative feedback loops mediated by the Interferon-stimulated genes (ISGs) and the SOCS (Suppressors of cytokine signaling), SOC1S and SOCS3. SOCS can suppress STAT1 and STAT3 activation, and consequently IFN-α mediated ISGs expression [73]. The interplay and crosstalk between STAT3 and cGAS-STING pathway is illustrated in Fig. 7B and highlights the feedback loops between the cascades.

The dual effect of PNKP depletion/inhibition on cGAS-STING activation and concurrently on STAT3 inhibition may increase the robustness of the response, possibly through inhibition/activation of feedback loops that eventually potentiate the response of STING activation and of STAT3 inhibition as shown in Fig. 7A. This may explain the synergistic effect of PNKP and STAT3 co-inhibition (Fig. 5K–M, S5B, G). We found that PNKP and STAT3 cooperate and possibly can protect cells from cell death induced by FINs or doxorubicin (Fig. 5K and L). The two proteins colocalize shortly after FINs or doxorubicin treatments and can be found in the same immunocomplex, as shown by Co-IP assay (Fig. 5N–O, S5C, 5Q, 5R). This interaction might prevent inactivation of STAT3 by SHP-2 or play a different role that needs to be further defined. Yet, the inactivation of STAT3 in response to PNKP inhibition can contribute to the synergistic effect with doxorubicin, as doxorubicin by itself induced DNA damage response, increased DNA oxidation, most profoundly together with PNKP inhibition (Fig. 6B–L, S6A-B), which can further enhance STING activation and STAT3 inhibition. Furthermore, previous studies reported that STAT3 inhibition and doxorubicin synergistically inhibited TNBC cells growth in vitro, reduced the expression of PD-L1 and activated immune cell-mediated cancer cell death [57], while other reports showed that SHP-2 activation enhances the activity of STING activation and chemotherapy response [74].

Altogether, we identified PNKP as a regulator of ferroptosis in TNBC and showed that PNKP is not only involved in DNA repair but also plays an important role in regulating the autophagic/lysosomal machinery by converging the cGAS-STING and STAT3 pathways, highlighting the impact of the PNKP-STING-STAT3 axis. In addition, we found that PNKP could be a promising target for TNBC therapy in combination with established and already approved chemotherapeutic drugs to increase effectiveness and reduce toxicity.

## 4 Materials & Methods

### 4.1 Cell culture

TNBC cell lines and human embryonic kidney HEK293T cells were obtained from the American Type Culture Collection (USA). HCC38 was obtained from M. Virginie (Institute Curie, Research Centre, Paris, France; 2014). MDA-MB-468, HCC1937, HCC70, HCC38, BT549, Hs578T and 4T1 cells were grown and maintained in RPMI medium (Gibco BRL, USA) containing 10 % fetal bovine serum (Gibco BRL, USA) and penicillin/streptomycin (100 U/ml, 100 μg/ml), while HEK293T cells were grown in Dulbecco's modified Eagle medium (DMEM), supplemented as mentioned above. Cells were cultured at 37 °C in a humidified incubator of 5 % CO2. Cell lines were routinely (once a month) checked for mycoplasma using a PCR assay.

### 4.2 Chemicals and drugs

STAT3 inhibitor (Stattic, sc-202818) was purchased from Santa Cruz Biotechnology. Liproxstatin-1 (17730), Ferrostatin-1 (SML0583), UAMC-3203 (26525), STING inhibitor C-170 (30157), doxorubicin (15007) and cisplatin (13119) were purchased from Cayman Chemical. Erastin (E7781), 2,2′-bipyridyl (D216305), A12B4C3 (PNKPi, A8736), doxycycline (D3072) and doxycycline hyclate (D9891), RSL3 (SML2234), FIN56 (SML1740), paclitaxel (T7402) and thiazolyl blue tetrazolium bromide (MTT) (M5655) were purchased from Sigma.

### 4.3 Antibodies

The antibodies list is provided in Supplementary Table 1 (Supporting Information).

### 4.4 Drugs treatments

For chemotherapy experiments: cells were seeded in 96-well plates and 24h later were treated with either PNKPi (10 μM), doxorubicin (60–100 nM), paclitaxel (1–3 nM), cisplatin (4–5 μM) or with combination of PNKPi and the above drugs. Control cells were treated with DMSO. Cell viability was assessed by MTT 72 hr later. For IF experiments, cells were treated with 60 nM doxorubicin in the presence or absence of PNKPi 10 μM for 24 hr and processed for IF following incubation with the drugs.

For PNKPi-Stattic combination signaling experiments: cells were treated with PNKPi and Stattic as single drugs (PNKPi 10 μM, Stattic 2 μM) or drugs combination for 24 hr. Cells were then processed for lysate preparation for WB or RNA extraction for qPCR.

For FIN sensitivity experiments: cells were seeded in 96-wells plates. After 24 hr, cells were treated with the indicated concentrations of RSL3 or FIN56 compared to DMSO-treated cells. 72 hr later, cell viability was measured by MTT assay. Results are presented as % cell viability of control untreated (DMSO) cells.

For cell viability rescue experiments: cells were seeded in 96-well plates and 24 hr later treated with the rescue compounds. For PNKPi viability rescue, cells were treated 24 h post seeding with PNKPi 20 μM. Cell viability was rescued by using either 3 μM ferrostatin-1, 3 μM liproxstatin-1, 2.5 μM UAMC-3203, 8 μM 2,2′-bipyridyl or 0.1 μM C-170. For experiments with siPNKP, cells were transfected with siRNA according to the manufacturer's protocol and 24 h later the rescuers were added to the cultures. Control cells were incubated with DMSO. Cell viability was measured by MTT assay 72 hr later. Results are presented as % cell viability of control untreated (DMSO) cells.

### 4.5 Flow cytometry

Cells were seeded in 12-well plates and 48–72 hr later incubated with the indicated fluorescent probe. The cells were then washed with PBS, trypsinized and centrifuged at 1200 rpm, for 4 min at 4 °C. Cells were washed twice with PBS and resuspended in 200 μl flow cytometry buffer (PBS + 0.1 % BSA). Fluorescence was acquired in a BD LSR II (BD Bioscience) (10 000 events/second) with the BV605 channel and the mean fluorescence intensity (MFI) was analyzed using FlowJo software.

### 4.6 ROS measurements

Levels of intracellular ROS were measured by the cell-permeable dye CM-H_2_DCFDA [5-(and-6)-chloromethyl-2′,7′-dichlorodihydrofluorescein diacetate, acetyl ester] (Invitrogen, LSC6827). Cells were cultured in 96-well black plates (Greiner, 655 090) for 16 hr and then treated with drugs as indicated. For total ROS levels cells were treated with 2 μM CM-H_2_DCFDA and Hoechst (1 mM) in Dulbecco's Phosphate Buffered Saline with Calcium and Magnesium (PBS^++^) (02-020-1A, Sartorius, Gottingen, Germany) for 10–15 min at 37 °C. Cells were washed twice with PBS^++^ and incubated in PBS^++^. Fluorescence of CM-H_2_DCFDA was measured by TECAN Infinite MPlex plate reader. CM-H_2_DCFDA fluorescence values were normalized by HOECHST values, indicative of the number of living cells in each well. Data is presented as percentage of ROS relative to control cells.

Mitochondrial ROS were detected by MitoSOX™ Red 396/610 nm fluorescent probe (M36008, ThermoFisher Scientific). MitoSOX™ Red (5 mM in DMSO stock) was diluted in serum-free RPMI to a final concentration of 2 μM and incubated with the cells for 15 min at 37 °C. Cells were then processed for flow cytometry as described above. Data is presented as percentage of MFI relative to control cells.

### 4.7 GSH measurement

GSH levels were measured by the Glutathione Colorimetric Assay kit (ab239709, Abcam, Cambridge, UK) according to the manufacturer's instructions and as previously described [21].

### 4.8 Generation of 3D spheroids

Spheroids were generated as follows. U-shaped 96-well plates (3799, Corning, NY, USA) were precoated with 20 mg/ml poly-HEMA (Poly-2-hydroxyethyl methacrylate, P3932, Merck-Millipore) in ethanol (60 μl per well) and left to dry overnight. Single-cell suspension of 4T1 or BT549 cells (7-8 × 10^3^ cells in 100 μl RPMI medium) was loaded into each well and plates were centrifuged at 1,000g for 15 min to allow formation of spheroid structures. Plates were then incubated for 2 days at 37 °C to form a single spheroid per well, and then treated with the indicated drugs for 10 days (spheroids were treated by fresh RPMI medium containing the indicated drugs every 3–4 days). Brightfield pictures of spheroids were acquired by an inverted microscope at × 10 magnification.

### 4.9 Cell viability and death assays

Cells were cultured in a 96-well plate and treated either with the indicated drugs, transfected with siRNA or induced with doxycycline. Cell viability was measured 72 hr later using the MTT (M2128, SIGMA) assay as described previously [75]. Results are presented as % cell viability of control untreated (DMSO) cells. For dose response curves, calculation of IC50 was performed using the drc package in R.

Cell death was measured by the CellTox-green Cytotoxicity assay (G8741, Promega) according to the manufacturer instructions. In brief, cells were seeded in 96 wells black plate (Greiner, 655 090) and treated as indicated for 72 hr. CellTox green reagent was diluted 1:1000 in assay buffer and added to the cells (100 μl/well), plates were immediately shaken for 1 min on an orbital shaker and then incubated in the dark at room temperature for 10–15 min. The fluorescence signal was measured by the Infinite 200 PRO Tecan fluorometric microplate reader at 490/525 nm (excitation/emission).

### 4.10 RNA extraction and Real‐Time PCR

Total RNA was extracted and purified using TRI Reagent (Sigma‐Aldrich) according to the manufacturer's instructions. RNA was reverse‐transcribed into complementary DNA (cDNA) using the High‐Capacity cDNA Reverse Transcription Kit (Applied Biosystems, Cat. No. 4368814) with random primers according to the manufacturer's instructions. Real‐time PCR analysis was performed in QuantStudio‐3 Real‐Time PCR system (Applied Biosystems, Thermo Fisher Scientific) using SYBR Green Master Mix reagents (Roche) using specific primers for the indicated genes. GAPDH was used as a housekeeping gene for normalization. Relative mRNA levels were calculated using the ΔΔ*C*_T_ method. The primers list is provided in Supplementary Table 2 (Supporting Information).

### 4.11 RNA sequencing

RNAseq was performed in independent duplicates on RNA extracted from control (pLKO) and PNKP KD (shPNKP) MDA-MB-468 cell line. Total RNA was extracted as described above. RNA quality was assessed using the Agilent 4200 TapeStation System (Agilent Technologies, Santa Clara, CA). RNA‐seq libraries were generated by applying a bulk adaptation of the MARS‐seq protocol, as previously described [76]. Libraries were sequenced by the Illumina Novaseq 6000 using SP mode 100 cycles kit (Illumina). Mapping of sequences to the genome and generation of the count matrix was performed by the UTAP pipeline (Weizmann Institute). Libraries normalization, filtration of low count genes, and discovery of differentially expressed genes were performed using the edgeR and Limma packages in R. Gene set enrichment analysis and GSEA plots were performed using the FGSEA package.

### 4.12 Gene knockdown

shRNA lentivirus–mediated knockdown: lentiviral vectors encoding shRNAs of PNKP (TRCN0000050367) and STING1 were purchased (TRCN0000161345) from Sigma and prepared as described previously [77].

For the Tet-ON inducible knockdown: The Tet-pLKO-puro lentiviral vector was purchased from Addgene (plasmid #21915; http://n2t.net/addgene:21915; RRID: Addgene_21915). The shRNA oligonucleotides described below were cloned into the Tet-pLKO-puro vector according to the manufacturer's instruction.

Oligonucleotide sequence for PNKP knockdown:

Forward:5′CCGGGAAGCGTATGCGGAAGTCAAACTCGAGTTTGACTTCCGCATACGCTTCTTTTTG-3′, Reverse:5′AATTCAAAAAGAAGCGTATGCGGAAGTCAAACTCGAGTTTGACTTCCGCATACGCTTC 3’.

Cells were transfected with the Tet-shPNKP construct, positive cells were selected with puromycin and incubated for 4–5 days with doxycycline (1 μg/ml) before experiments conduction.

For siRNA knockdown: PNKP siRNA (M-006783-02-0005, Dharmacon) was used to knockown PNKP, while a non-targeting siRNA (D-001210-05-05, Dharmacon) was used as control. Transfection of siRNA was performed using DharmaFECT Transfection Reagents (Dharmacon), following the manufacturer's instructions.

### 4.13 Cell lysates and Western Blotting

Cells were lysed in lysis buffer containing 0.5 % Triton X‐100, 50 mM Hepes (pH 7.5), 100 mM NaCl, 1 mM MgCl_2_, 50 mM NaF, 0.5 mM NaVO_3_, 20 mM β‐glycerophosphate, 1 mM phenylmethylsulfonyl fluoride, 10 μg/ml leupeptin, and 10 μg/ml aprotinin. For animals-derived tumors, tumor tissues were manually chopped with a sterile blade on dry ice, and 70 mg tissue was homogenized in 400 μl lysis buffer using 4 mm stainless steel beads in a homogenizer device (TissueLyser‐LT, QIAGEN) for 4 min. Cell lysates were centrifuged at 15 000 rpm for 15 min at 4 °C, and protein concentration of the supernatants was measured by Bradford assay (Bio‐Rad, Hercules, CA). Equal amounts of total protein (30–50 μg per sample) were analyzed by WB as previously described [77].

### 4.14 Iron measurement

Total (Fe^3+^ and Fe^2+^) and labile (Fe^2+^) cellular iron were measured using the colorimetric Iron Assay Kit (ab83366, Abcam, Cambridge, UK) as described previously [21].

### 4.15 Coimmunoprecipitation (Co-IP)

Co-IP was performed as described previously [78] with slight modifications. Briefly, cells were lysed in IP-Buffer (1 % NP-40, 20 mM Hepes, pH 7.5, 100 mM NaCl, 5 mM MgCl_2_, 1 mM PMSF, 10 μg/ml leupeptin, and 10 μg/ml aprotinin) and incubated on ice for 30 min. Supernatants were prepared by centrifuging cell lysates at 10 000 g for 15 min at 4 °C. 500 μg of protein sample was incubated with 0.5 μg of anti-PNKP antibody (sc-271505) and rotated over-night at 4 °C. For each sample, 30 μl of 50 % (v/v) protein A/G–PLUS-Agarose beads (sc-2003) were washed twice with PBS and then rotated for 1–2 h with IP-Buffer containing 1 % BSA for blocking. The pretreated beads were then mixed with the samples, rotated for 1 h at 4 °C and washed three times with IP-Washing Buffer (0.1 % Triton X-100, 20 mM Hepes, pH 7.5, 100 mM NaCl, 5 mM MgCl_2_, 1 mM PMSF, 10 μg/ml leupeptin, and 10 μg/ml aprotinin). Samples were then mixed with 2X Laemmli buffer, boiled and separated by SDS-PAGE. WB analysis with the relevant antibodies is described in the figure legends.

### 4.16 Cell fractionation

The nuclear and cytoplasmatic fractions were extracted from $1\times 10^{7}$ cells/group as we previously described in Ref. [21].

### 4.17 Fluorescence staining

Cells grown on glass coverslips in a 24‐wells plate were treated with drugs as indicated, washed twice with PBS, fixed in 4 % paraformaldehyde (PFA), and processed for IF staining essentially as we described previously [21].

Live cell imaging of lipid peroxidation, lysosomes, mitochondria or intracellular iron was performed with BODIPY-C11 (#27086, Cayman Chemicals), LysoTracker (Red DND‐99 Invitrogen), MitoTracker (M22425, Invitrogen) or FerroOrange (F374, DOJINDO) respectively, as described in 10.1002/advs.202307263. Lipid peroxidation quantification in live cells was performed by measuring the ratio between oxidized (green) and reduced (red) BODIPY-C11 normalized to Hoechst as previously reported [21]. Quantification of labile Fe^2+^ was performed as described in Ref. [21].

### 4.18 Mitochondrial DNA damage measurement

Mitochondrial DNA damage quantification was performed as previously described [39] with slight modifications. Cells were plated in 12-wells plate and 24 hr later cells were trypsinized, washed with PBS and cell pellets were stored at −80° for later processing. Total DNA was extracted using the DNeasy® Blood & Tissue Kit (Qiagen, Hilden, Germany). Real‐time PCR was performed with mitochondrial specific primers (Supplementary Table 2) using 6 ng of total DNA. qPCR was performed as described above. The relative levels of mtDNA were calculated using the ΔΔ*C*_T_ method by using nuclear actin as housekeeping gene, as described in Ref. [79]. DNA leakage in the cytoplasm was detected by IF using anti-dsDNA antibody. Cells were seeded in 12-well plates on coverslips and 24 hr later transfected with siCTRL or siPNKP. After 72 hr, cells were incubated with 70 nM MitoTracker for 30 min and processed for IF as described above. Localization of dsDNA within or outside the mitochondria was analyzed by using an anti-dsDNA antibody (1:25000) and Hoechst.

### 4.19 TNBC patients derived organoids (PDO)

Fresh tissues were obtained from patients with TNBC, treated in IRCCS-IRE Regina Elena National Cancer Institute. Collected samples were maintained in MACS Tissue Storage Solution (130-100-008, Miltenyi Biotec) supplemented with 100 U/ml penicillin, 100 μg/ml streptomycin and 100 μg/ml antimycotic for a maximum of 24 hr at 4 °C. Subsequently, the samples were washed twice in PBS, mechanically minced in a Petri dish, and the small pieces were transferred to a T75 flask or storage at −80 °C tissue into the cryotubes with 1 ml of MACS Freezing solution for each cryovial (130-129-552, Miltenyi Biotec). The single-cell suspensions were obtained using Tumor Dissociation Kit (130-095-929, Miltenyi Biotec), according to the manufacturer's instructions. The tissues were digested at 37 °C for 1 hr with occasional pipetting until the visible pieces disappeared. The obtained cellular suspension was then filtered with a 70 μm strainer (130-098, Miltenyi Biotec). Dissociated cell clusters were spun down at 1200 rpm for 5 min, washed once with PBS, and spun down again at 1200 rpm for 5 min. Erythrolysis was performed with Ammonium Chloride Solution (07850, STEMCELL Thecnologies) before the washing step. Dissociated cell clusters were resuspended in cold Matrigel (356231, Corning) and seeded in a prewarmed 24-well plate at density of 6 × 10^5^ cells per 30 μl drops. The drops were solidified in a 37 °C and 5 % CO2 incubator for 30 min, and then 500 μl organoid culture medium: Advanced DMEM/F-12 (12634010, Gibco), 200 mM GlutaMAX (35050038, Gibco), 1 mM HEPES (15630080, Gibco), 1X B27 supplement (17504001, Invitrogen), 1X N2 supplement (17502001, Invitrogen), Spondin 1 (HZ-1328, HumanKine), 100 ng/mL Noggin (HZ-1118, HumanKine), 5 nM Neuregulin (Qk045, HumanKine), 5 μM A83-01 (S7692, HumanKine), 50 ng/mL EGF (HZ-1326, HumanKine), 20 ng/mL FGF10 (Qk003, HumanKine), 5 ng/mL FGF7 (HZ-1100, HumanKine), 500 nM SB202190 (S7067, Sigma Aldrich), 5 μM Y-27632 (HY-10583, HumanKine) was added to each well and refreshed every 2–3 days. The organoids were passaged every 5–9 days, depending on proliferation rate.

### 4.20 Organoids treatment, viability assay and morphological parameters assessment

In all, 1 × 10^3^ cells were seeded into 96-well plates. PDOs were cultured in 3 μl of Matrigel and seeded into 96-well Pheno Plate (6055300, Revvity), using the Corning Matribot bioprinter. Allowed cells and organoids growth, they were treated for 72 h with 0.12–0.24 μM Doxorubicin (E2516); 20–40 μM A12B4C3 (A8736, Merck), drugs combination or DMSO. Cells and PDOs viabilities were assessed using ATPlite luminescence assay (Revvity, Massachusetts, USA) according to the manufacturer's instructions.

### 4.21 Animal studies

For PNKP mouse xenograft combination model, MDA‐MB‐468 cells were infected with Tet‐pLKO‐shPNKP lentivirus, selected with puromycin (1 μg/ml) and then implanted bilaterally into the mammary fat pads (2 × 10^6^ per gland) of the fourth inguinal gland of 5–6 weeks old female nude mice. When tumor size reached an average of ∼100 mm^3^, mice were randomized into four groups (*n* = 5 mice per group). Two naïve groups, *n* = 5 each; naïve ± Doxorubicin. Two Tet-shPNKP groups, n = 5 each; doxycycline ± Doxorubicin. Naïve mice received regular water, while the doxycycline experimental group (Tet-shPNKP) were treated with 2 mg/mL of doxycycline hyclate (D9891, SIGMA) in drinking water. Doxycycline was replaced every 3–4 days. At the second doxycycline administration, mice began receiving also doxorubicin intraperitoneally (4 mg/kg in HBSS buffer, 100 μl). Mouse body weights and tumor size were measured every 4 days. Tumor volume was measured by a digital Vernier caliper and calculated according to the (width^2^ × length)/2 formula. At the end of the experiment, mice were euthanized, tumors were excised, weighted and documented using a digital camera and processed for WB analysis.

### 4.22 Statistical analysis

Data are presented as means ± SD. To compare between experimental groups, we used Student's *t*-test (two-sided), unless otherwise stated in the figure legends.

## CRediT authorship contribution statement

**Avi Maimon:** Methodology, Investigation, Formal analysis, Data curation, Conceptualization. **Pier Giorgio Puzzovio:** Methodology, Investigation, Formal analysis, Data curation. **Yaron Vinik:** Formal analysis, Data curation. **Gavriel-David Hannuna:** Data curation, Formal analysis. **Sara Donzelli:** Data curation. **Daniela Rutigliano:** Data curation. **Giovanni Blandino:** Investigation. **Sima Lev:** Writing – original draft, Supervision, Project administration, Funding acquisition, Conceptualization.

## Declaration of competing interest

None.

## Ethics approval and consent to participate

All animal studies were performed according to protocols approved by the Weizmann Institutional Animal Care and Use Committee (IACUC approval number 05560723-2).

The Ethical Research Committee of IRCCS-IRE Regina Elena National Cancer Institute (Rome, Italy) approved this study. All patients gave written informed consent.

## Data availability statement

The RNAseq data performed for this study was deposited in the GEO repository (accession number GSE291269).

## Funding

This research was supported by the Ministry of Innovation, Science & Technology, Israel and by the Italian 10.13039/501100006601Ministry of Foreign Affairs and International Cooperation (Italy-Israel), the 10.13039/501100003977Israel Science Foundation (grant 1564/23), by “the Minerva foundation with funding from the Federal German Ministry for Education and Research”, by the Weizmann‐Pasteur joint research grant, by research grants from the Estate of Thomas Gruen and the Estate of Gertrude Buchler and by Roland N. Karlen foundation.

## Declaration of competing interest

The authors declare the following financial interests/personal relationships which may be considered as potential competing interests: Sima Lev reports financial support was provided by 10.13039/501100001735Weizmann Institute of Science. Giovanni Blandino reports financial support was provided by IRCCS Regina Elena 10.13039/100000054National Cancer Institute. Sima Lev reports a relationship with Weizmann Institute of Science that includes: employment. Sima Lev has patent No Patent pending to No License. No If there are other authors, they declare that they have no known competing financial interests or personal relationships that could have appeared to influence the work reported in this paper.

## Data Availability

Data will be made available on request.
