# Supplementary material for: PNKP targeting engages the autophagic machinery through STING and STAT3 to potentiate ferroptosis and chemotherapy in TNBC
Source: Redox Biol. 2025 Jul 22;86:103775. doi: 10.1016/j.redox.2025.103775 (PMC12336819; doi:10.1016/j.redox.2025.103775)
Supplement: Multimedia component 1 [file mmc1.docx]

**SUPPLEMENTARY INFORMATION**

**PNKP Targeting Engages the Autophagic Machinery through STING and STAT3 to Potentiate Ferroptosis and Chemotherapy in TNBC**

Avi Maimon^1*^, Pier Giorgio Puzzovio ^1*^, Yaron Vinik^1^, Gavriel-David Hannuna ^1^, Sara Donzelli^2^, Daniela Rutigliano^2^, Giovanni Blandino^2^, and Sima Lev^1†^

^1^Molecular Cell Biology Department, Weizmann Institute of Science, Rehovot 76100, Israel.

^2^Translational Oncology Research Unit, Department of Research, Diagnosis and Innovative Technologies, IRCCS Regina Elena National Cancer Institute, Rome, Italy.

*These authors contributed equally to this work

^†^Corresponding author: Sima Lev, Weizmann Institute of Science, Email: [Sima.Lev@weizmann.ac.il](mailto:Sima.Lev@weizmann.ac.il)

**Supplementary Figures and Legends**

**
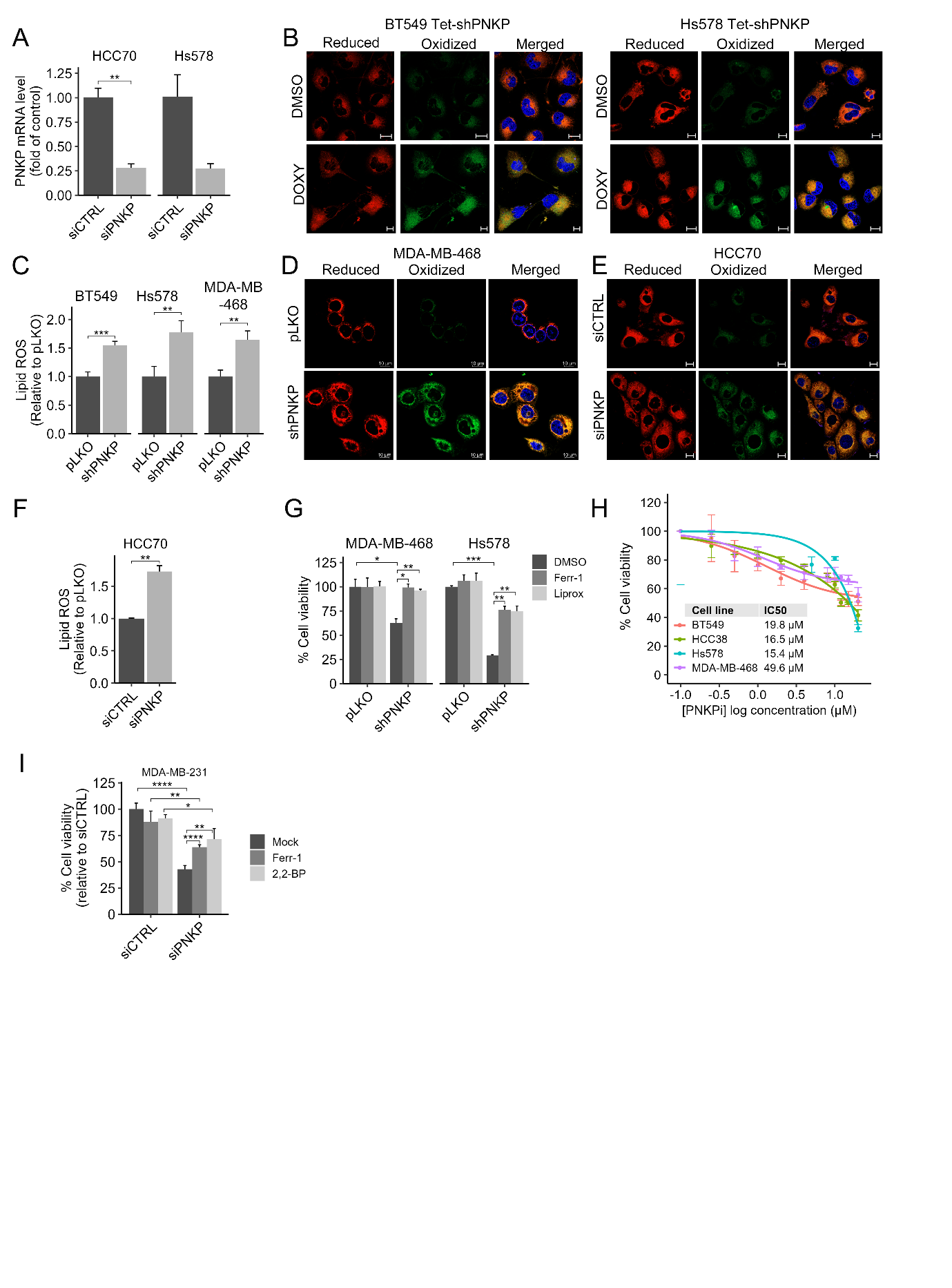
**

**Figure S1: PNKP targeting enhances lipid peroxidation and ferroptosis**

**(A)** Knocking down (KD) of PNKP by siRNA in the indicated TNBC cell lines was confirmed by qPCR. Shown are the mean values relative to control ±SD from two independent experiments. **(B, C)** The indicated TNBC cell lines expressing the inducible KD (Tet-shPNKP) (B), grown in the absence (DMSO) or presence of doxycycline (DOXY, 1 µg/ml), or expressing the constitutive shPNKP (C) and analyzed for lipid peroxidation by BODIPY-C11 staining. Representative confocal images of the reduced and the oxidized BODIPY-C11 fluorescence are shown in B. Scale bar, 10 µm. Fluorometric quantification of lipid‐ROS (C) was calculated relative to control pLKO and the mean values ± SD from three independent experiments are shown. **(D-F)** Lipid-ROS were assessed by BODIPY-C11 staining as described above in the indicated cells depleted of PNKP either by shPNKP (D) or by siPNKP (E). Representative confocal images of BODIPY-C11 fluorescence are shown (D, E). Scale bar 10 µm. Fluorometric quantification of lipid‐ROS (F) was calculated relative to siCTRL and the mean values ± SD from two independent experiments are shown. **(G)** Cells depleted of PNKP were grown in the absence or presence of ferrostatin-1 (Ferr-1, 5 µM) or liproxstatin (Liprox, 5 µM) and cell viability was assessed 72 hr later. Percentage (%) of cell viability relative to control (pLKO) is shown with the mean values ± SD from two independent experiments. **(H)** Dose response curve of PNKP inhibitor A12B4C3 (PNKPi) applied in the indicated cell lines and cell viability was calculated 72 hr later as described above. Shown are the mean values ± SD from two independent experiments. **(I)** MDA-MB-231 cells were transfected with siCTRL or siPNKP and 24 hr later were grown in the absence or presence of Ferr-1 (5 μM) or 2,2-BP (8 μM) for 72 hr. Cell viability was measured by MTT assay. Percentage (%) of cell viability relative to control (siCTRL) is shown with the mean values ± SD (n = 4).


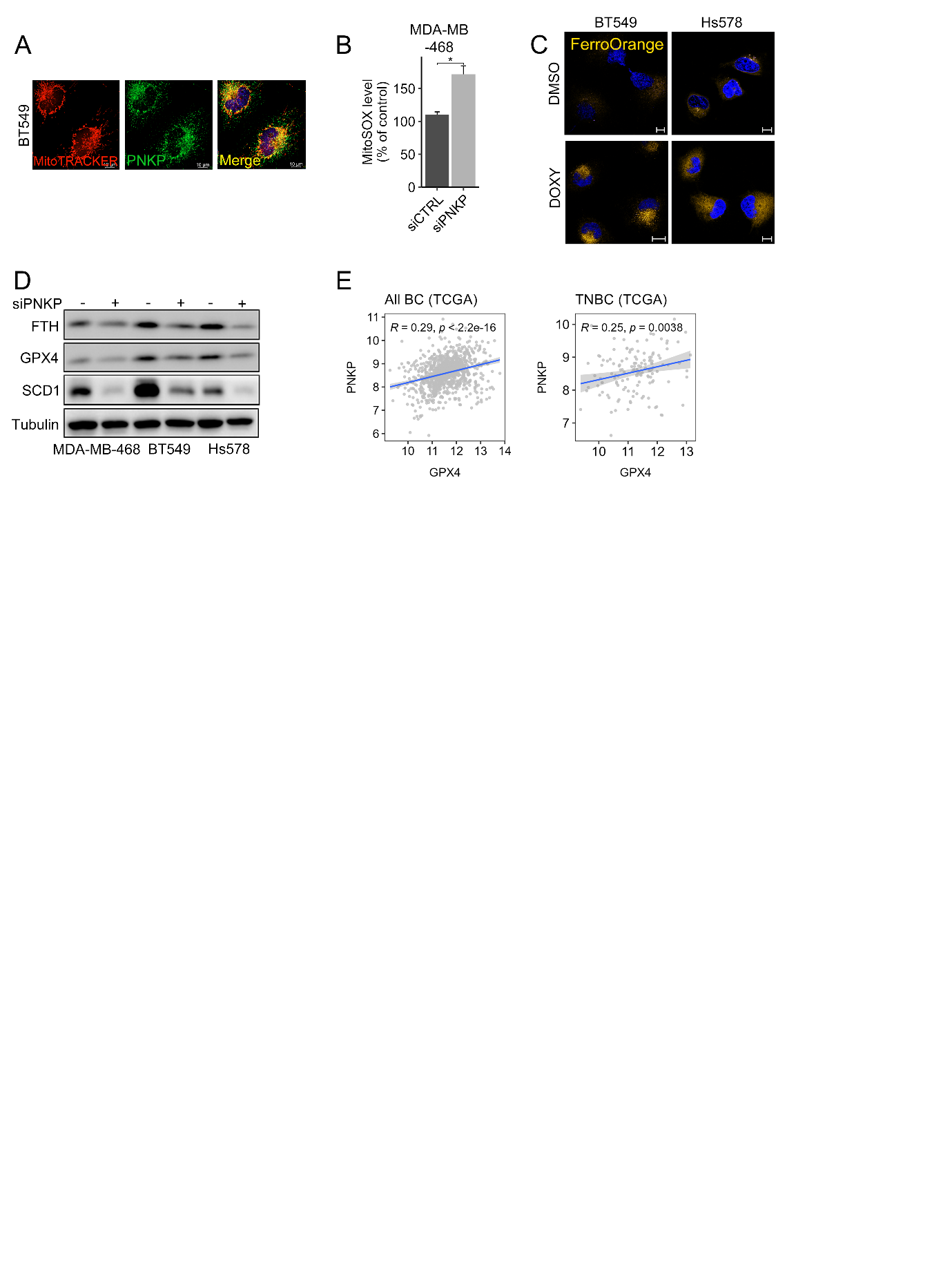


**Figure S2: Deletion of PNKP increases mitochondrial ROS, labile iron, and downregulates ferroptosis-associated proteins.**

**(A)** Localization of PNKP within the mitochondria. Shown are representative confocal images of BT549 cells co-stained with MitoTracker (red) and anti-PNKP antibody (green). Scale bar, 5 µm. **(B)** MDA-MB-468 cells transfected with the indicated siRNA were treated with MitoSOX and then subjected to flow cytometry analysis. Shown are the mean percentage values relative to siCTRL ± SD from two independent experiments. **(C)** The indicated TNBC cell lines expressing the inducible Tet-shPNKP were treated with DMSO (control) or doxycycline (DOXY, 1 µg/ml) for 96 hr, and then stained with FerroOrange. Representative confocal images are shown. Scale bar, 10 µm. **(D)** Cells transfected with siCTRL (-) or siPNKP (+) were assessed 72 hr after by WB analysis with anti-FTH, anti-GPX4 and anti-SCD1 antibodies. Tubulin was used as loading control. **(E)** Correlation between PNKP and GPX4 expression in breast cancer patients, data from TCGA.


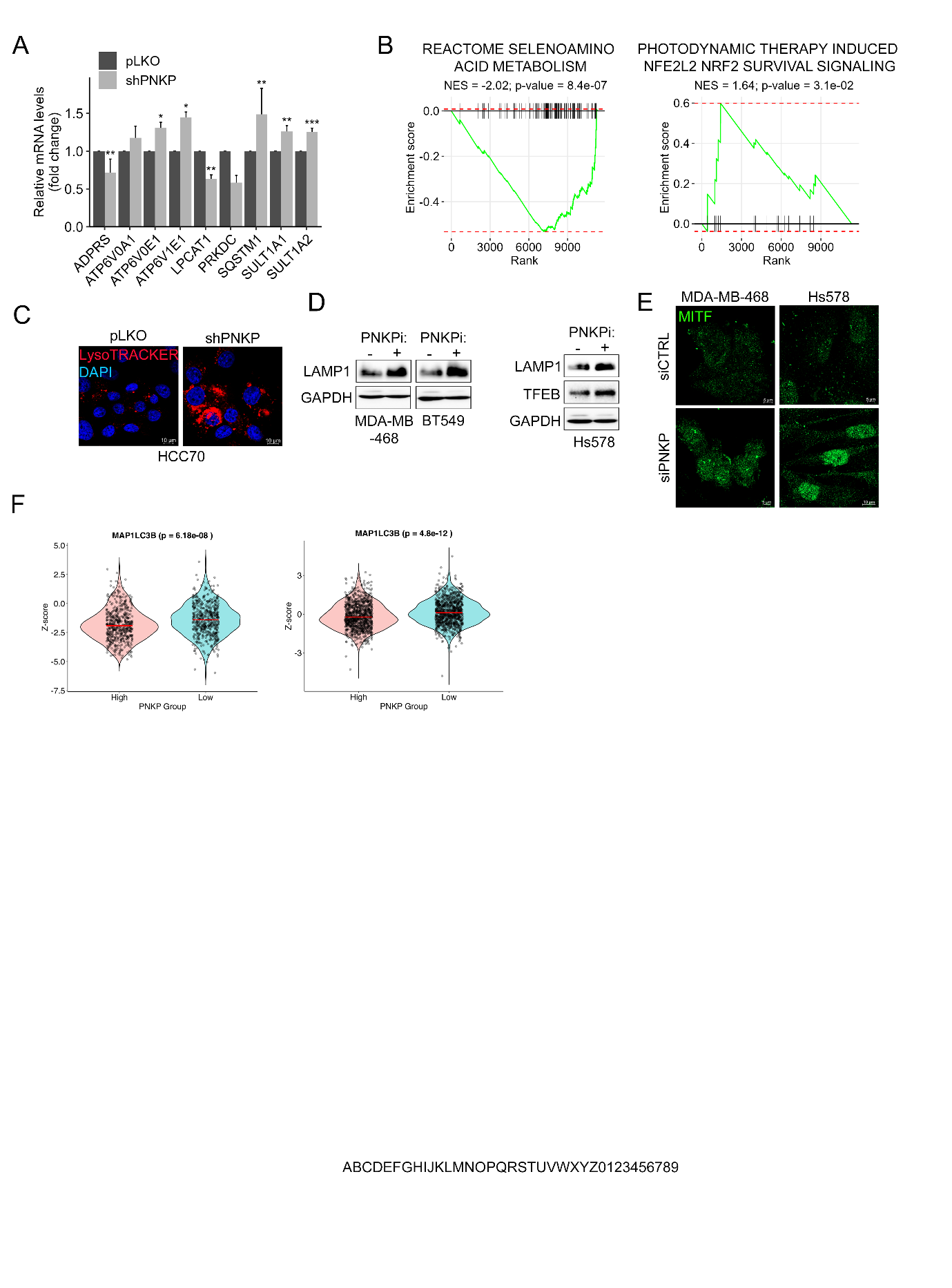


**Figure S4: Increased DNA damage, lysosomal activity, lipid-ROS and labile iron in PNKP depleted cells is STING dependent.**

**(A)** TNBC cells treated with PNKP inhibitor for the indicated time points and assessed by WB using pγH2AX antibody. GAPDH was used for loading control. **(B)** Increased cytoplasmic dsDNA in PNKP KD (siPNKP) cells is shown by the reduced co-localization of MitoTracker (red) with dsDNA antibody (green) as determined by IF analysis. Square borders indicate zoom areas. Scale bar, 5 µm. **(C-H)** Depletion of STING by shRNA suppressed the ferroptosis-associated phenotype of PNKP depleted cells. The indicated cells expressing either pLKO (empty virus) or shSTING1 transfected with siCTRL or siPNKP, and 72 hr later assessed for STING knockdown by qPCR (representative graph) (C), stained with LysoTracker (D), BODIPY-C11 (E-F), or FerroOrange (G-H), and processed for confocal microscopy (scale bar 20 µM D, E). Fluorometric quantifications (E-G) were calculated relative to control (siCTRL) and the mean values ± SD from 3 experiments are shown.

**
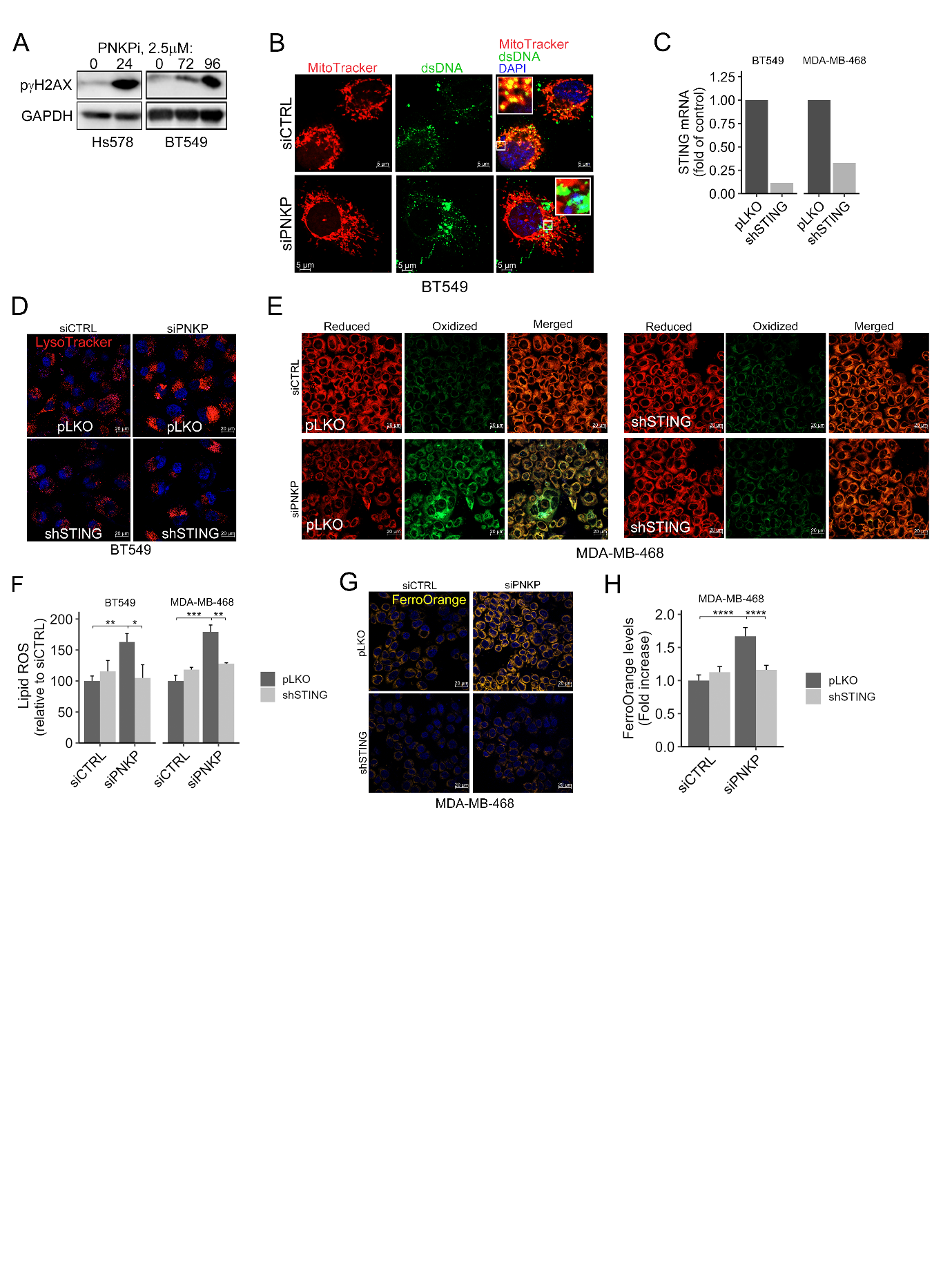
**

**Figure S4: PNKP depletion increases DNA damage and cytoplasmic dsDNA fragments.**

**(A)** TNBC cells treated with PNKP inhibitor for the indicated time points and assessed for the DNA damage by WB using pγH2AX antibody. GAPDH was used for loading control. **(B)** Increased cytoplasmic dsDNA in PNKP KD (siPNKP) is shown by the increased signal of dsDNA and the reduced co-localization between MitoTracker with dsDNA as determined by IF analysis. Square borders indicate zoom areas. Scale bar, 5 µm. **(C-H)** Depletion of STING by shRNA suppressed the ferroptosis-associated phenotype of PNKP depleted cells. The indicated cells expressing either pLKO (empty virus) or shSTING1 were transfected with siCTRL or siPNKP, and 72 hr later assessed for STING knockdown by qPCR (representative graph) (C), stained with LysoTracker (D), BODIPY-C11 (E-F), or FerroOrange (G-H), and processed for confocal microscopy (scale bar 20 µM). Fluorometric quantifications (E-G) were calculated relative to control (siCTRL) and the mean values ± SD from 3 experiments is shown.

**
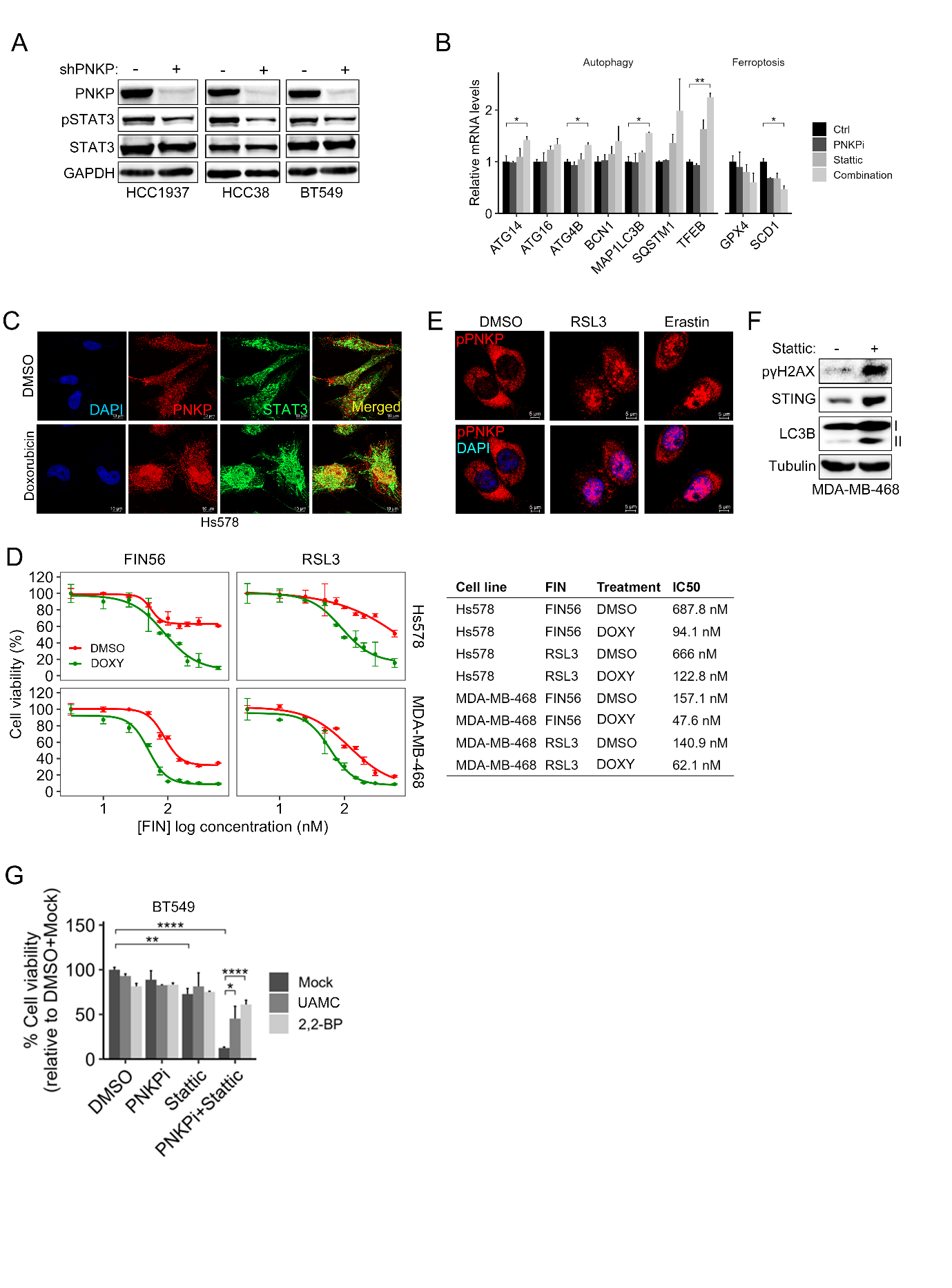
**

**Figure S5: PNKP perturbation modulates STAT3 activity.**

**(A)** Control (-) or PNKP-depleted (shPNKP) (+) TNBC cells were analyzed by WB for the indicated proteins. STAT3 phosphorylation (pSTAT3) was assessed by anti-pSTAT3(Y705) antibody. GAPDH was used for loading control. **(B)** Inhibition of PNKP (PNKPi; 10 µM) and/or STAT3 (Stattic, 2 µM) decreased the levels of ferroptosis associated genes and increased autophagic markers in MDA-MB-468 cells as determined by qPCR analysis. Shown is a representative experiment out of two repeats. **(C)** The nuclear colocalization of PNKP (red) and STAT3 (green) is enhanced following doxorubicin treatment (0.1 µM, 24 hr) in Hs578 cells. Scale bar, 10 µm. **(D)** Dose response curve of FIN56 (left panel) and RSL3 (right panel) in the indicated cell lines expressing the Tet-shPNKP treated with DMSO (red line) or doxycycline, 1µg/ml (DOXY) (green line) for 72 hr and then assessed for cell viability by MTT. The mean percentage relative to control (DMSO) ± SD from two independent experiments is shown. The table shows the IC_50_ values for the FINs in different TNBC cell lines depleted for PNKP (DOXY) compared to control DMSO. **(E)** Enhanced nuclear localization of pPNKP (S114/T118) following RSL3 (0.2 µM, 4 hrs) or Erastin (2µM,10 hrs) treatments in MDA-MB-468 cells. Shown are representative confocal images. Scale bar, 5 µm. **(F)** STAT3 inhibition enhances pγH2AX, STING and LC3B levels. Cells were treated with Stattic (0.25 µM, 24 hr) and assessed by WB for the indicated markers. β-tubulin was used as loading control. **(G)** Co-inhibition of PNKP and STAT3 induced ferroptotic cell death. BT549 cells were treated with the indicated drugs in the absence or presence of iron chelator (2,2’BP, 8 µM) or UAMC-3203 (UAMC, 2.5 µM) and assessed 72 hr later for cell viability by MTT. Influence on cell viability is presented relative (%) to control untreated cells. Shown are mean values ± SD from three experiments.


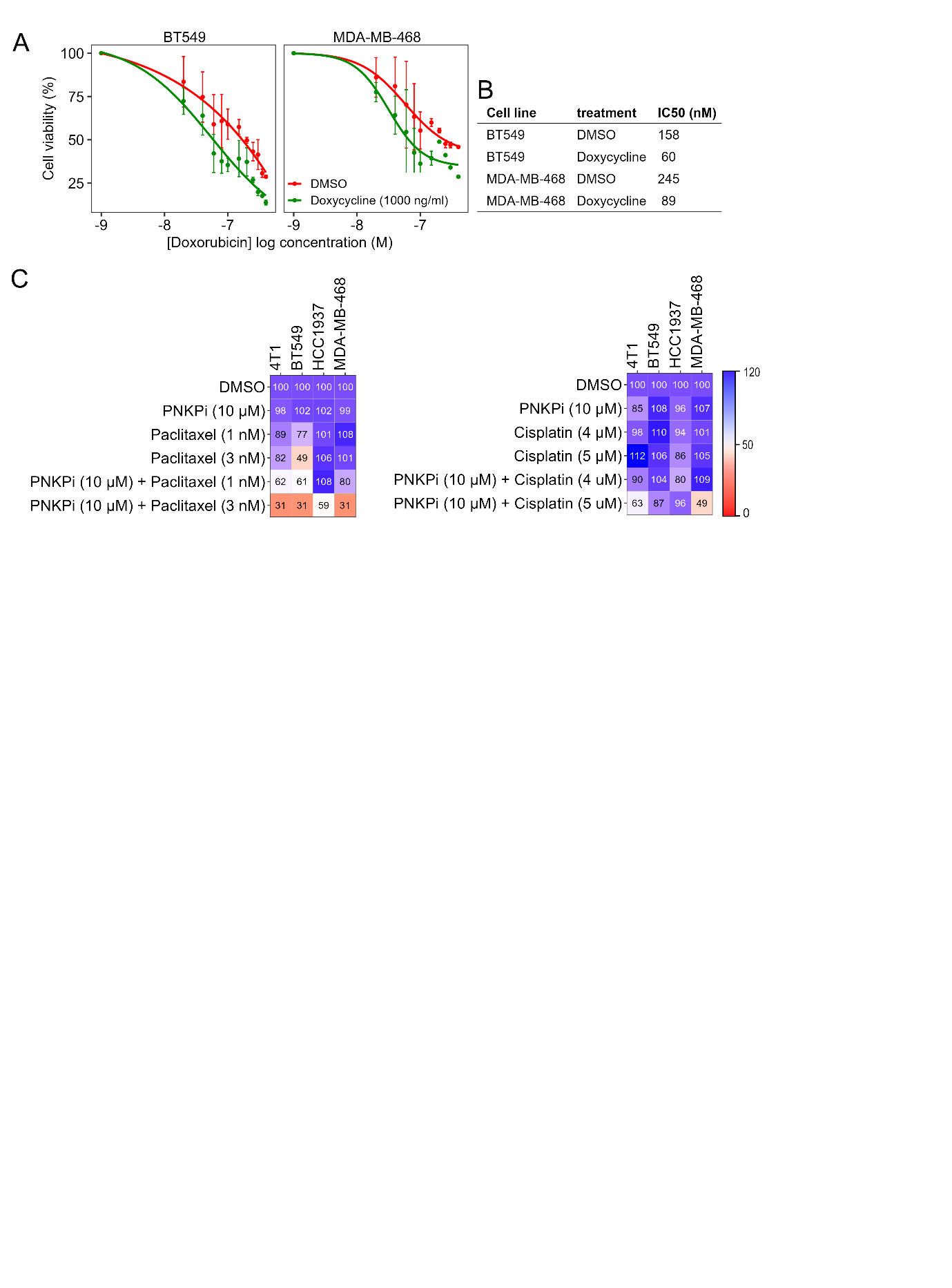


**Figure S6: Targeting of PNKP in combination with chemotherapy decreased cell viability of TNBC cells.**

**(A)** Dose-response curves of doxorubicin in the indicated cell lines (A) expressing the inducible Tet-shPNKP treated with DMSO (red line) or doxycycline, 1µg/ml (green line) for 72 hr and then assessed by MTT for cell viability. The mean percentage relative to control (DMSO) ± SD from 2-6 repeats is shown. **(B)** Table showing the IC_50_ values for doxorubicin in the above-described cells depleted of PNKP (doxycycline) and control DMSO. **(C)** Heatmap representations of single drug and drugs combinations applied in several TNBC cell lines. Cell viability was measured 72 hr later by MTT and is shown as percentage of control (DMSO).
