# Supplementary material for: PNKP targeting engages the autophagic machinery through STING and STAT3 to potentiate ferroptosis and chemotherapy in TNBC
Source: Redox Biol. 2025 Jul 22;86:103775. doi: 10.1016/j.redox.2025.103775 (PMC12336819; doi:10.1016/j.redox.2025.103775)
Supplement: Multimedia component 3 [file mmc3.pptx]

## Slide 1
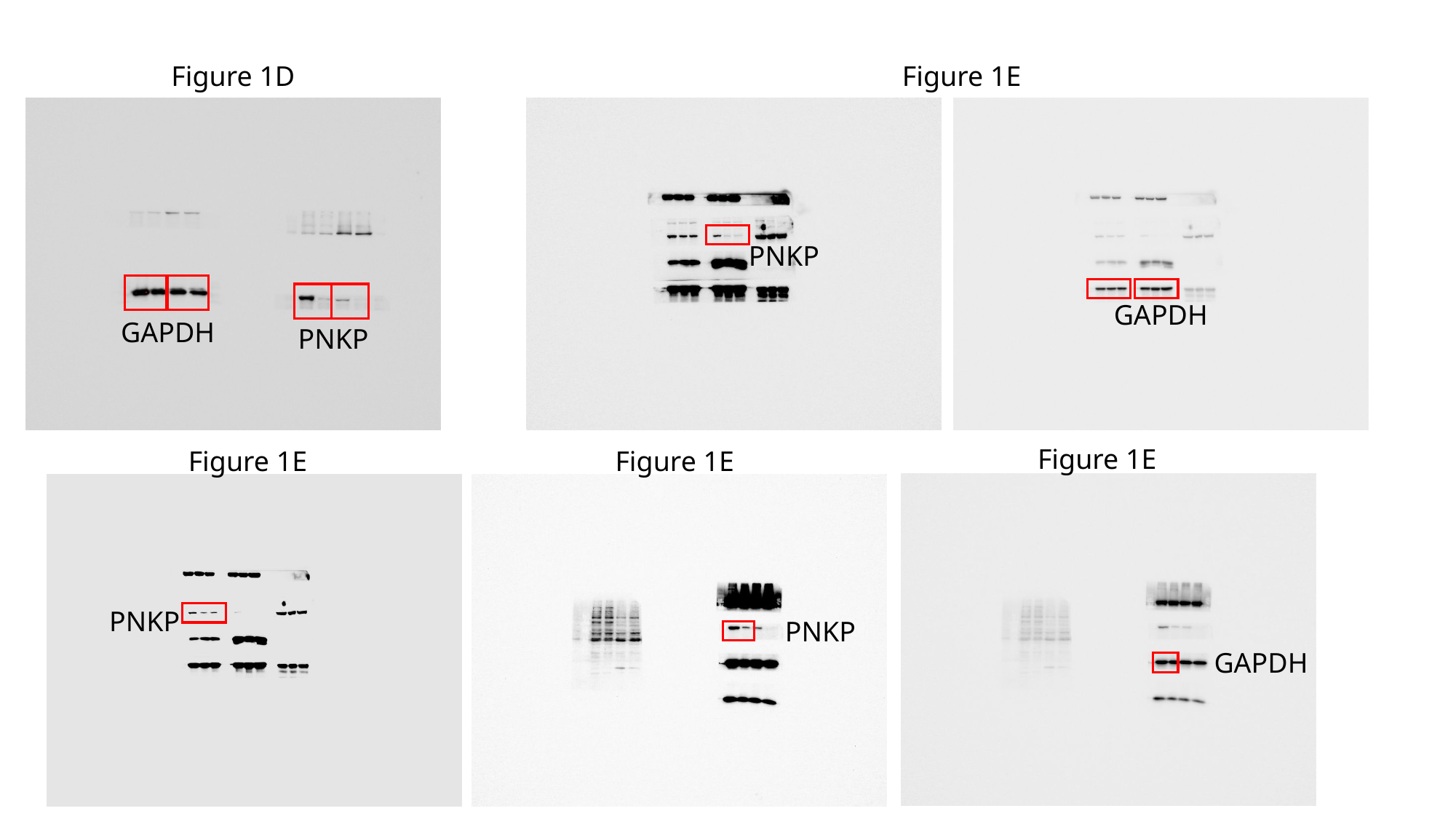

Figure 1E
Figure 1D
PNKP
GAPDH
GAPDH
PNKP
Figure 1E
Figure 1E
Figure 1E
PNKP
PNKP
GAPDH

## Slide 2
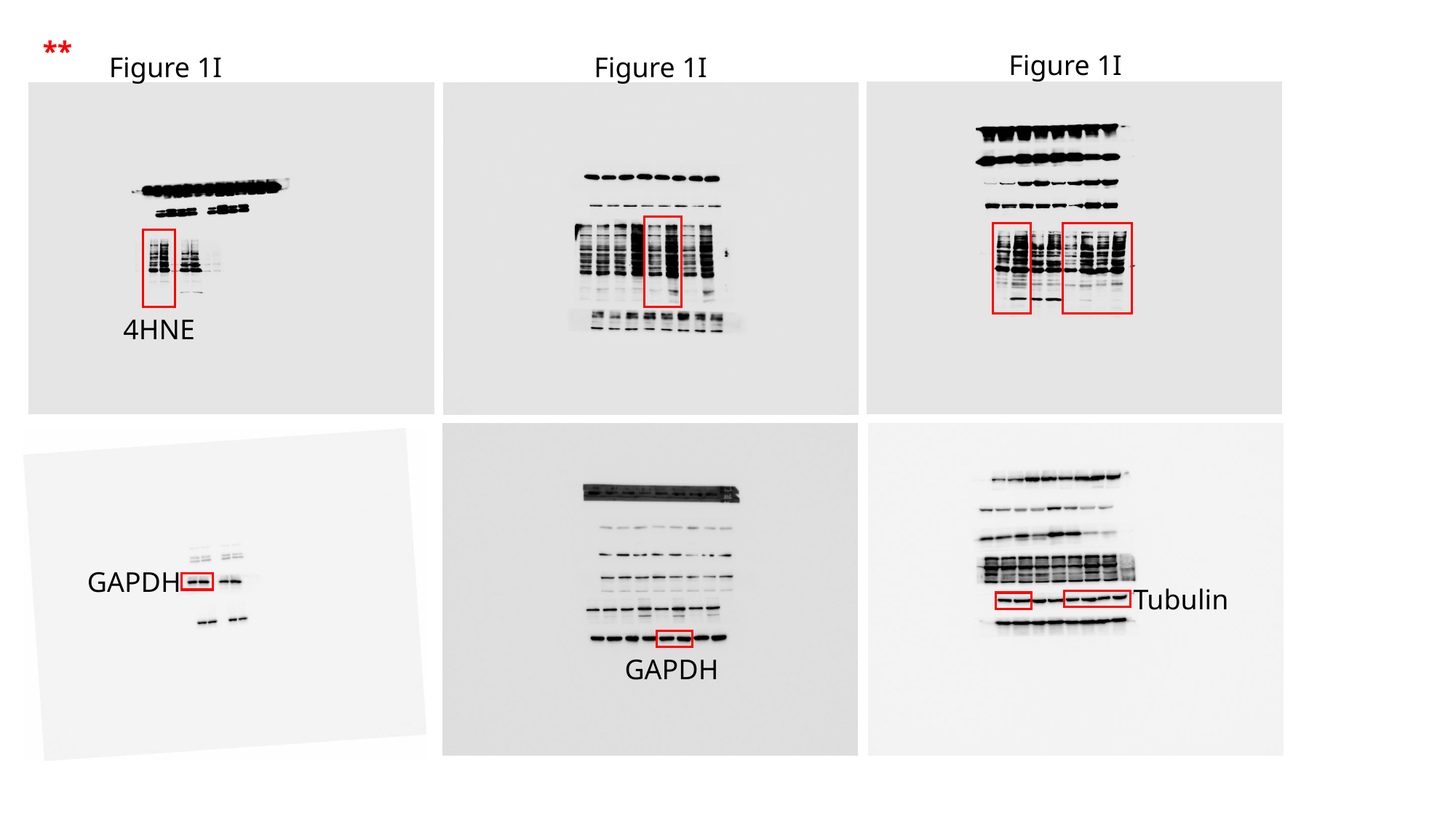

**
Figure 1I
Figure 1I
Figure 1I
4HNE
GAPDH
Tubulin
GAPDH

## Slide 3
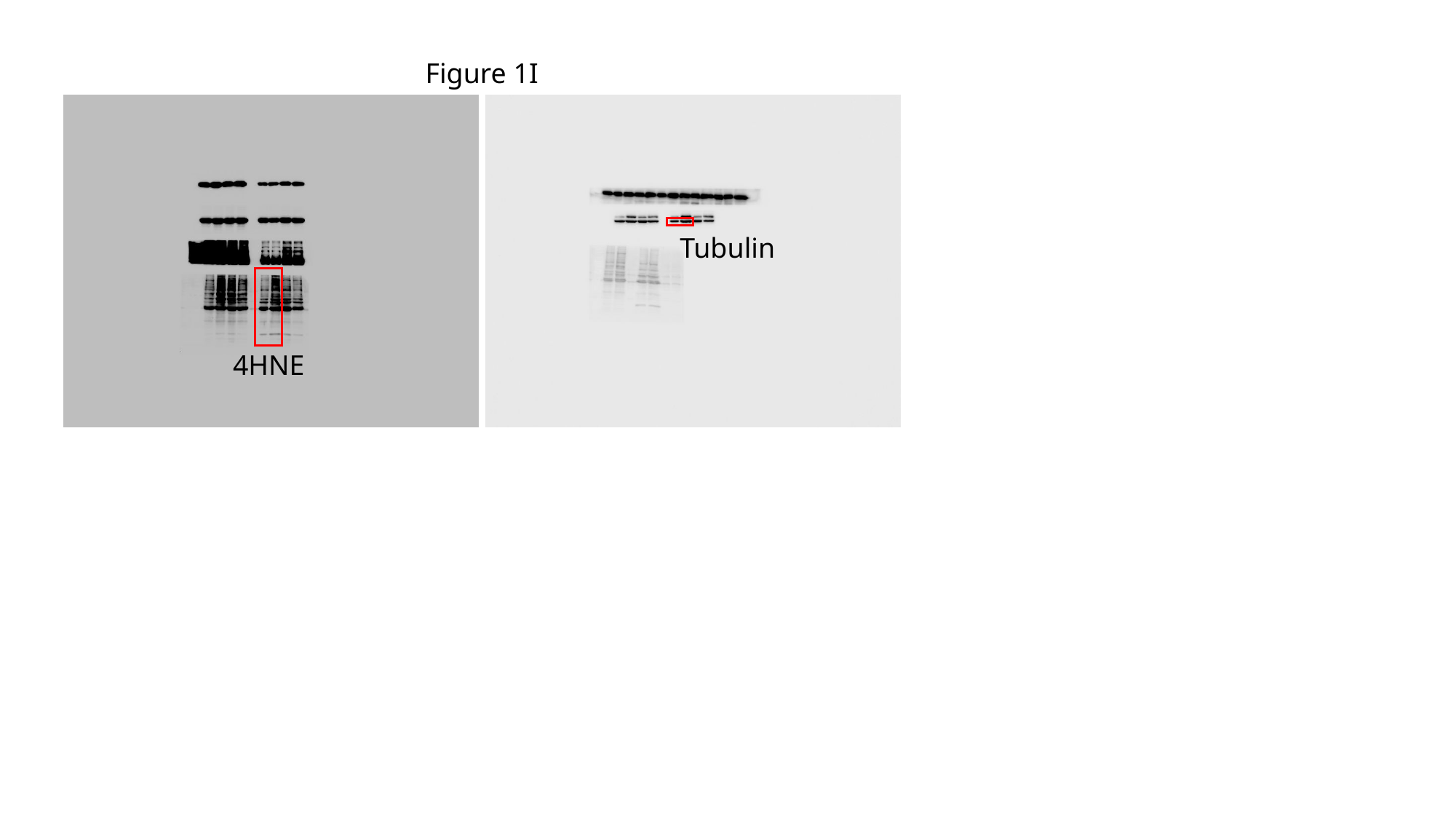

Figure 1I
Tubulin
4HNE

## Slide 4
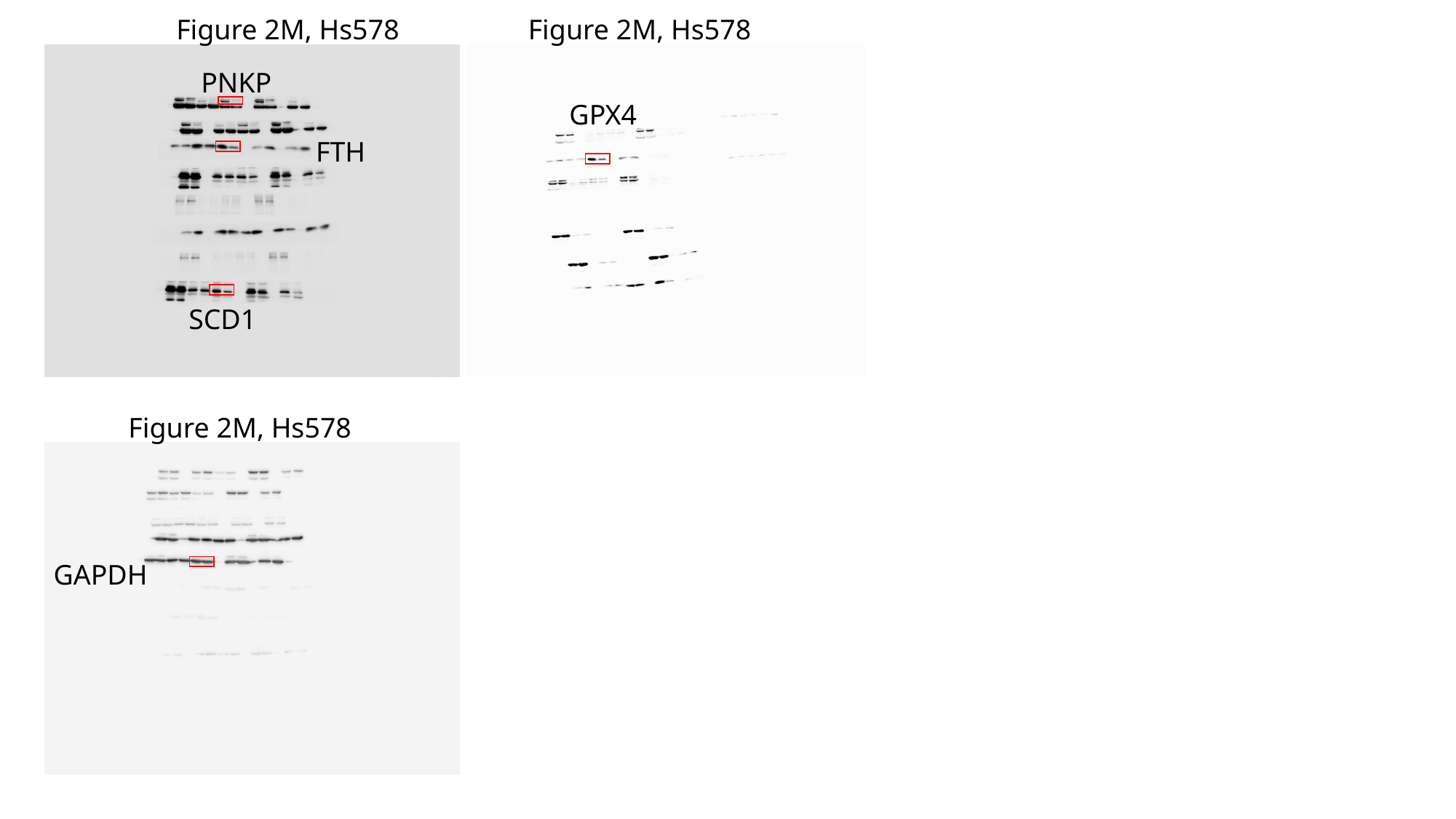

Figure 2M, Hs578
Figure 2M, Hs578
PNKP
GPX4
FTH
SCD1
Figure 2M, Hs578
GAPDH

## Slide 5
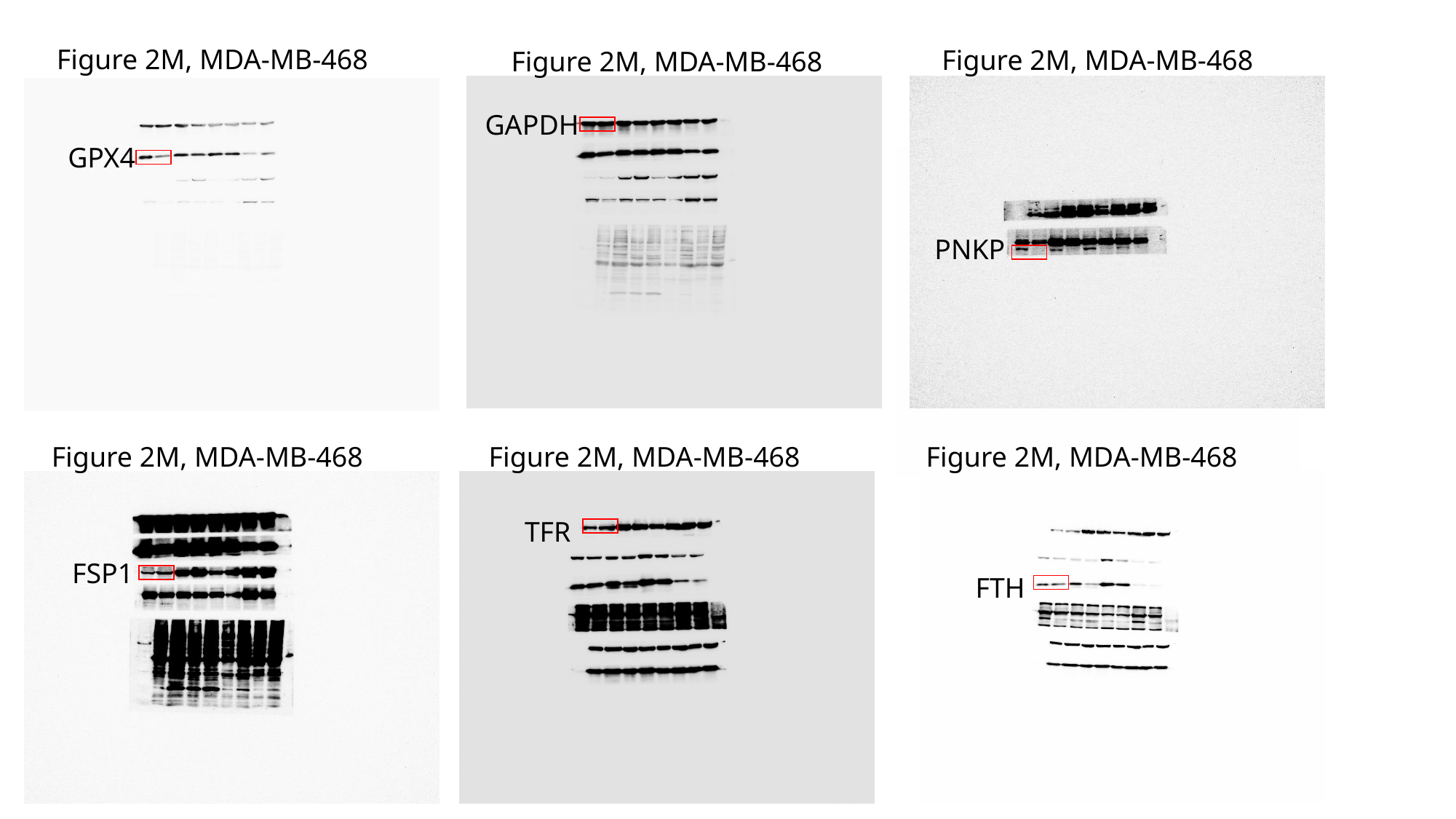

Figure 2M, MDA-MB-468
Figure 2M, MDA-MB-468
Figure 2M, MDA-MB-468
GAPDH
GPX4
PNKP
Figure 2M, MDA-MB-468
Figure 2M, MDA-MB-468
Figure 2M, MDA-MB-468
TFR
FSP1
FTH

## Slide 6
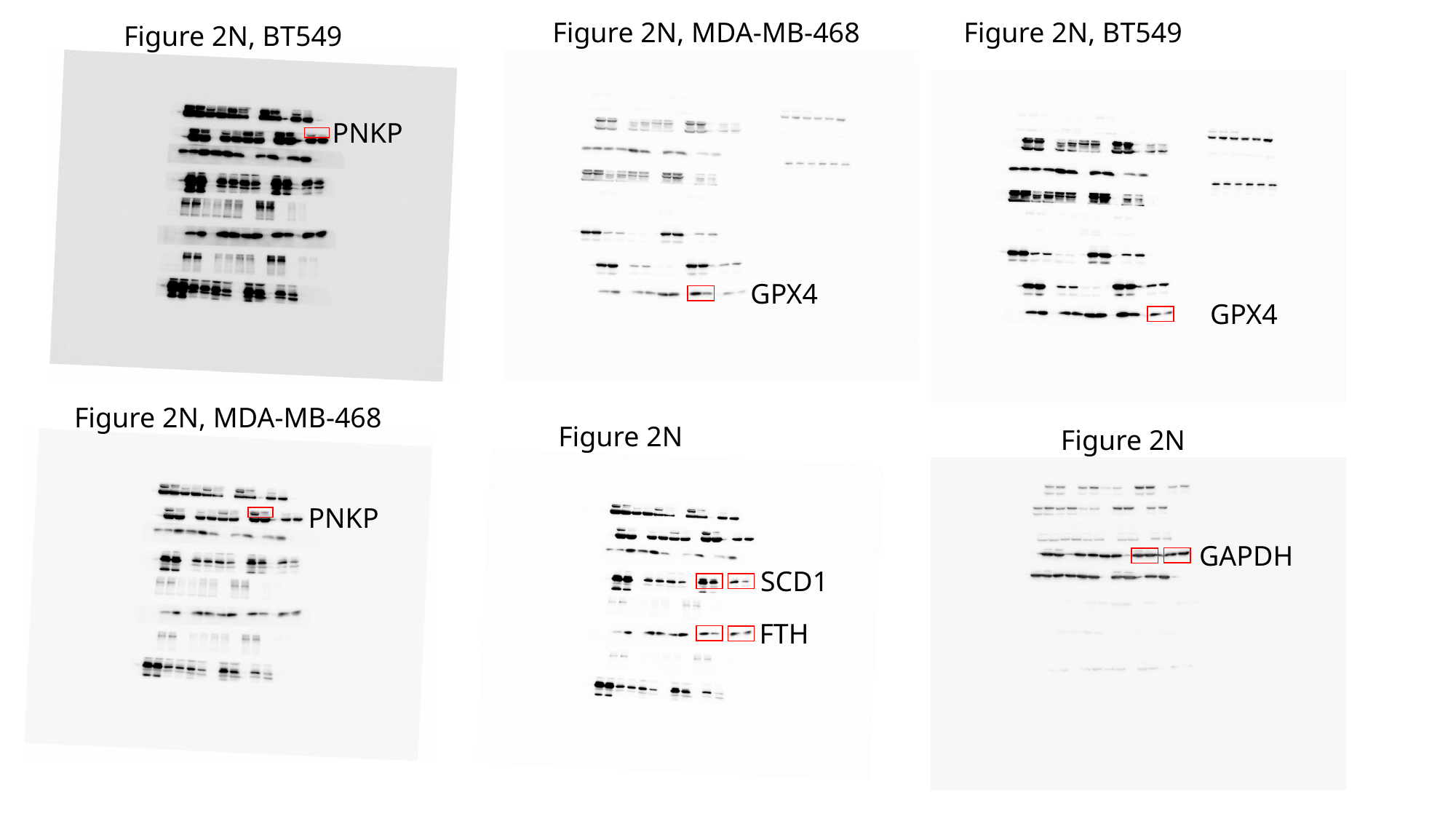

Figure 2N, MDA-MB-468
Figure 2N, BT549
Figure 2N, BT549
PNKP
GPX4
GPX4
Figure 2N, MDA-MB-468
Figure 2N
Figure 2N
PNKP
GAPDH
SCD1
FTH

## Slide 7
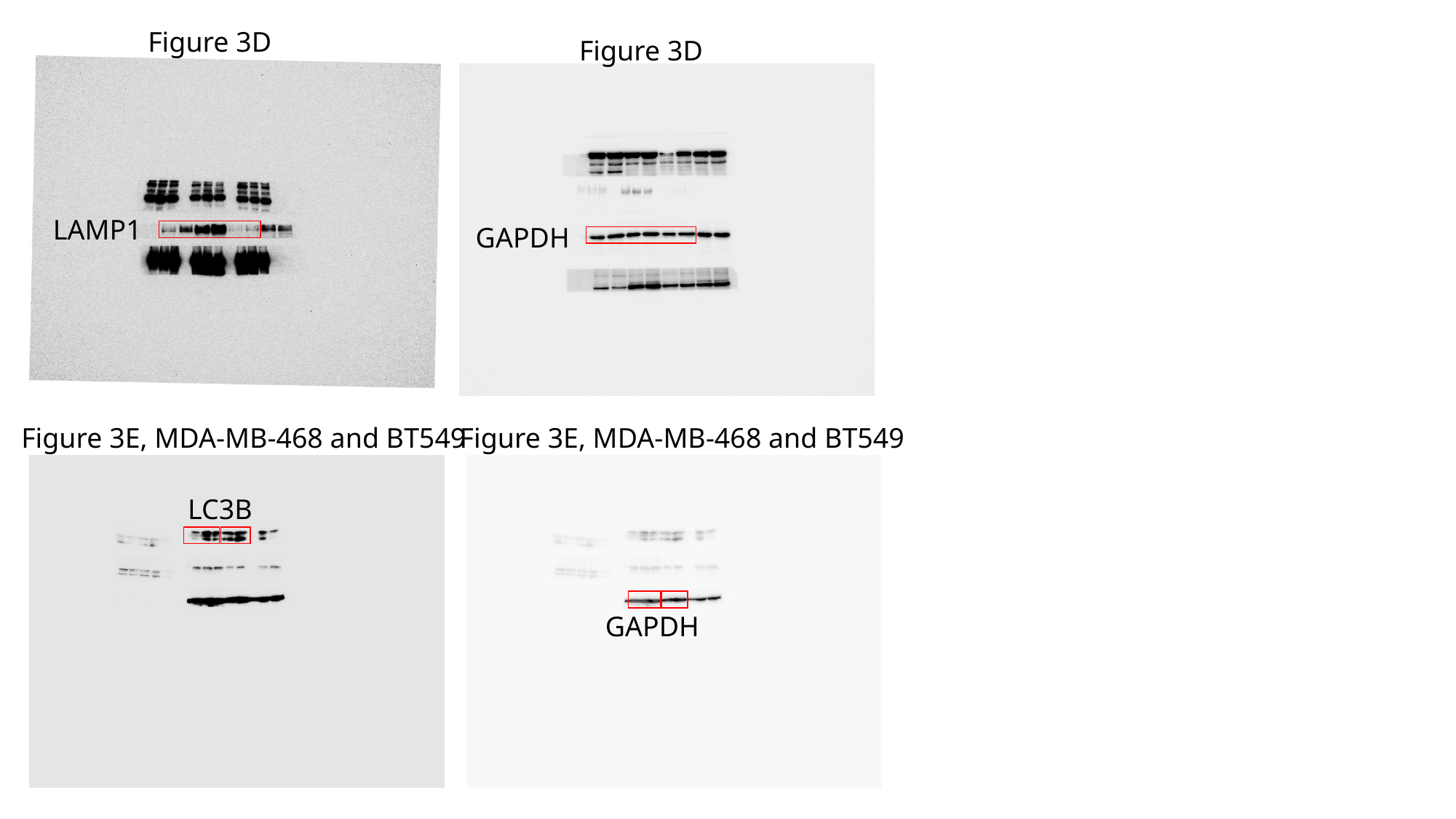

Figure 3D
Figure 3D
LAMP1
GAPDH
Figure 3E, MDA-MB-468 and BT549
Figure 3E, MDA-MB-468 and BT549
LC3B
GAPDH

## Slide 8
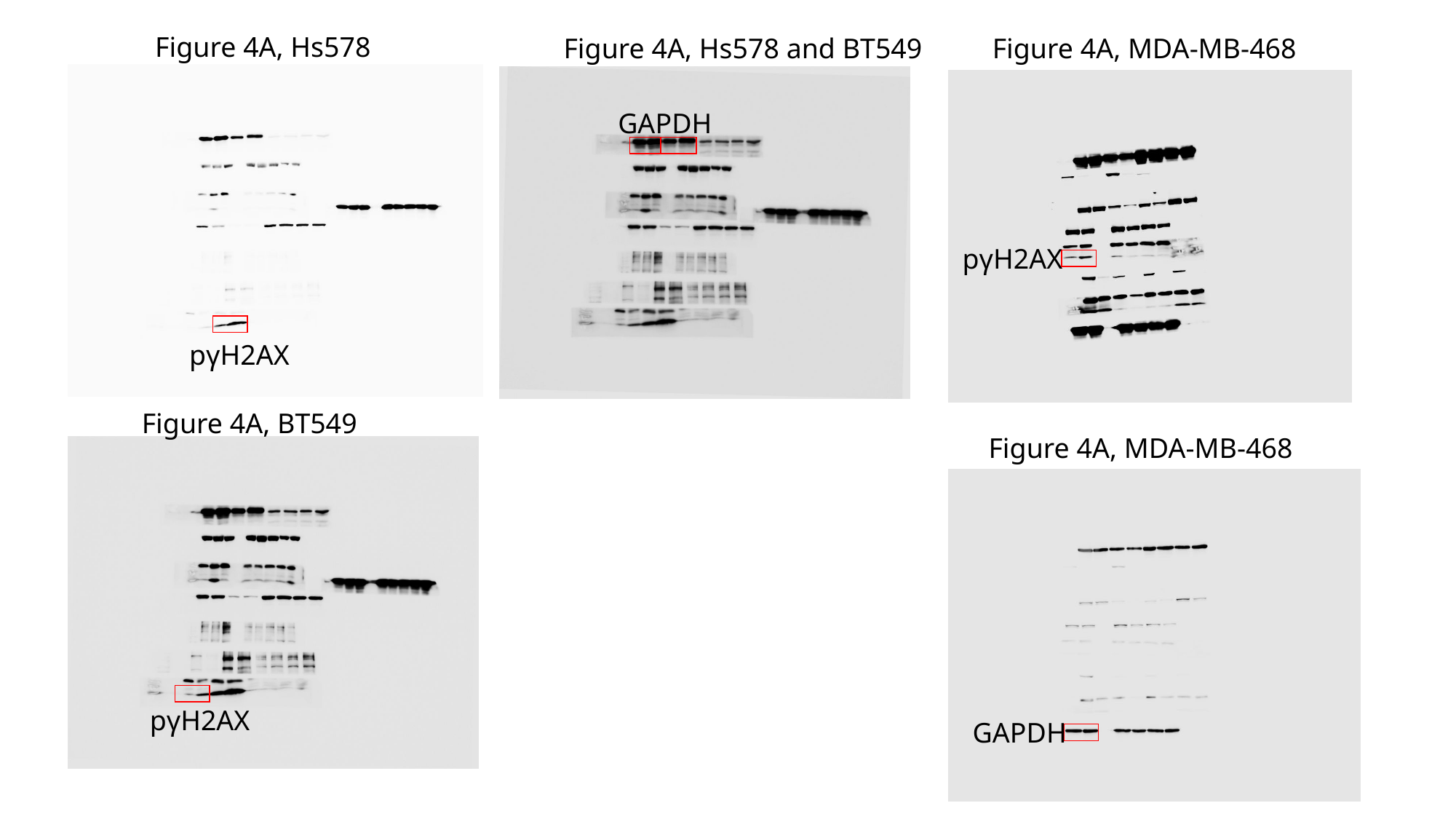

Figure 4A, Hs578
Figure 4A, Hs578 and BT549
Figure 4A, MDA-MB-468
GAPDH
pγH2AX
pγH2AX
Figure 4A, BT549
Figure 4A, MDA-MB-468
pγH2AX
GAPDH

## Slide 9
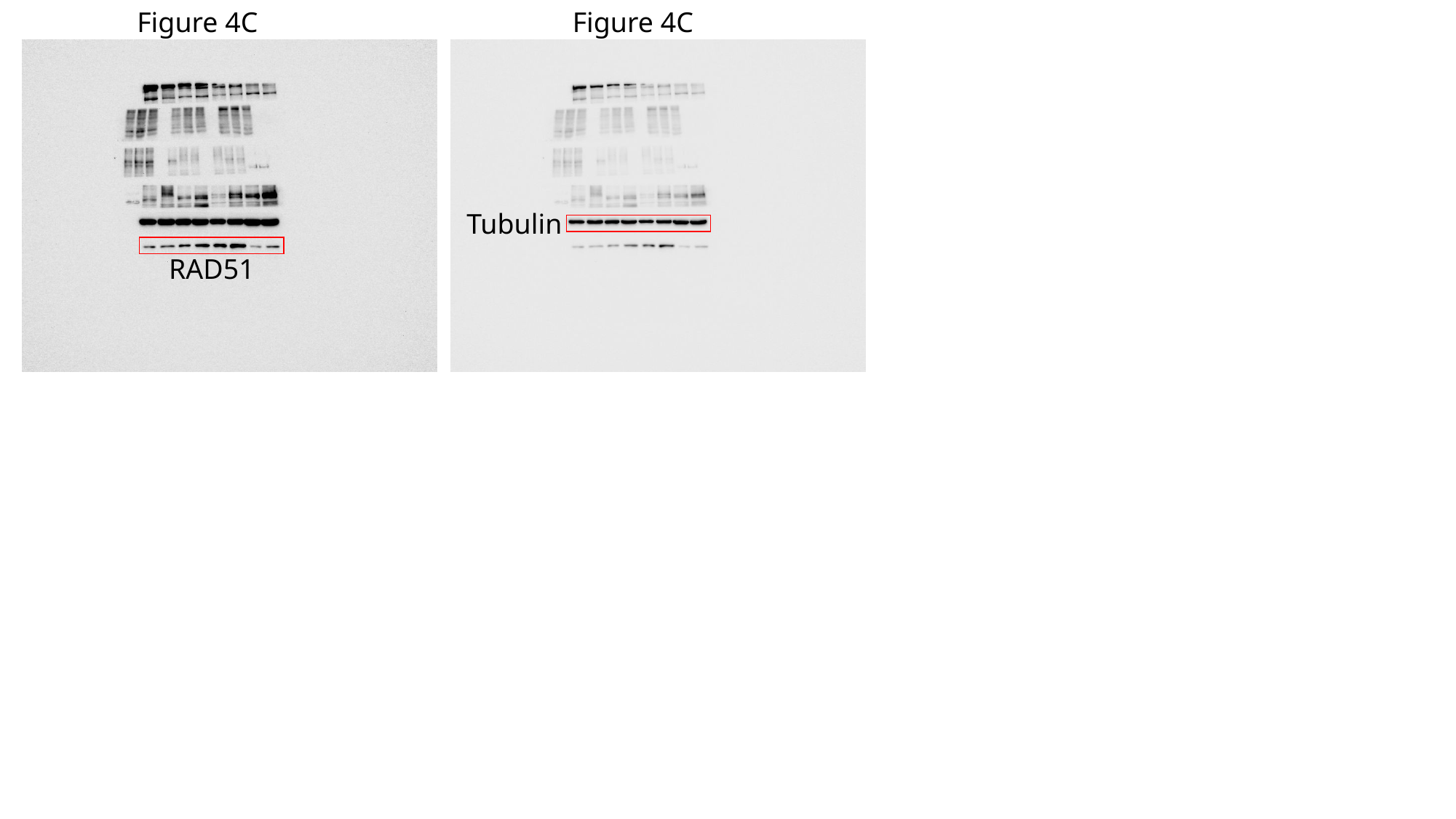

Figure 4C
Figure 4C
Tubulin
RAD51

## Slide 10
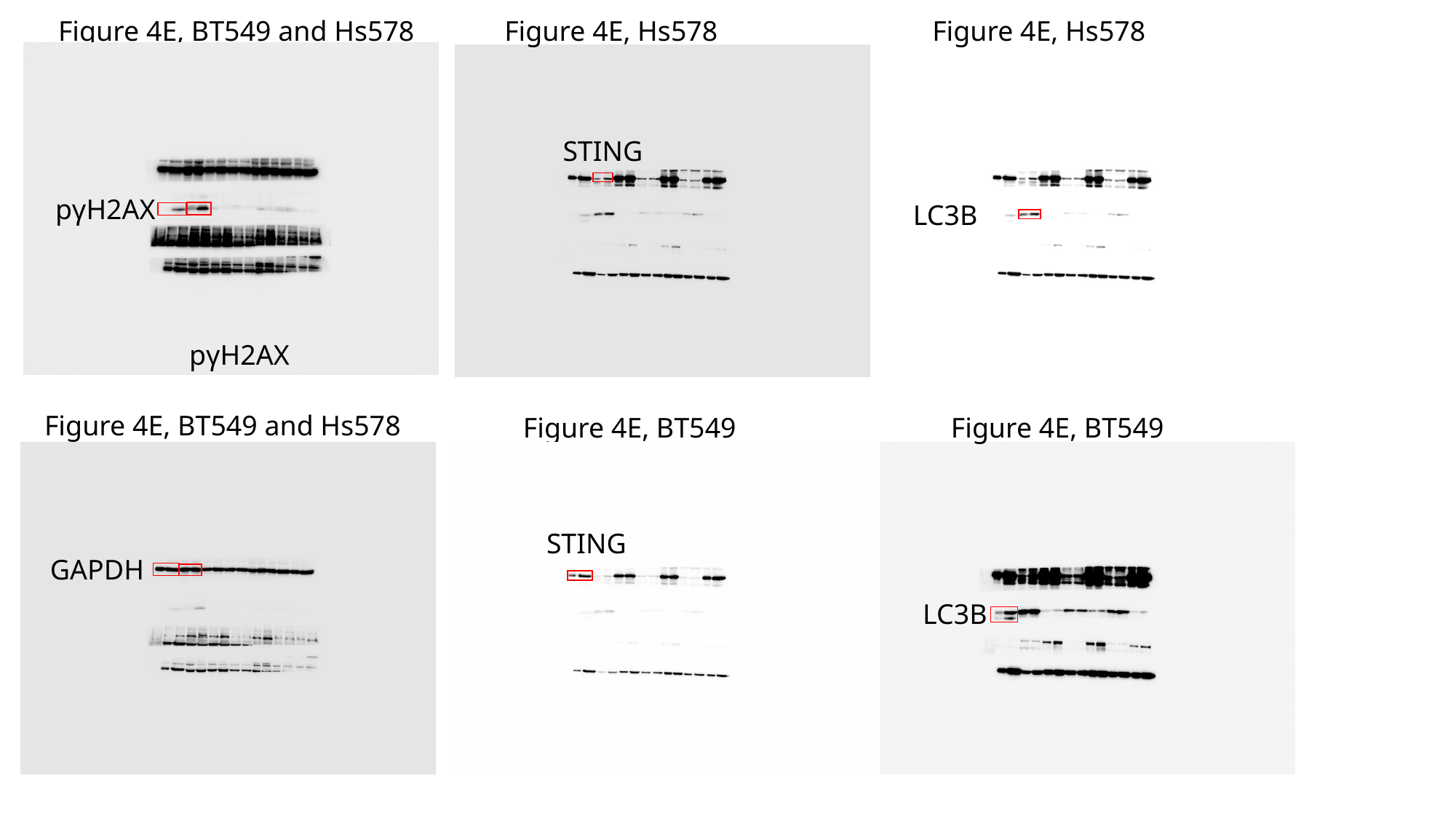

Figure 4E, BT549 and Hs578
Figure 4E, Hs578
Figure 4E, Hs578
STING
pγH2AX
LC3B
pγH2AX
Figure 4E, BT549 and Hs578
Figure 4E, BT549
Figure 4E, BT549
STING
GAPDH
LC3B

## Slide 11
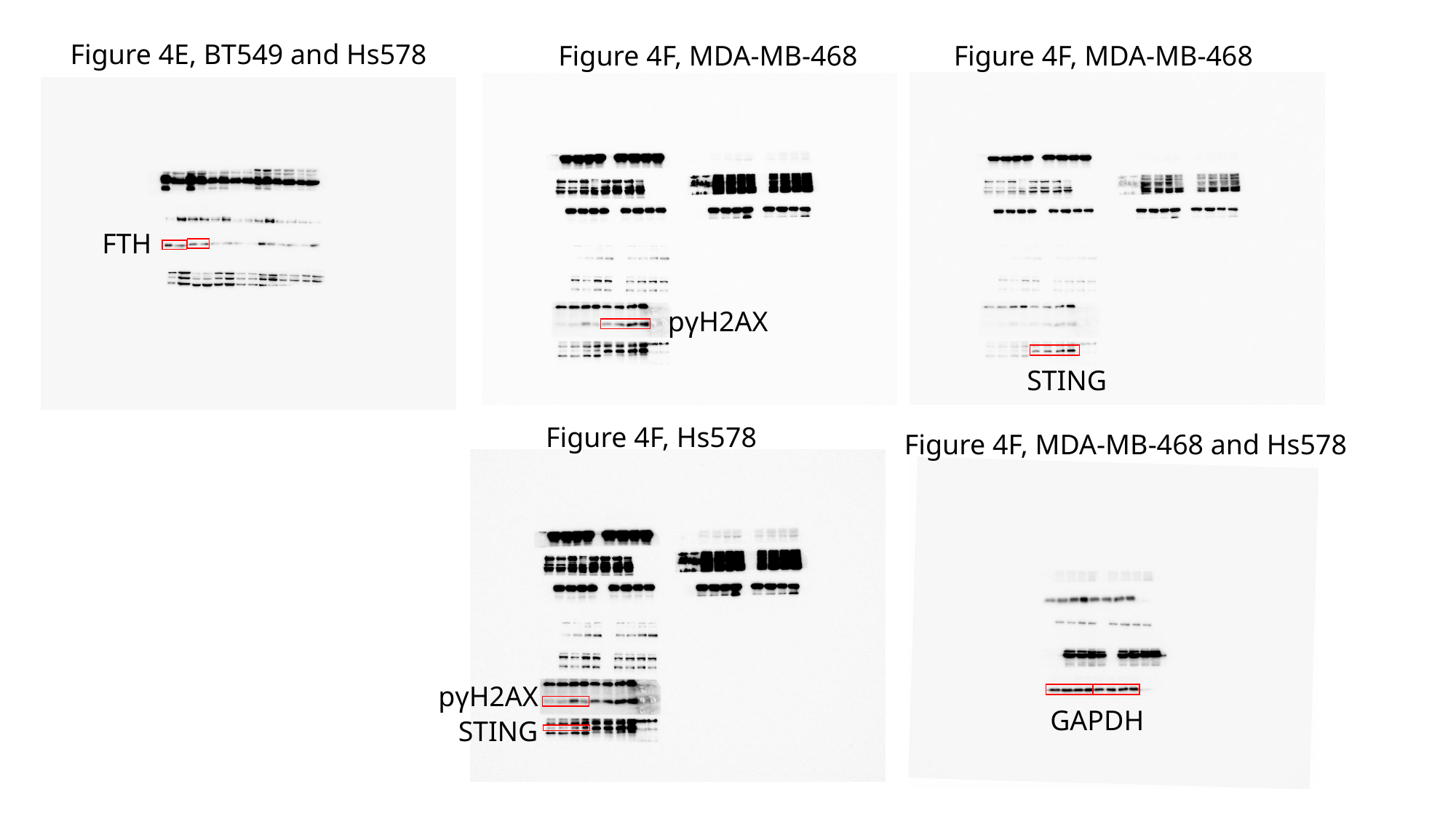

Figure 4E, BT549 and Hs578
Figure 4F, MDA-MB-468
Figure 4F, MDA-MB-468
FTH
pγH2AX
STING
Figure 4F, Hs578
Figure 4F, MDA-MB-468 and Hs578
pγH2AX
GAPDH
STING

## Slide 12
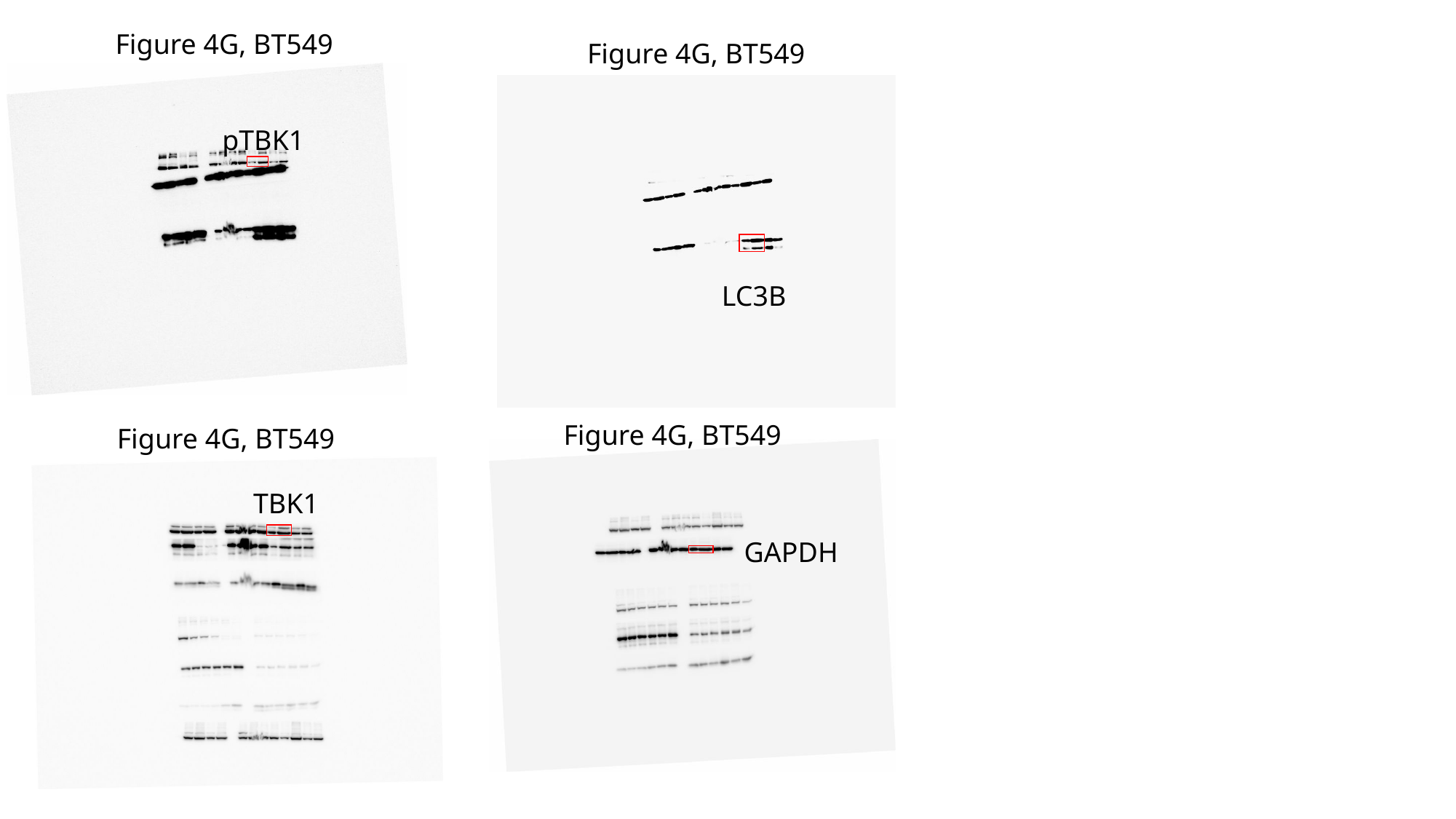

Figure 4G, BT549
Figure 4G, BT549
pTBK1
LC3B
Figure 4G, BT549
Figure 4G, BT549
TBK1
GAPDH

## Slide 13
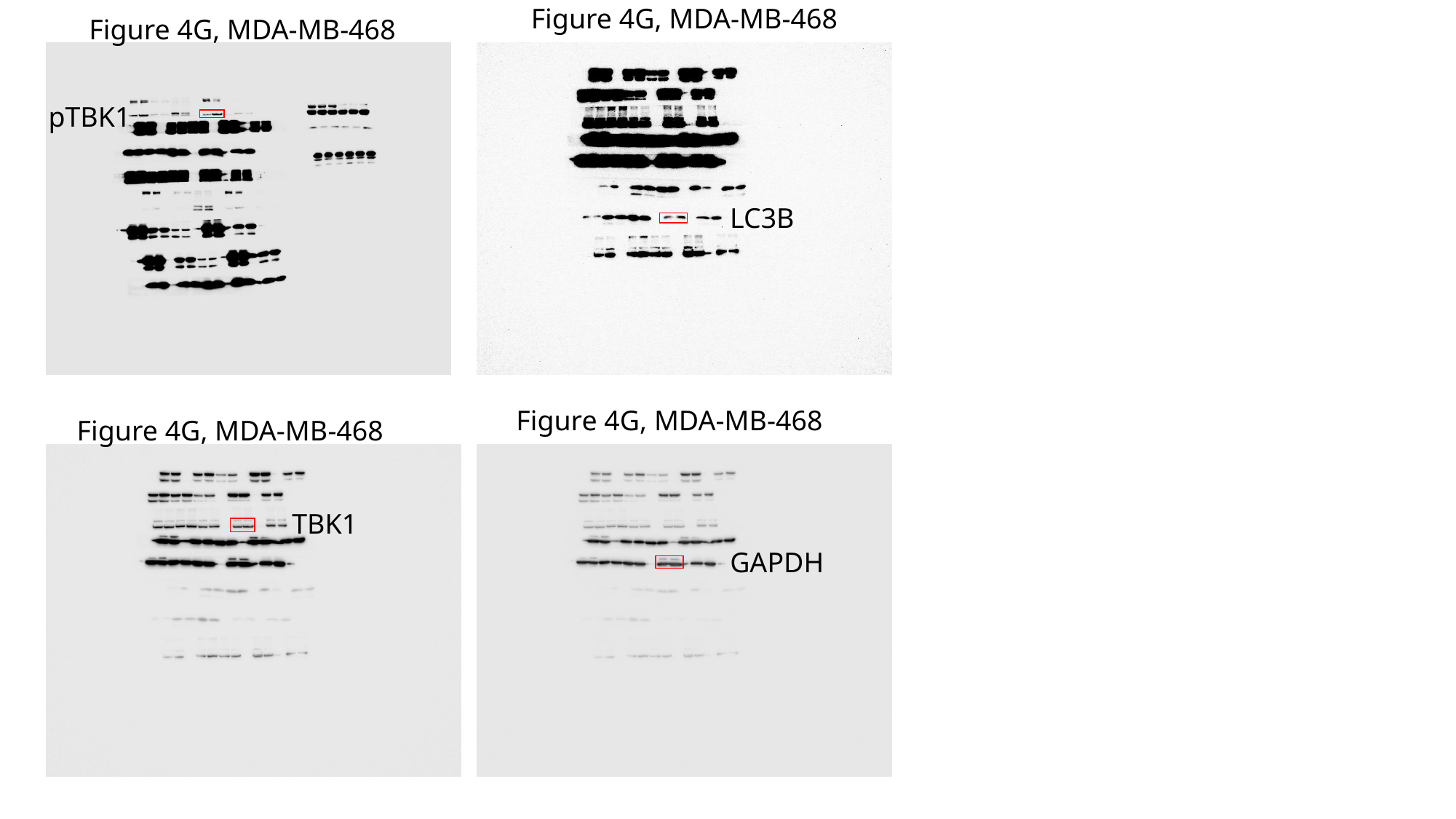

Figure 4G, MDA-MB-468
Figure 4G, MDA-MB-468
pTBK1
LC3B
Figure 4G, MDA-MB-468
Figure 4G, MDA-MB-468
TBK1
GAPDH

## Slide 14
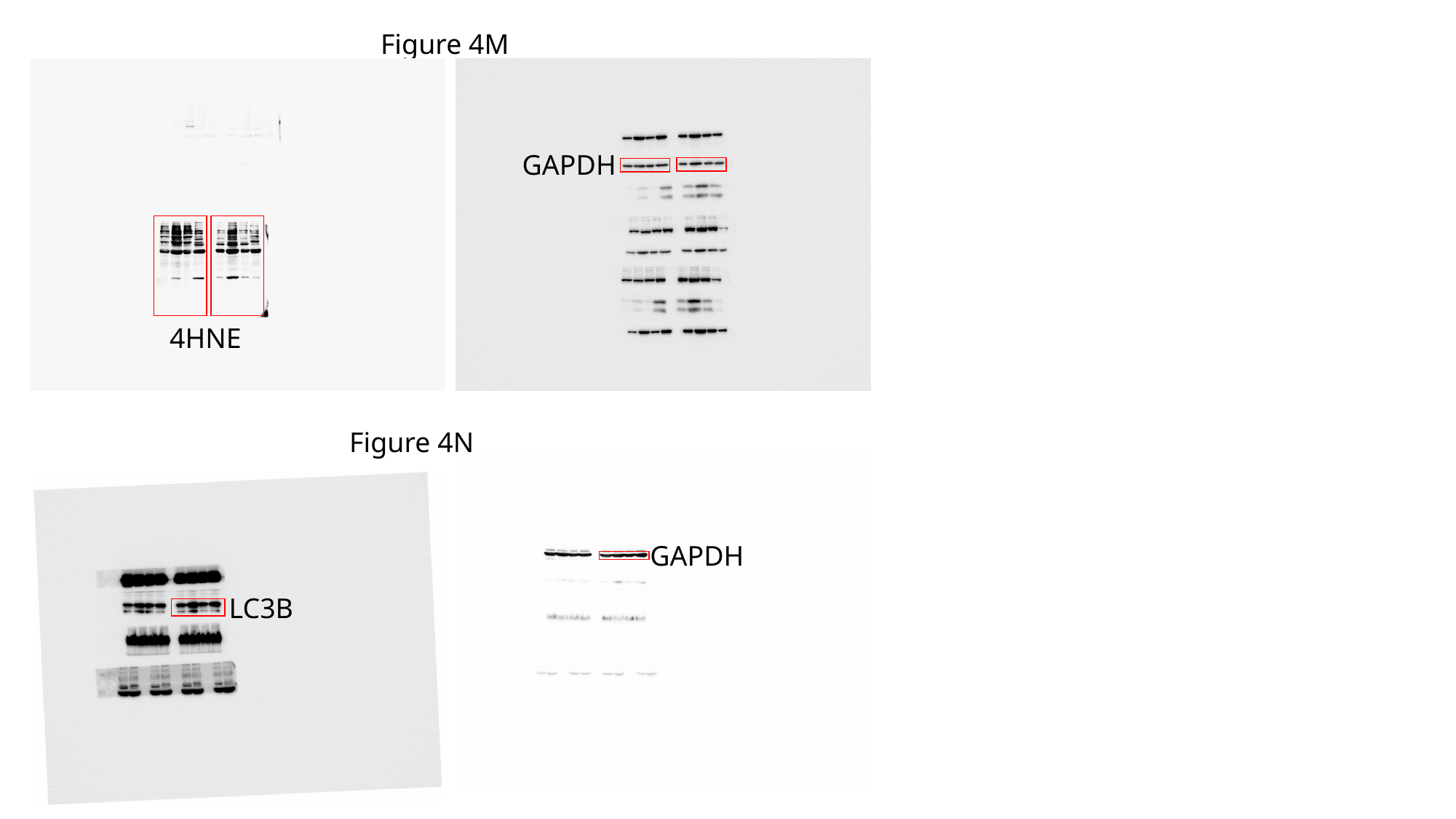

Figure 4M
GAPDH
4HNE
Figure 4N
GAPDH
LC3B

## Slide 15
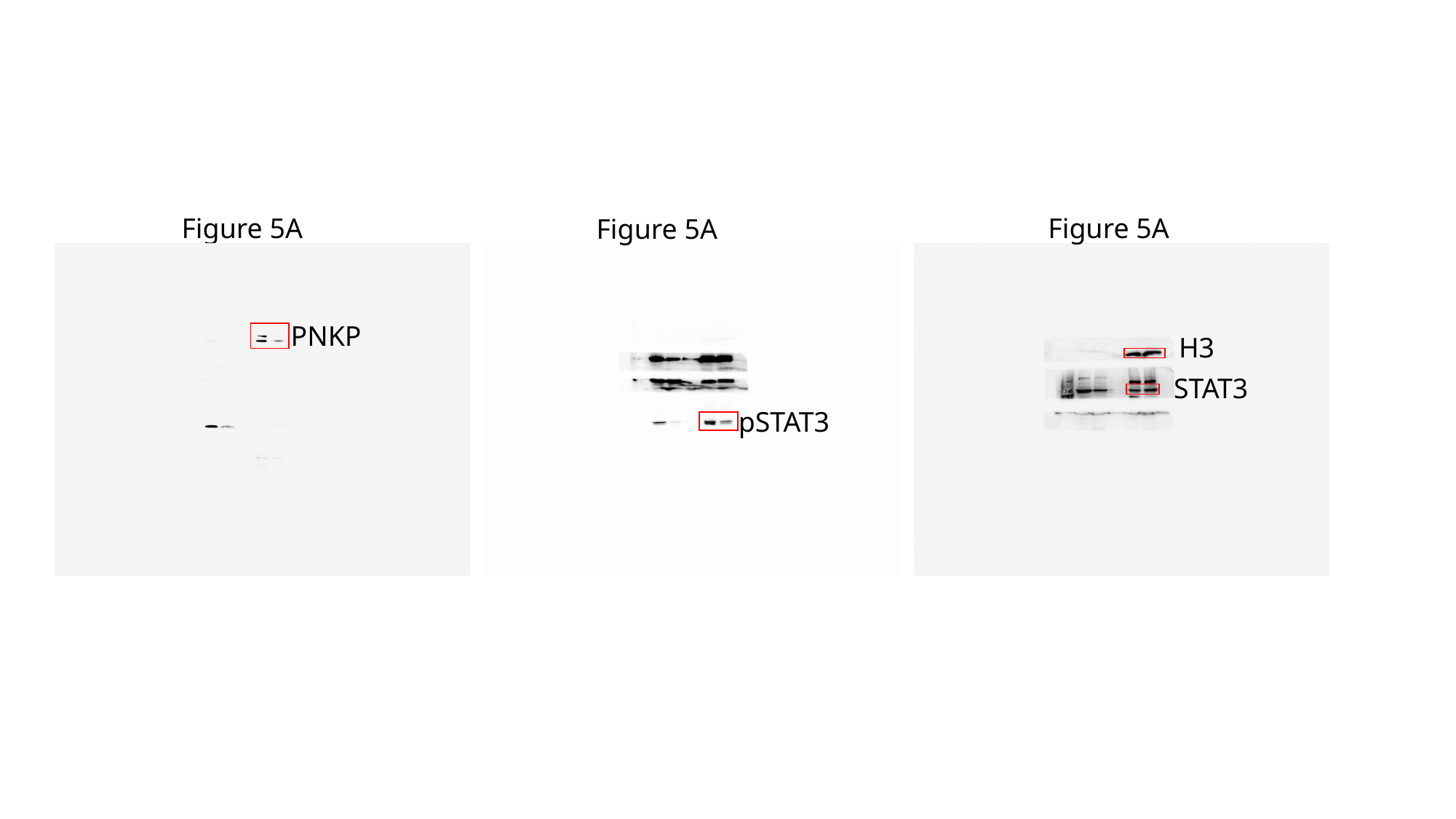

Figure 5A
Figure 5A
Figure 5A
PNKP
H3
STAT3
pSTAT3

## Slide 16
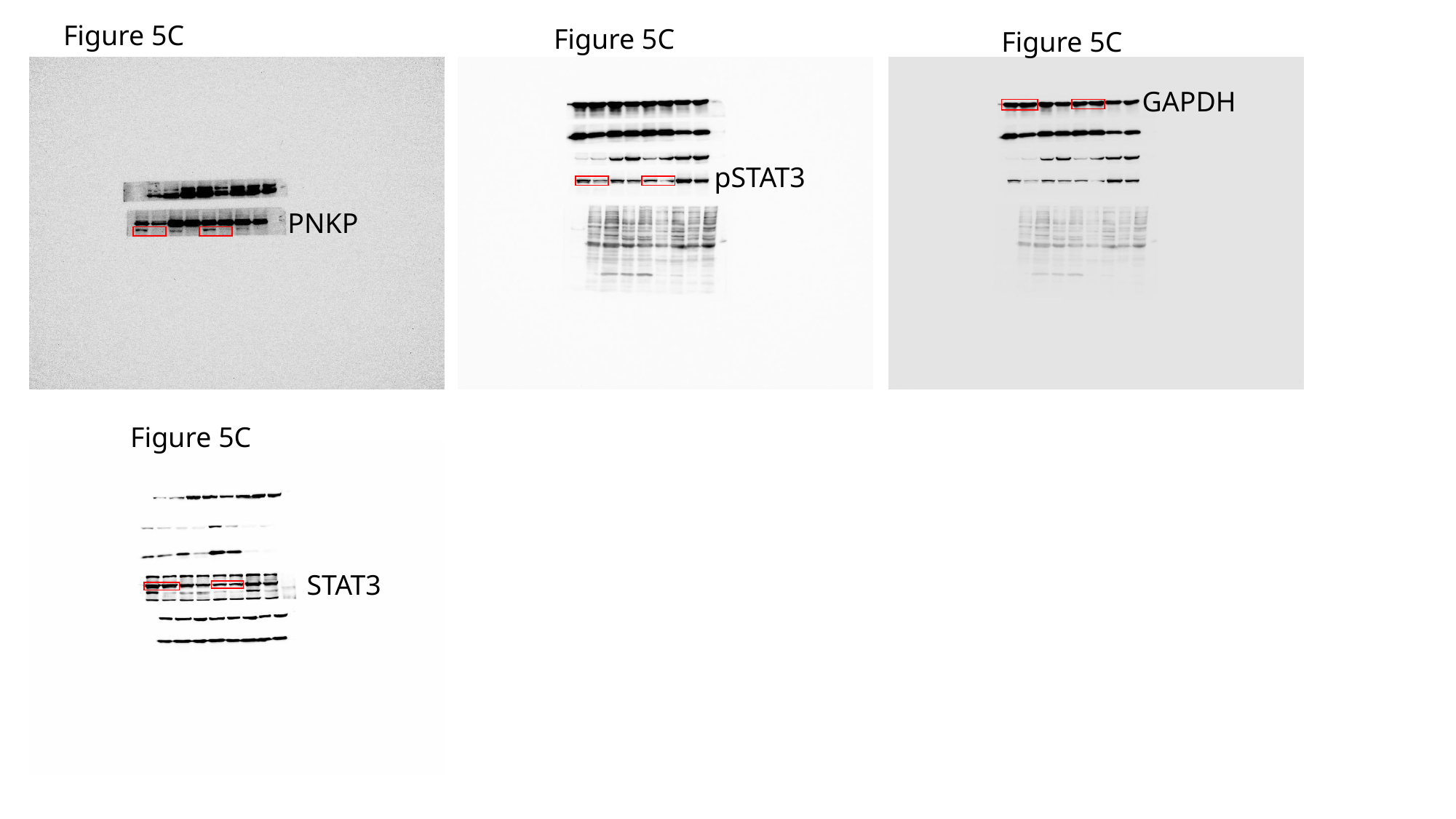

Figure 5C
Figure 5C
Figure 5C
GAPDH
pSTAT3
PNKP
Figure 5C
STAT3

## Slide 17
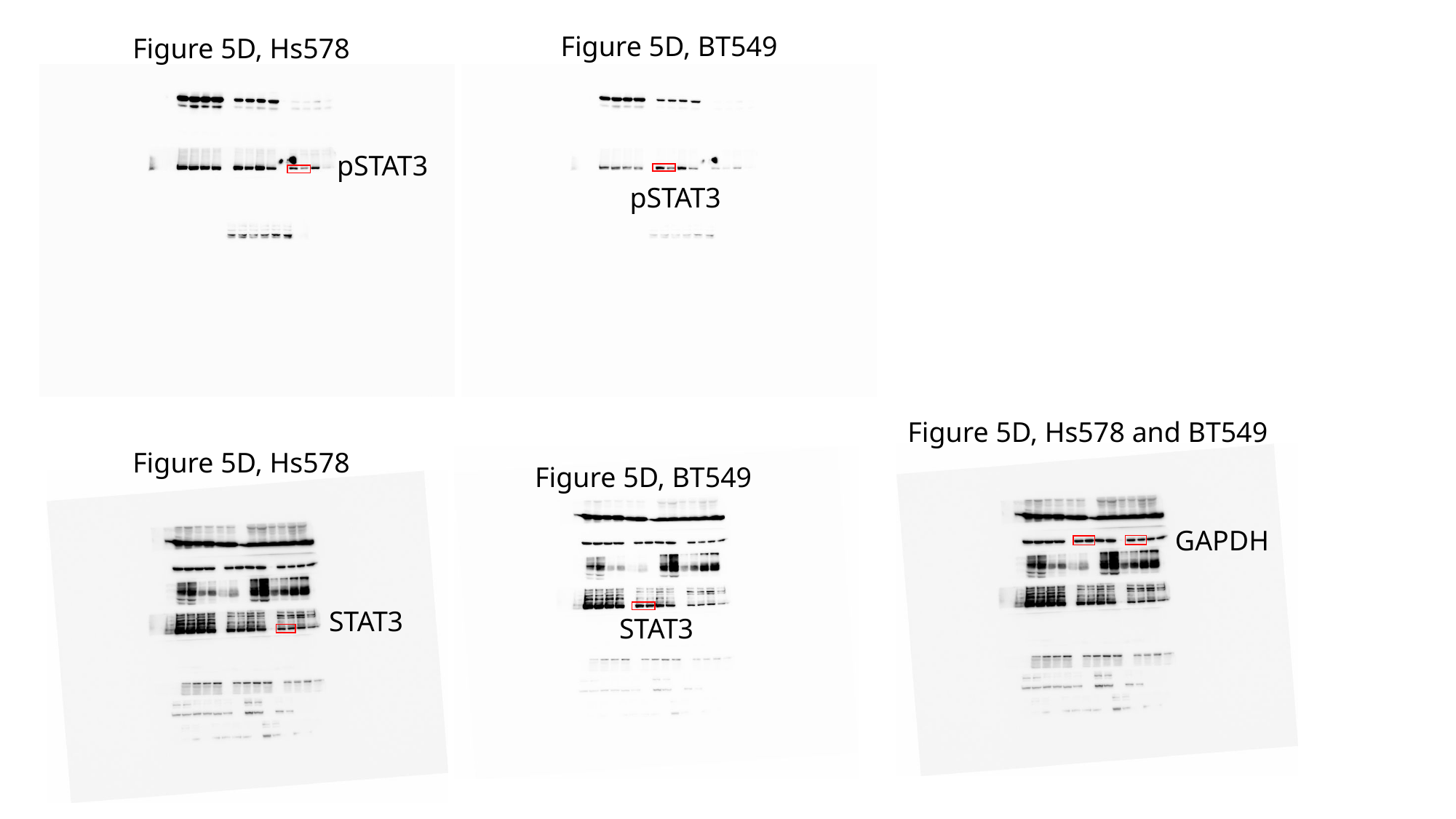

Figure 5D, BT549
Figure 5D, Hs578
pSTAT3
pSTAT3
Figure 5D, Hs578 and BT549
Figure 5D, Hs578
Figure 5D, BT549
GAPDH
STAT3
STAT3

## Slide 18
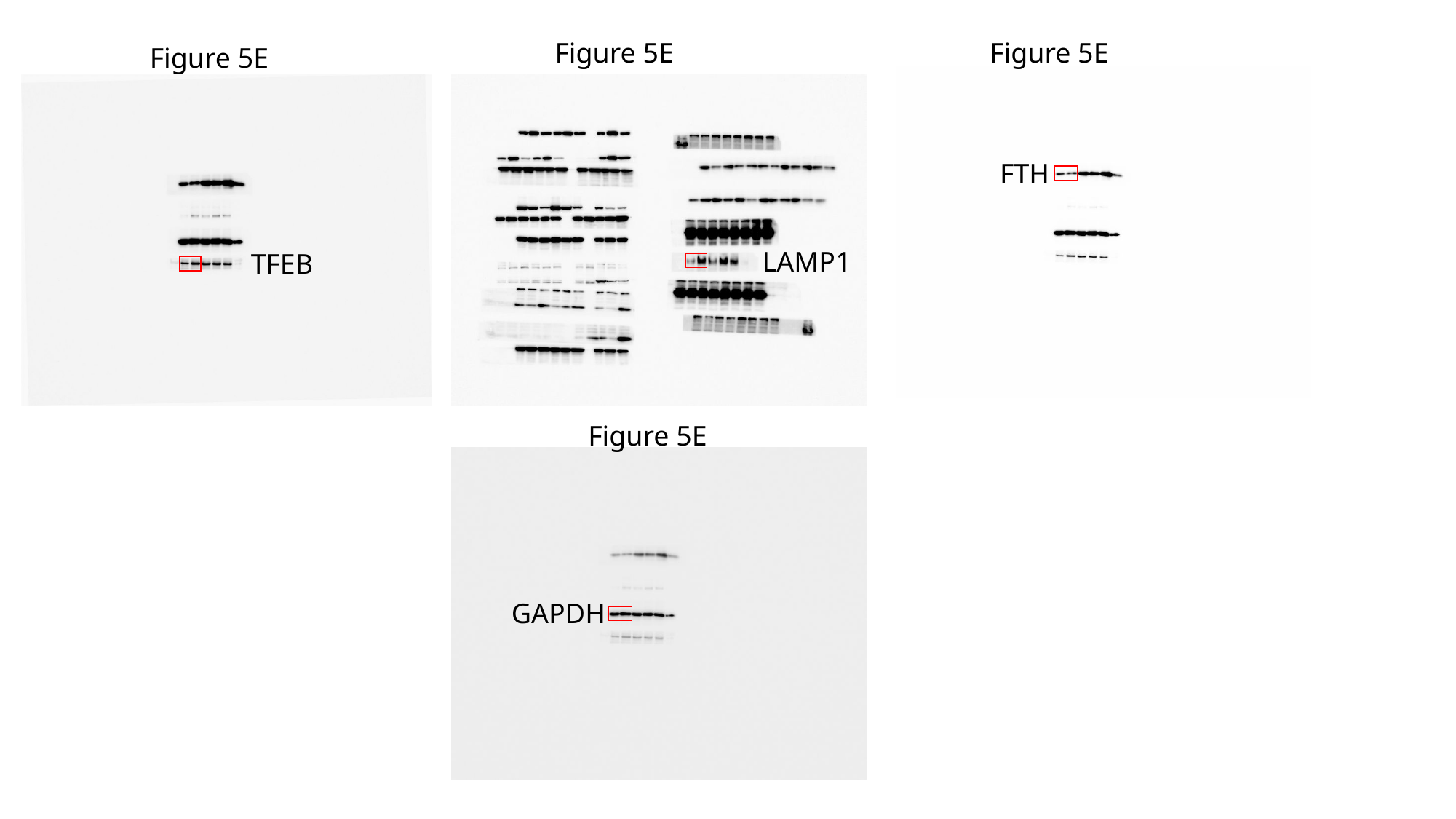

Figure 5E
Figure 5E
Figure 5E
FTH
LAMP1
TFEB
Figure 5E
GAPDH

## Slide 19
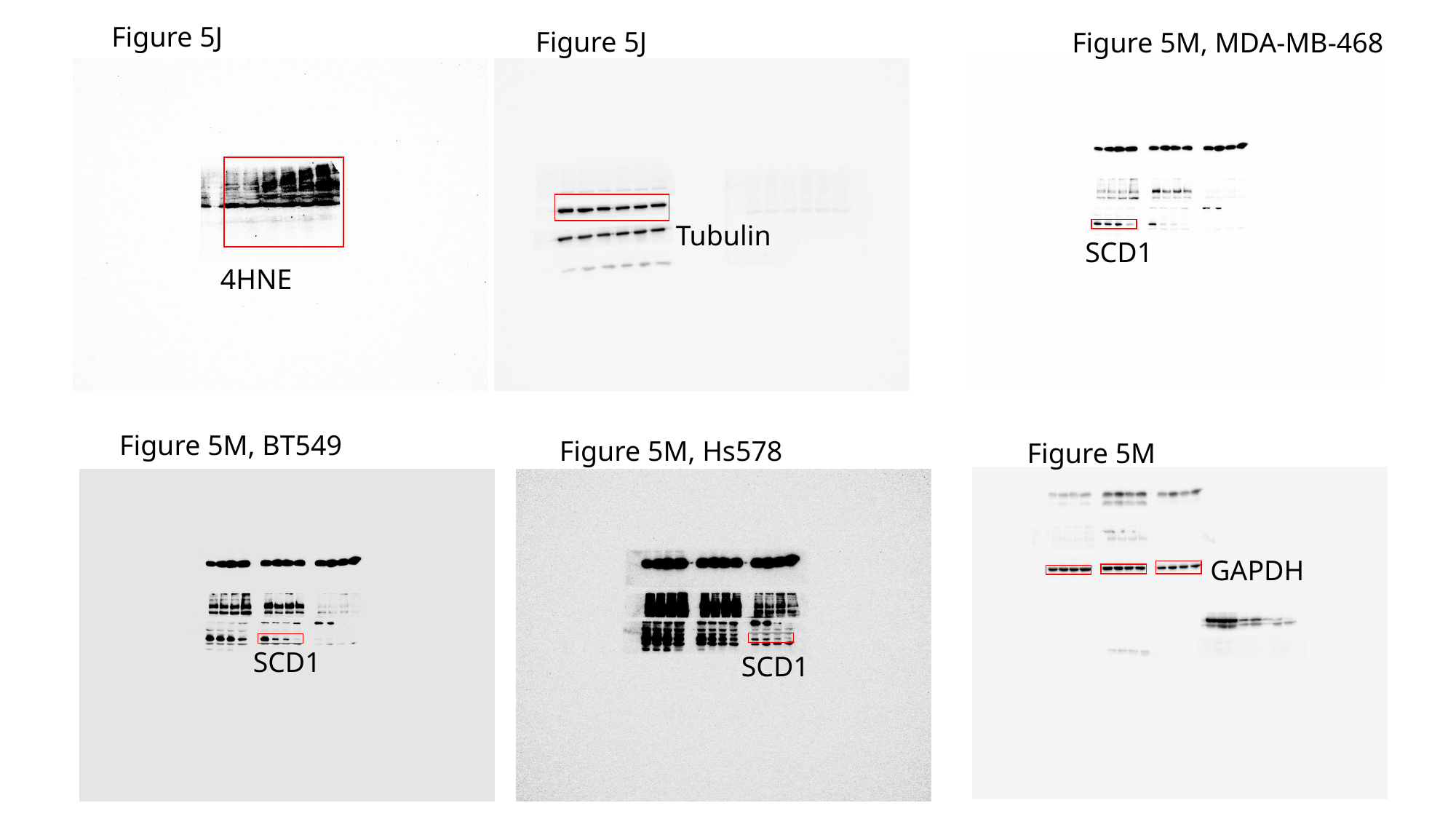

Figure 5J
Figure 5J
Figure 5M, MDA-MB-468
Tubulin
SCD1
4HNE
Figure 5M, BT549
Figure 5M, Hs578
Figure 5M
GAPDH
SCD1
SCD1

## Slide 20
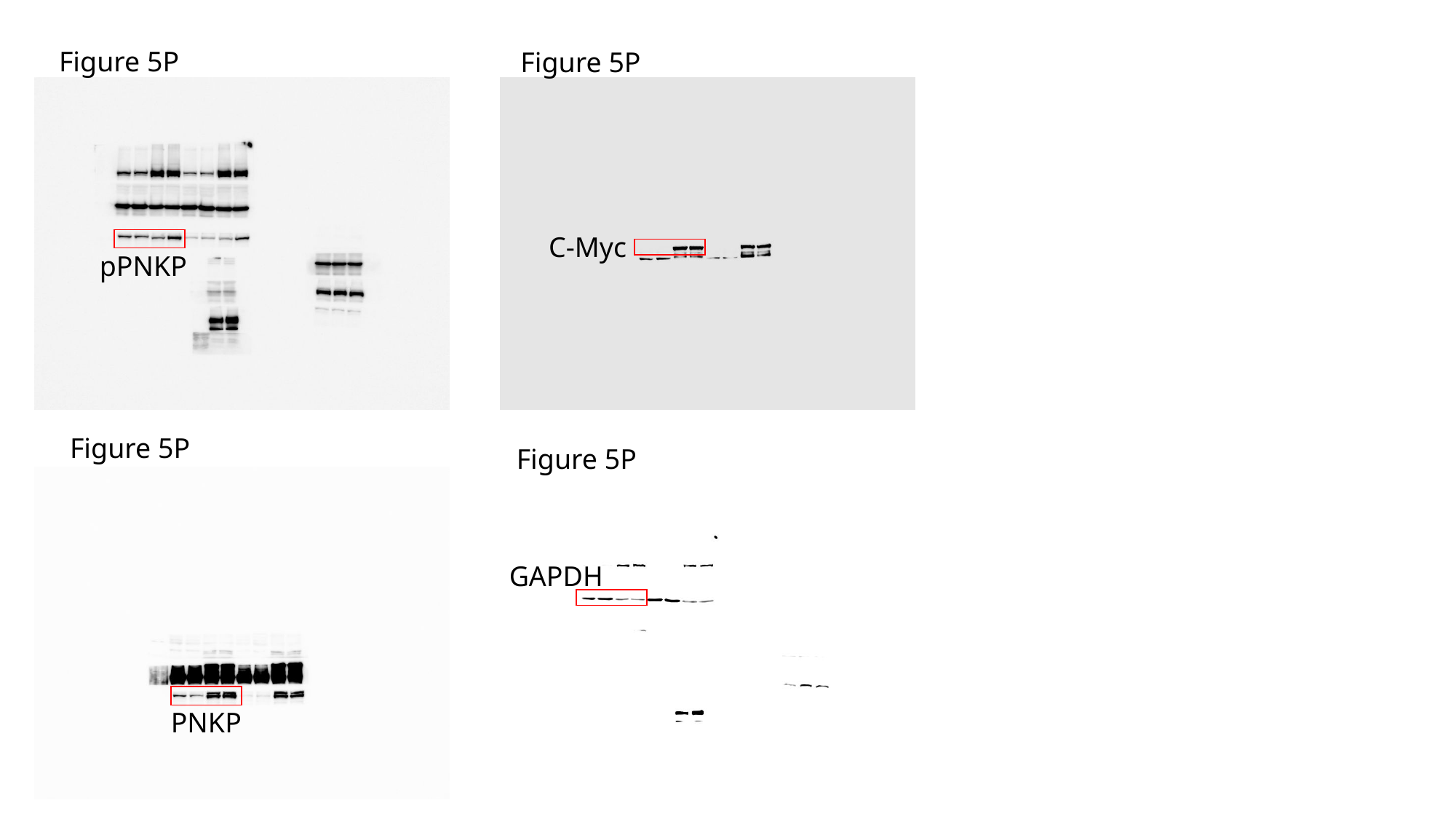

Figure 5P
Figure 5P
C-Myc
pPNKP
Figure 5P
Figure 5P
GAPDH
PNKP

## Slide 21
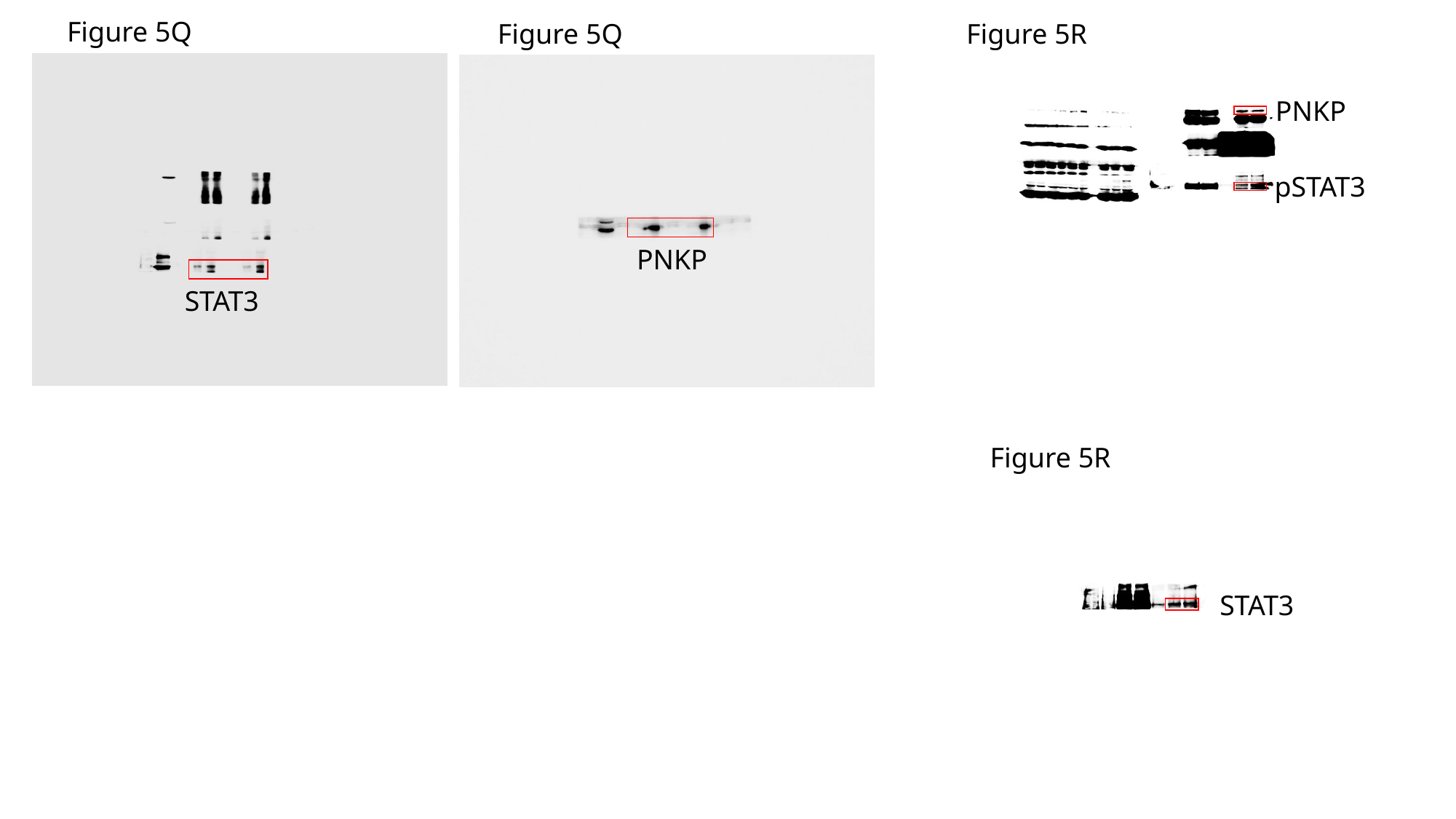

Figure 5Q
Figure 5Q
Figure 5R
PNKP
pSTAT3
PNKP
STAT3
Figure 5R
STAT3

## Slide 22
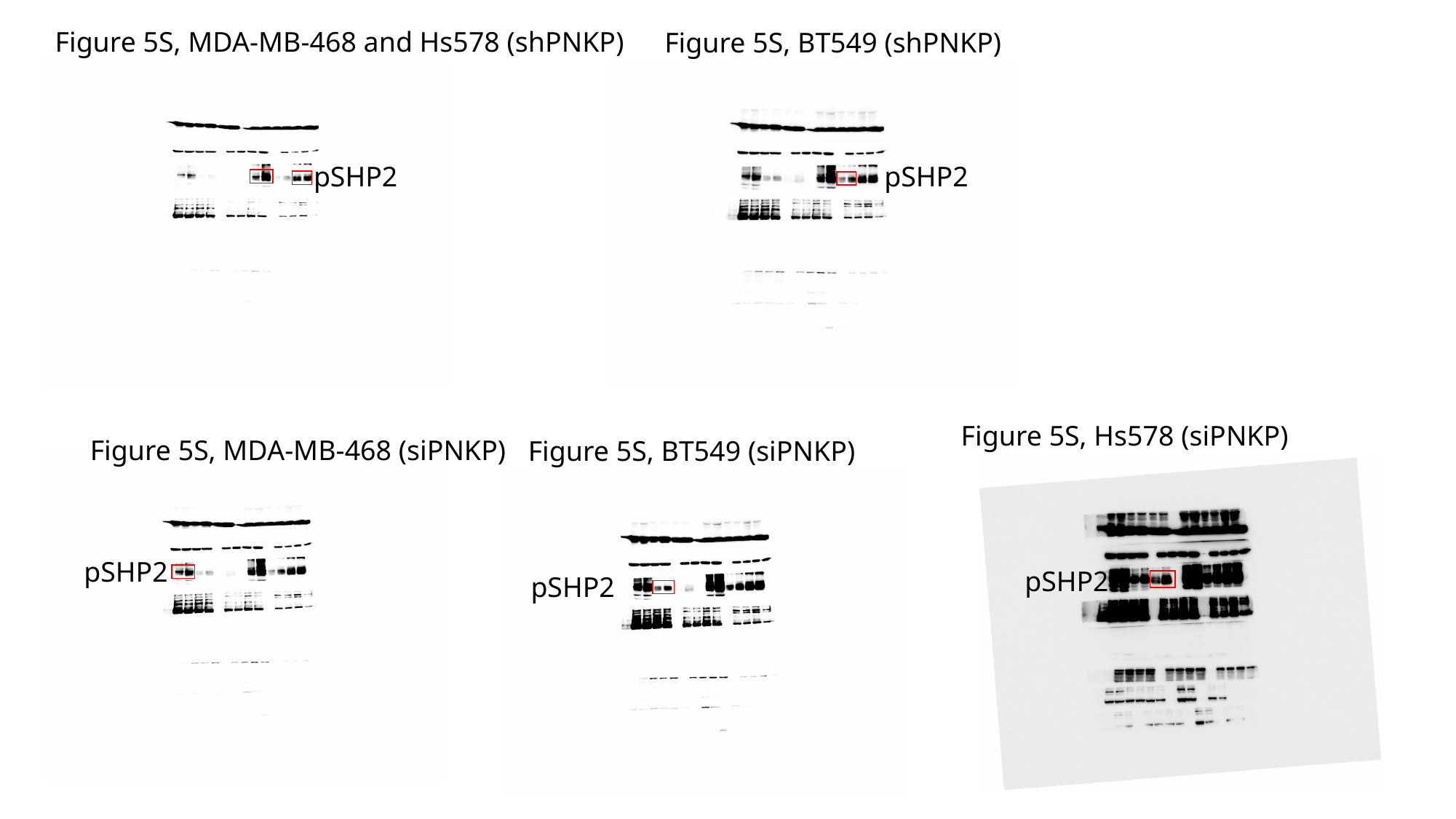

Figure 5S, MDA-MB-468 and Hs578 (shPNKP)
Figure 5S, BT549 (shPNKP)
pSHP2
pSHP2
Figure 5S, Hs578 (siPNKP)
Figure 5S, MDA-MB-468 (siPNKP)
Figure 5S, BT549 (siPNKP)
pSHP2
pSHP2
pSHP2

## Slide 23
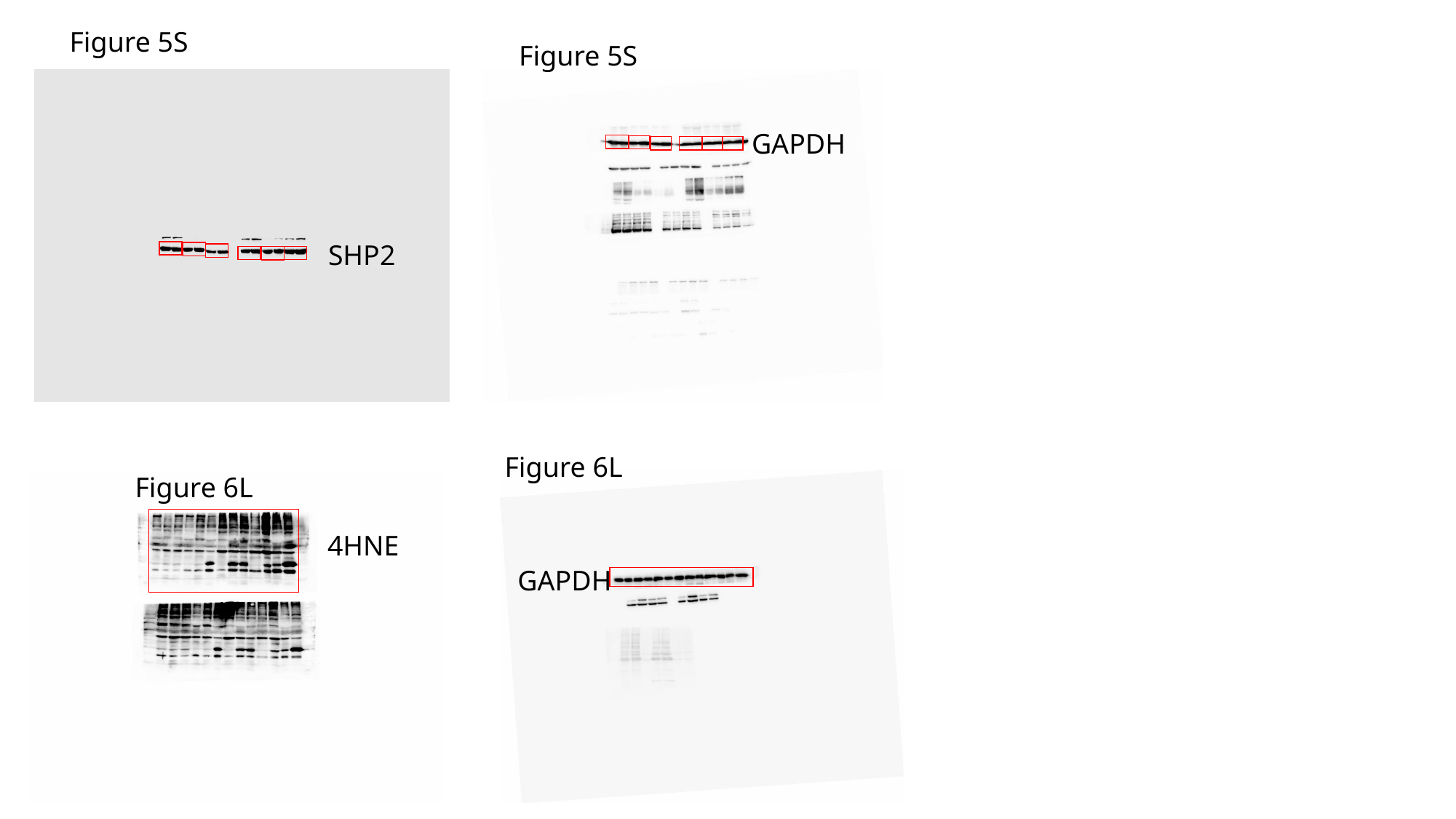

Figure 5S
Figure 5S
GAPDH
SHP2
Figure 6L
Figure 6L
4HNE
GAPDH

## Slide 24
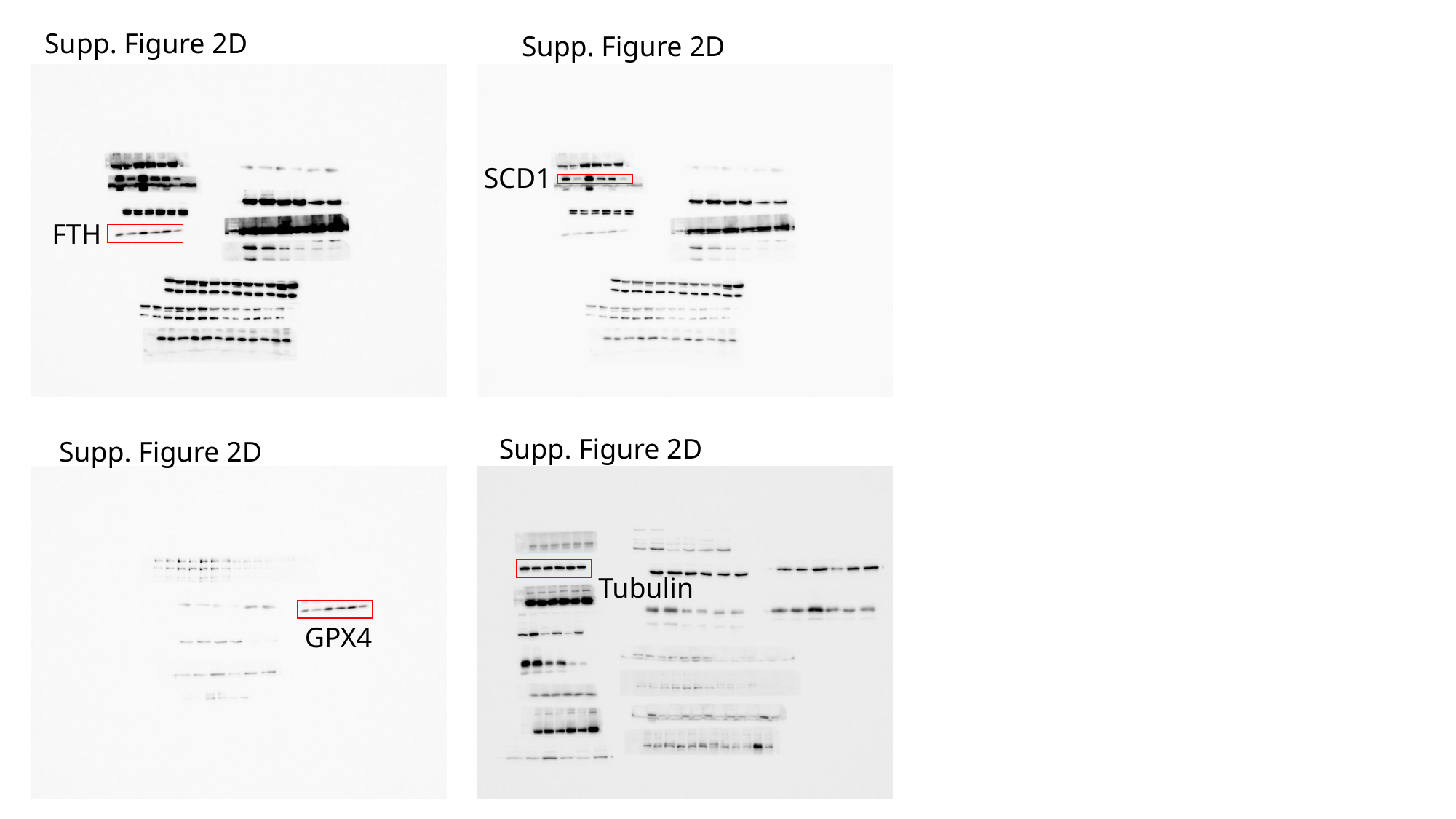

Supp. Figure 2D
Supp. Figure 2D
SCD1
FTH
Supp. Figure 2D
Supp. Figure 2D
Tubulin
GPX4

## Slide 25
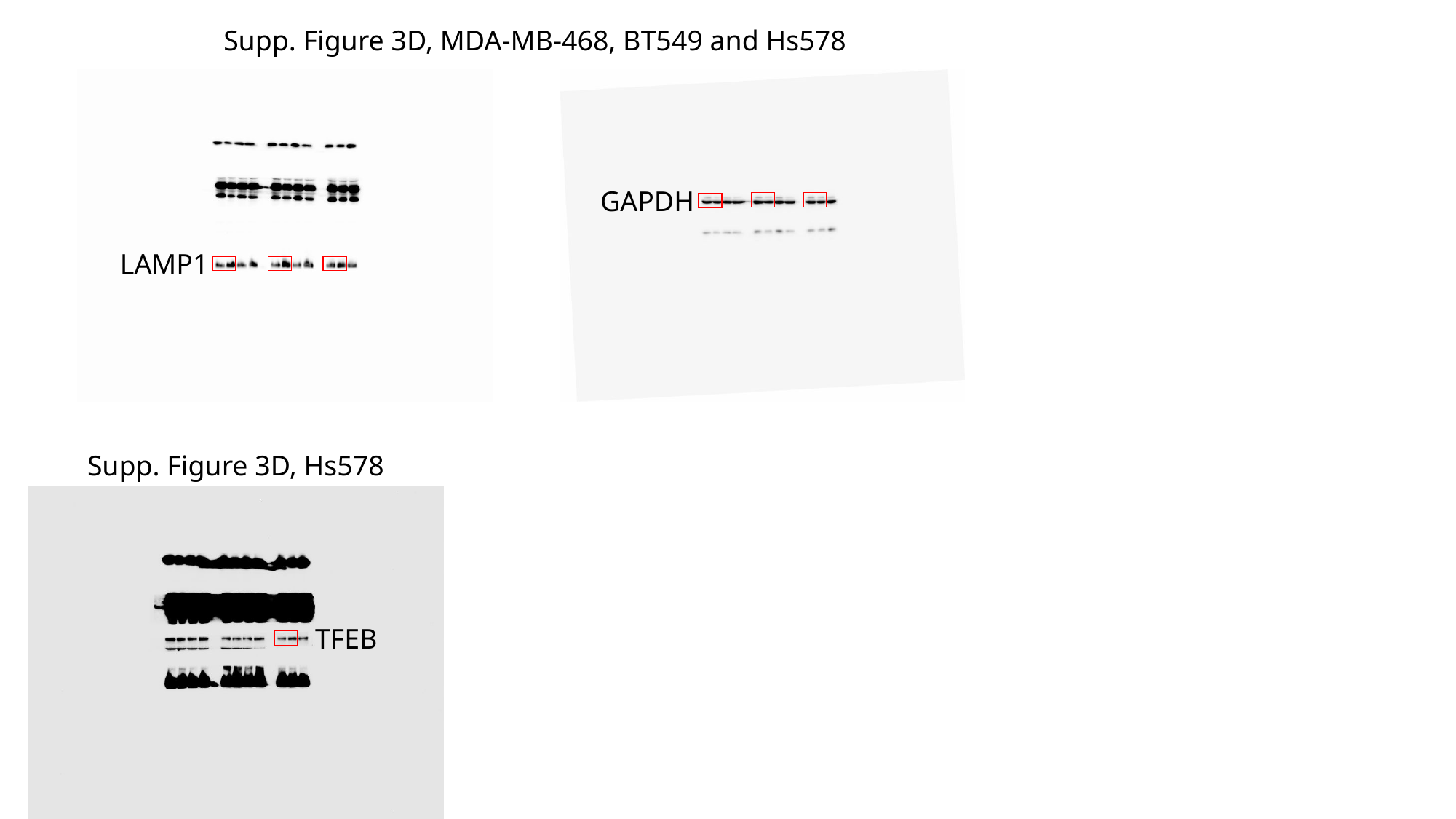

Supp. Figure 3D, MDA-MB-468, BT549 and Hs578
GAPDH
LAMP1
Supp. Figure 3D, Hs578
TFEB

## Slide 26
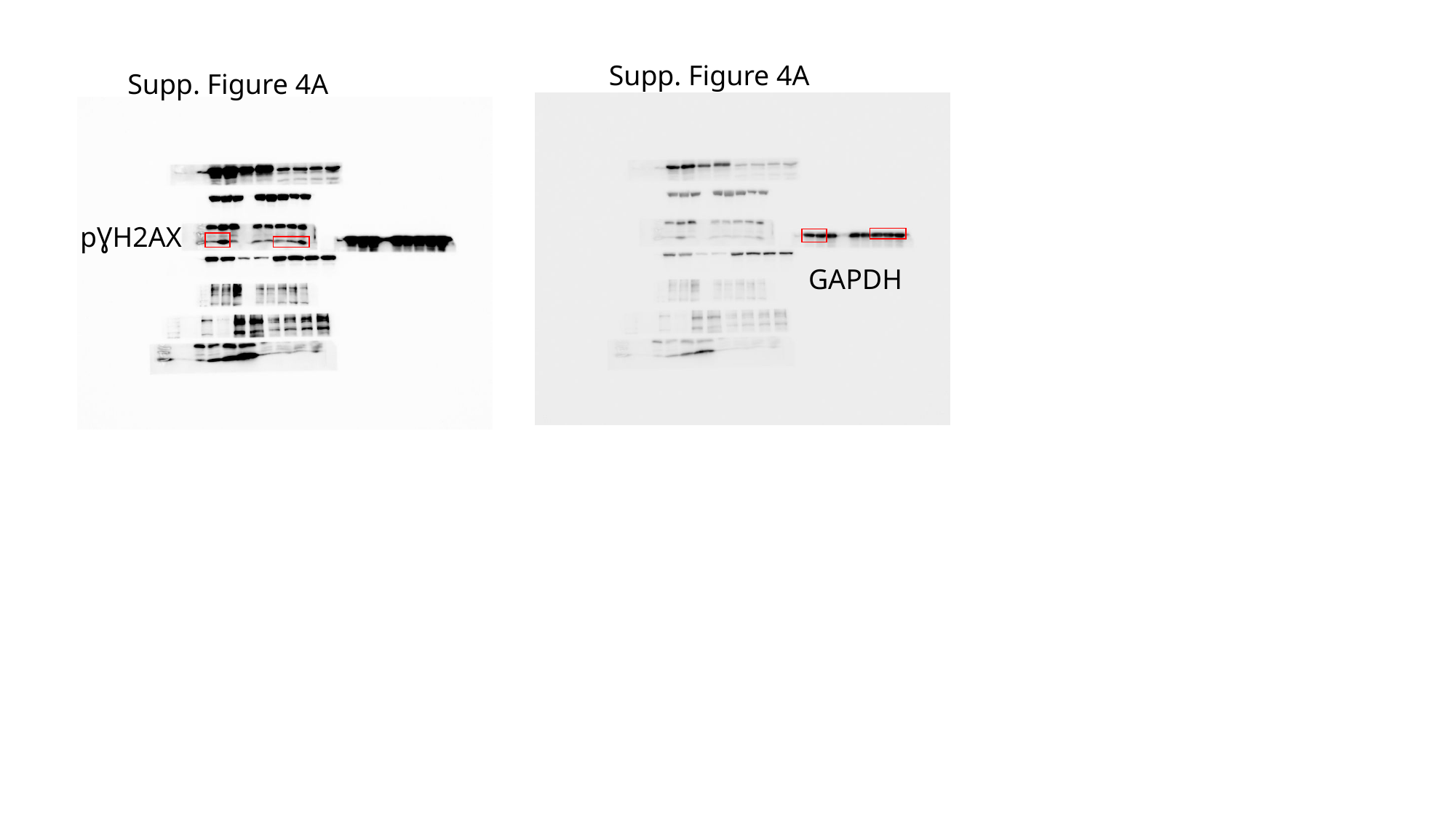

Supp. Figure 4A
Supp. Figure 4A
pƔH2AX
GAPDH

## Slide 27
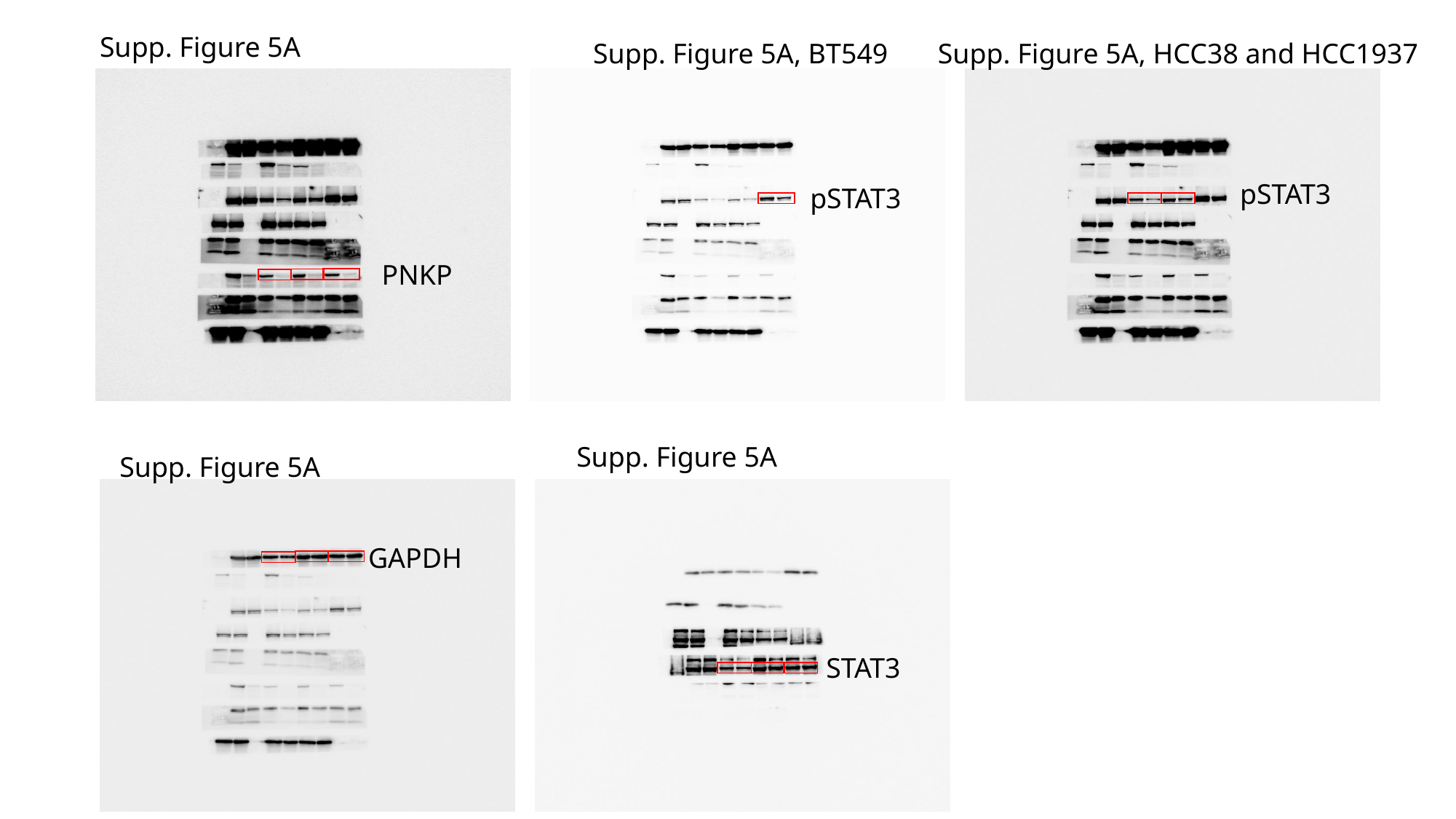

Supp. Figure 5A
Supp. Figure 5A, BT549
Supp. Figure 5A, HCC38 and HCC1937
pSTAT3
pSTAT3
PNKP
Supp. Figure 5A
Supp. Figure 5A
GAPDH
STAT3

## Slide 28
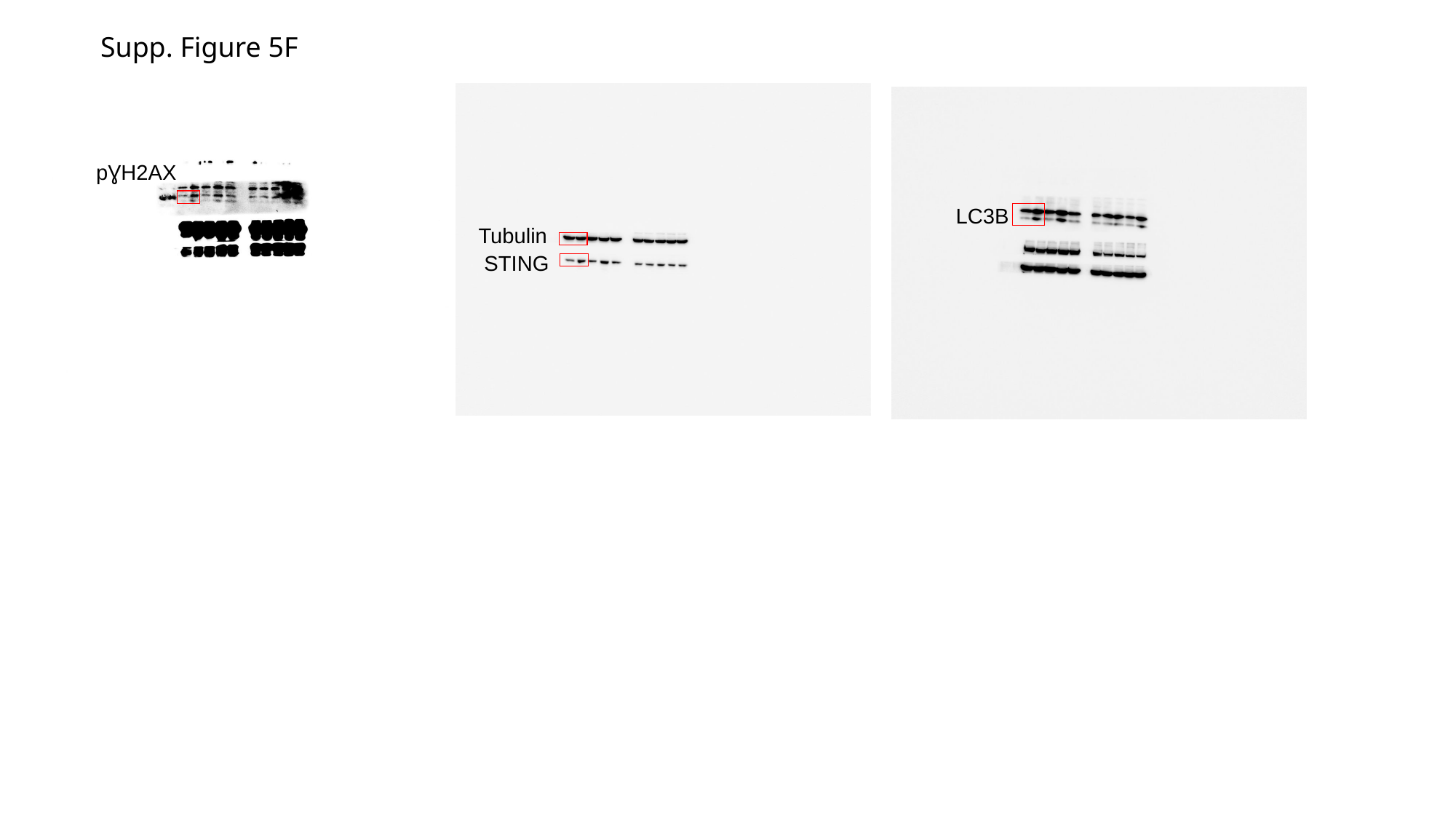

Supp. Figure 5F
pƔH2AX
LC3B
Tubulin
STING
